# Supplementary figures and images for: A new Paleogene fossil and a new dataset for waterfowl (Aves: Anseriformes) clarify phylogeny, ecological evolution, and avian evolution at the K-Pg Boundary
Source: PLoS One. 2024 Jul 30;19(7):e0278737. doi: 10.1371/journal.pone.0278737 (PMC11288464; doi:10.1371/journal.pone.0278737)

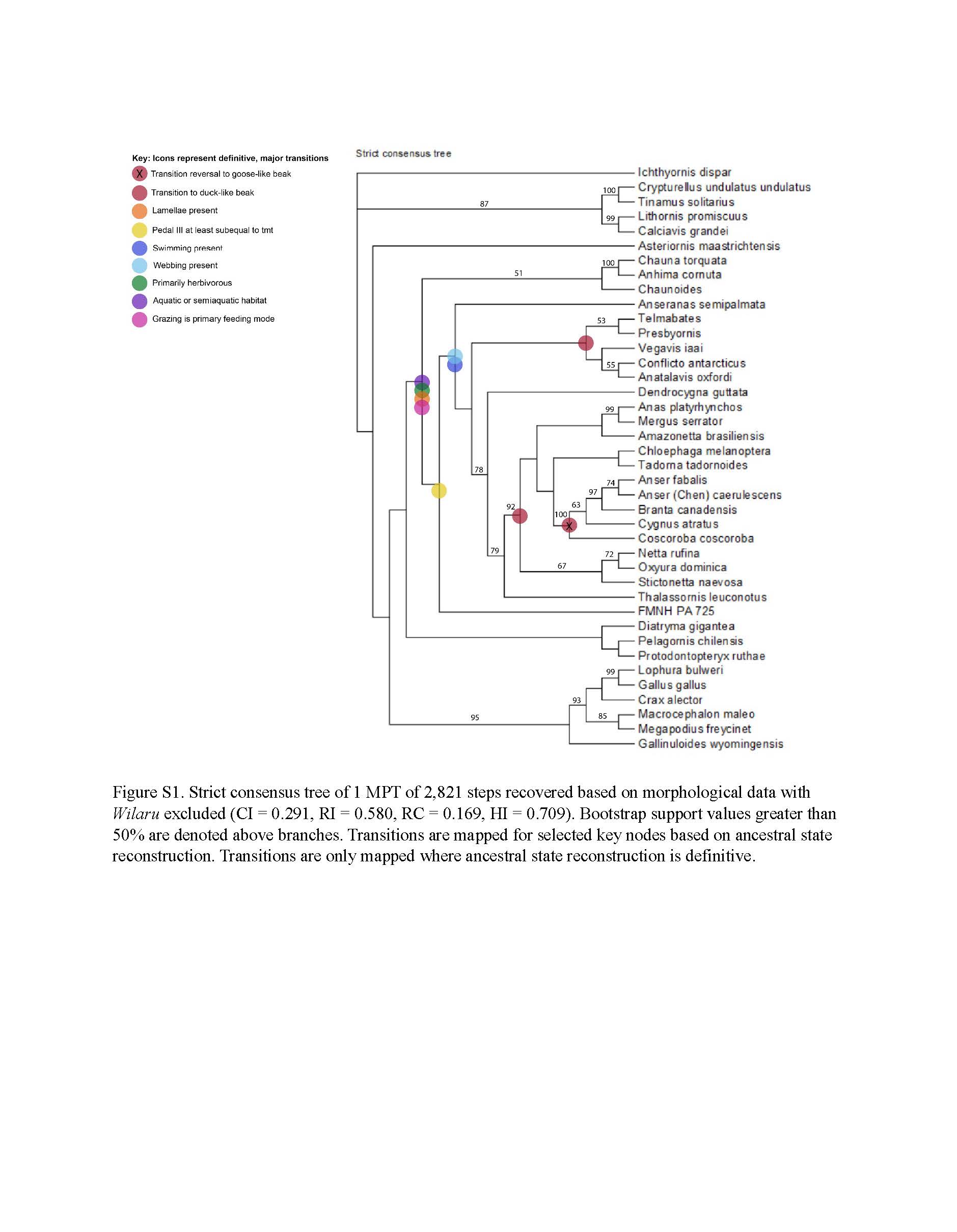

Supplement: S1 Fig — Strict consensus tree of 1 MPT of 2,821 steps recovered based on morphological data with Wilaru excluded (CI = 0.291, RI = 0.580, RC = 0.169, HI = 0.709). Bootstrap support values greater than 50% are denoted above branches. Transitions are mapped for selected key nodes based on ancestral state reconstruction. Transitions are only mapped where ancestral state reconstruction is definitive. (JPG) [file pone.0278737.s001.jpg]

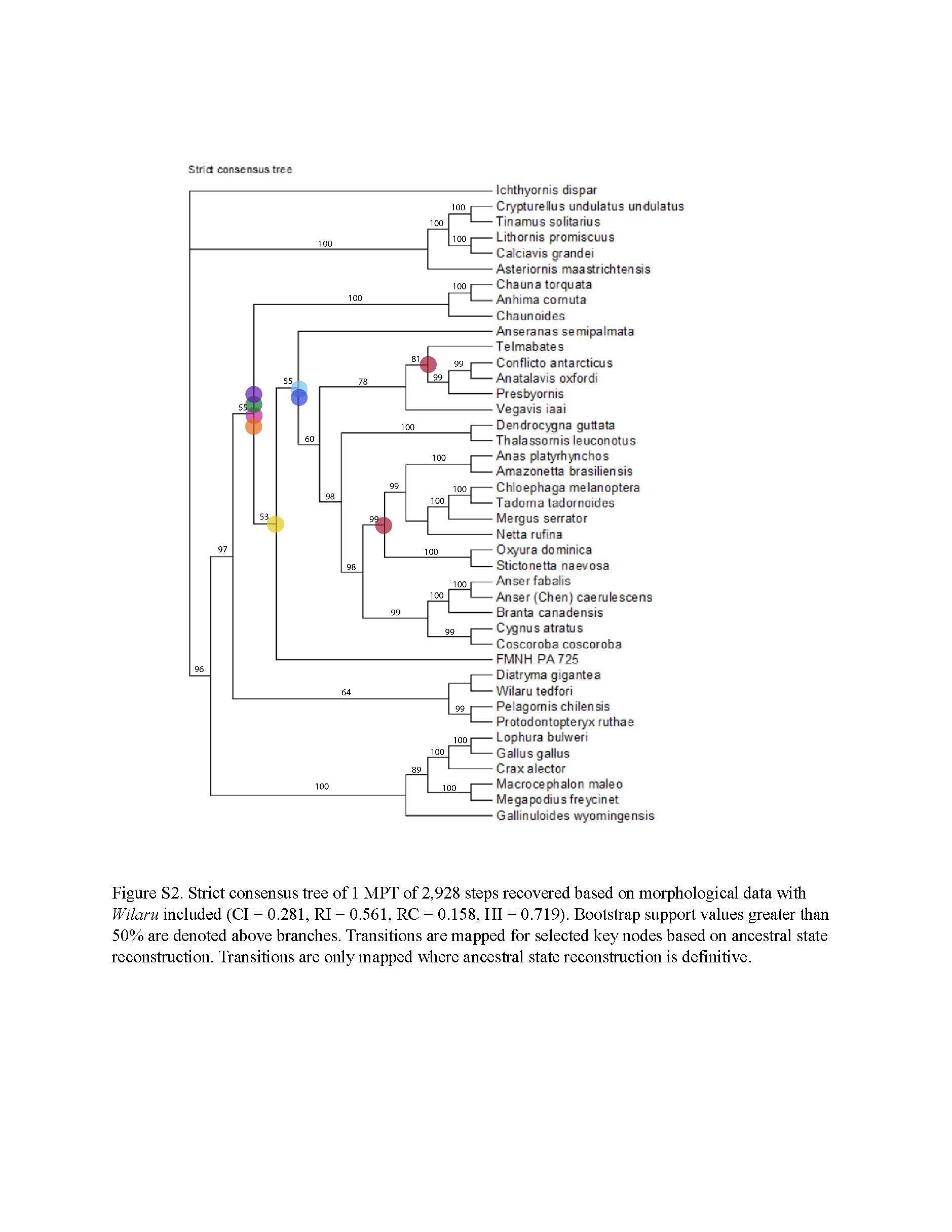

Supplement: S2 Fig — Bootstrap support values greater than 50% are denoted above branches. Transitions are mapped for selected key nodes based on ancestral state reconstruction. Transitions are only mapped where ancestral state reconstruction is definitive. (JPG) [file pone.0278737.s002.jpg]

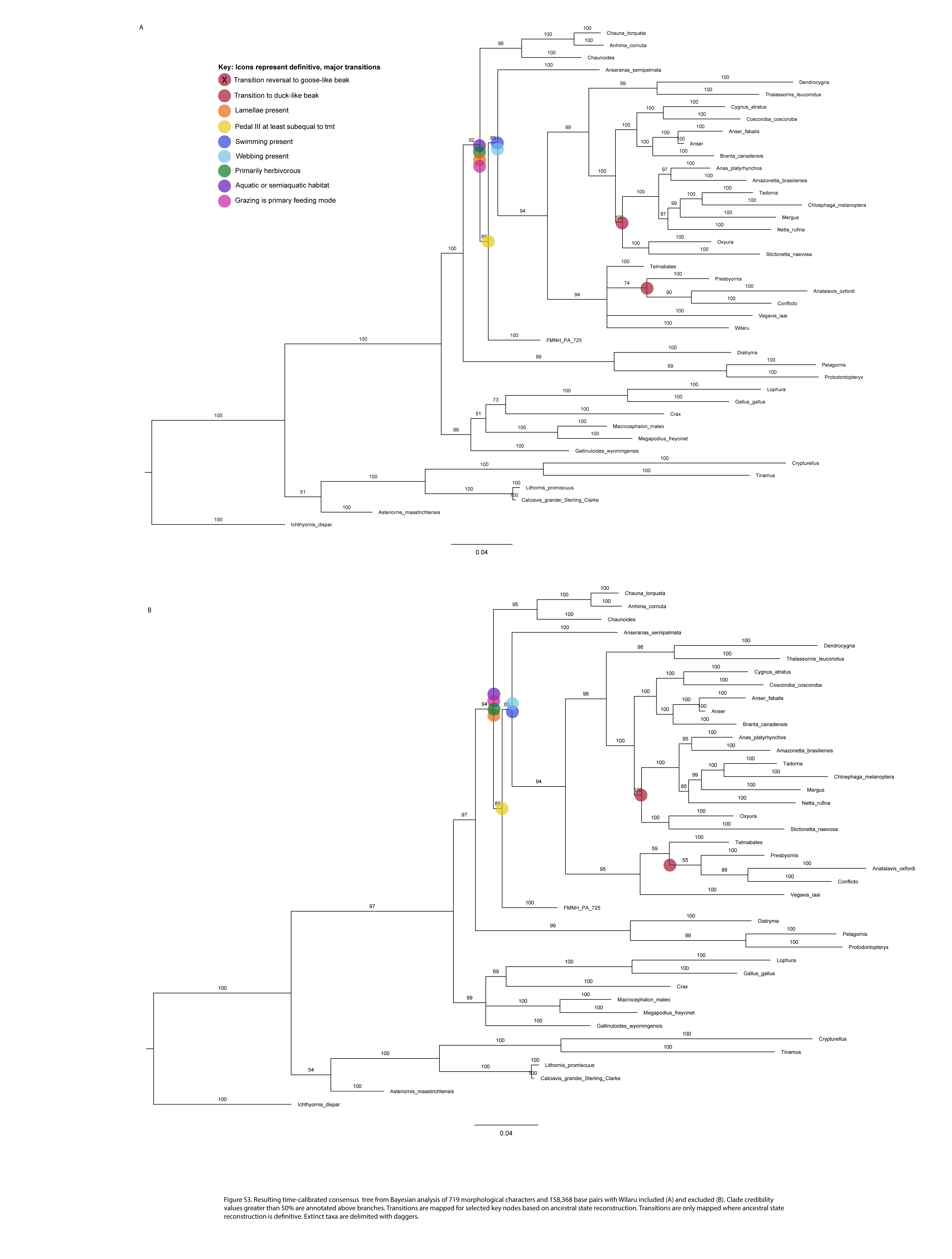

Supplement: S3 Fig — Clade credibility values greater than 50% are annotated above branches. Transitions are mapped for selected key nodes based on ancestral state reconstruction. Transitions are only mapped where ancestral state reconstruction is definitive. Extinct taxa are delimited with daggers. (TIF) [file pone.0278737.s003.tif]

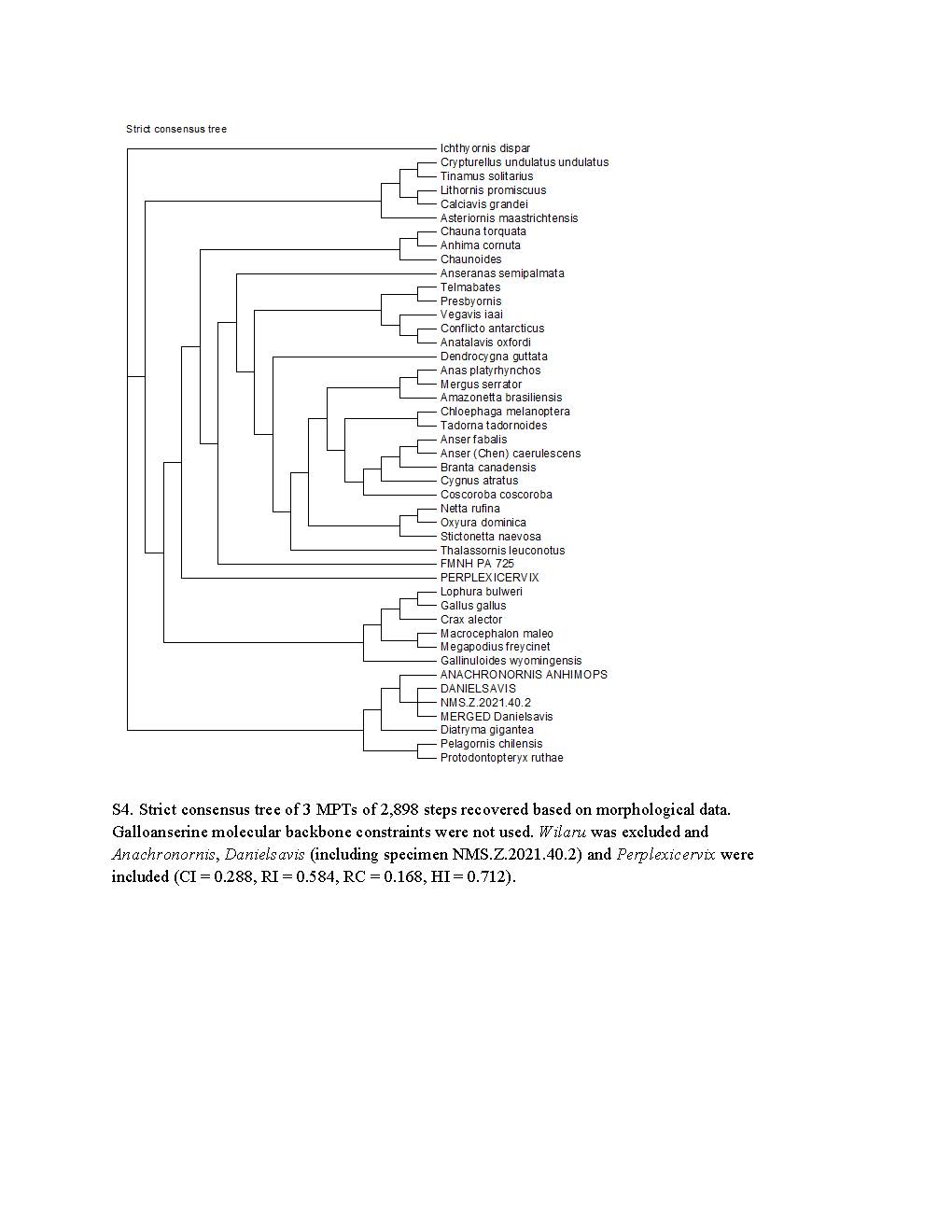

Supplement: S4 Fig — Galloanserine molecular backbone constraints were not used. Wilaru was excluded and Anachronornis, Danielsavis (including specimen NMS.Z.2021.40.2) and Perplexicervix were included (CI = 0.288, RI = 0.584, RC = 0.168, HI = 0.712). (JPG) [file pone.0278737.s004.jpg]

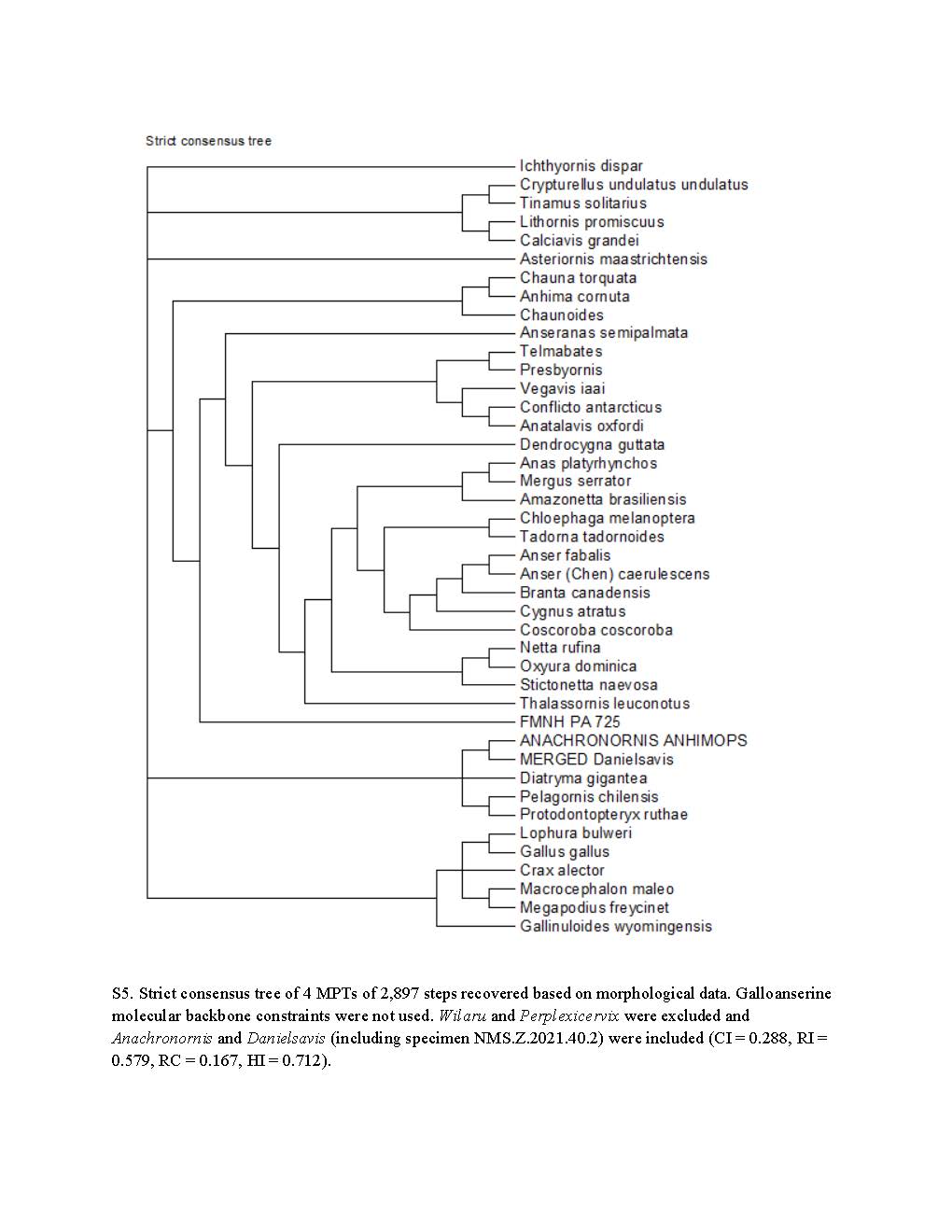

Supplement: S5 Fig — Galloanserine molecular backbone constraints were not used. Wilaru and Perplexicervix were excluded and Anachronornis and Danielsavis (including specimen NMS.Z.2021.40.2) were included (CI = 0.288, RI = 0.579, RC = 0.167, HI = 0.712). (JPG) [file pone.0278737.s005.jpg]

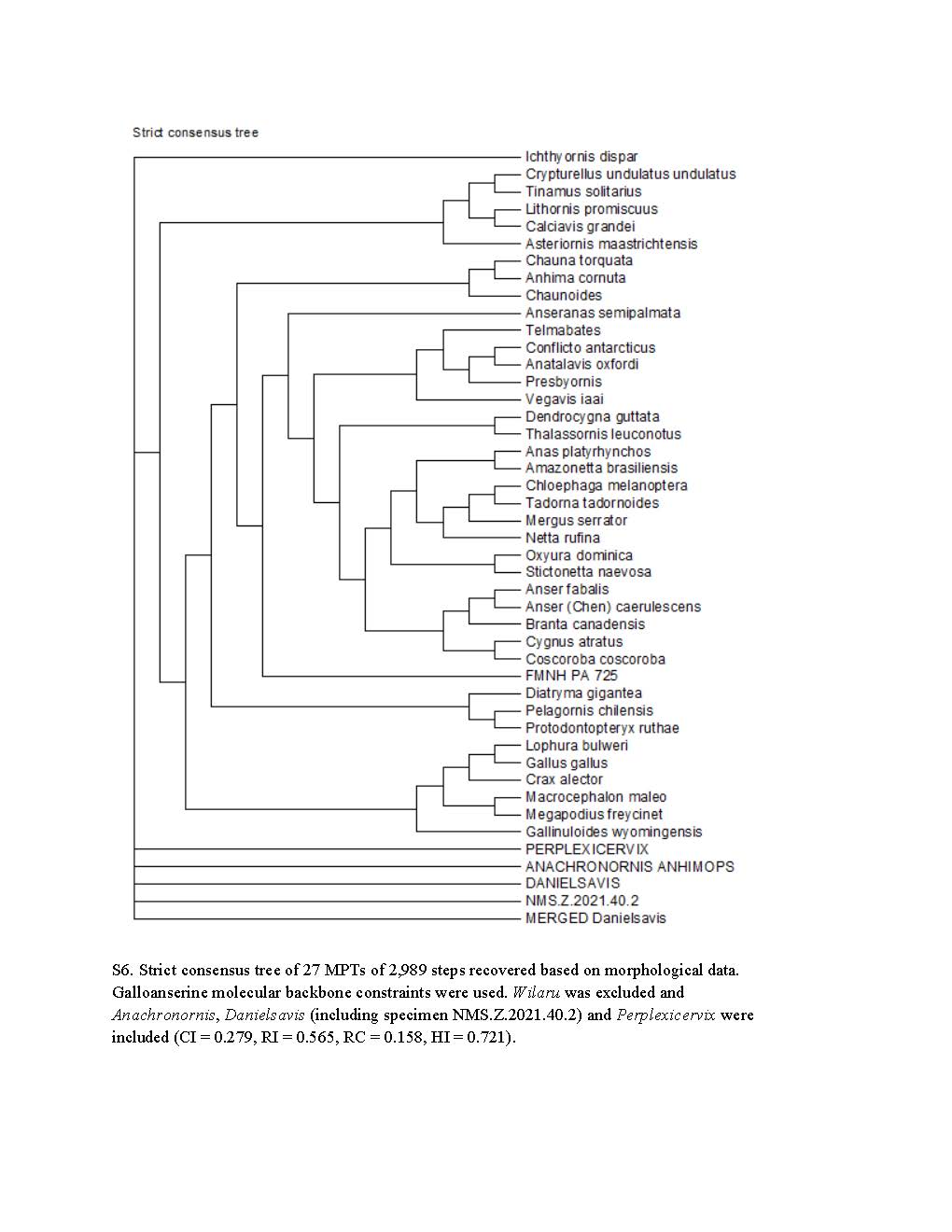

Supplement: S6 Fig — Galloanserine molecular backbone constraints were used. Wilaru was excluded and Anachronornis, Danielsavis (including specimen NMS.Z.2021.40.2) and Perplexicervix were included (CI = 0.279, RI = 0.565, RC = 0.158, HI = 0.721). (JPG) [file pone.0278737.s006.jpg]

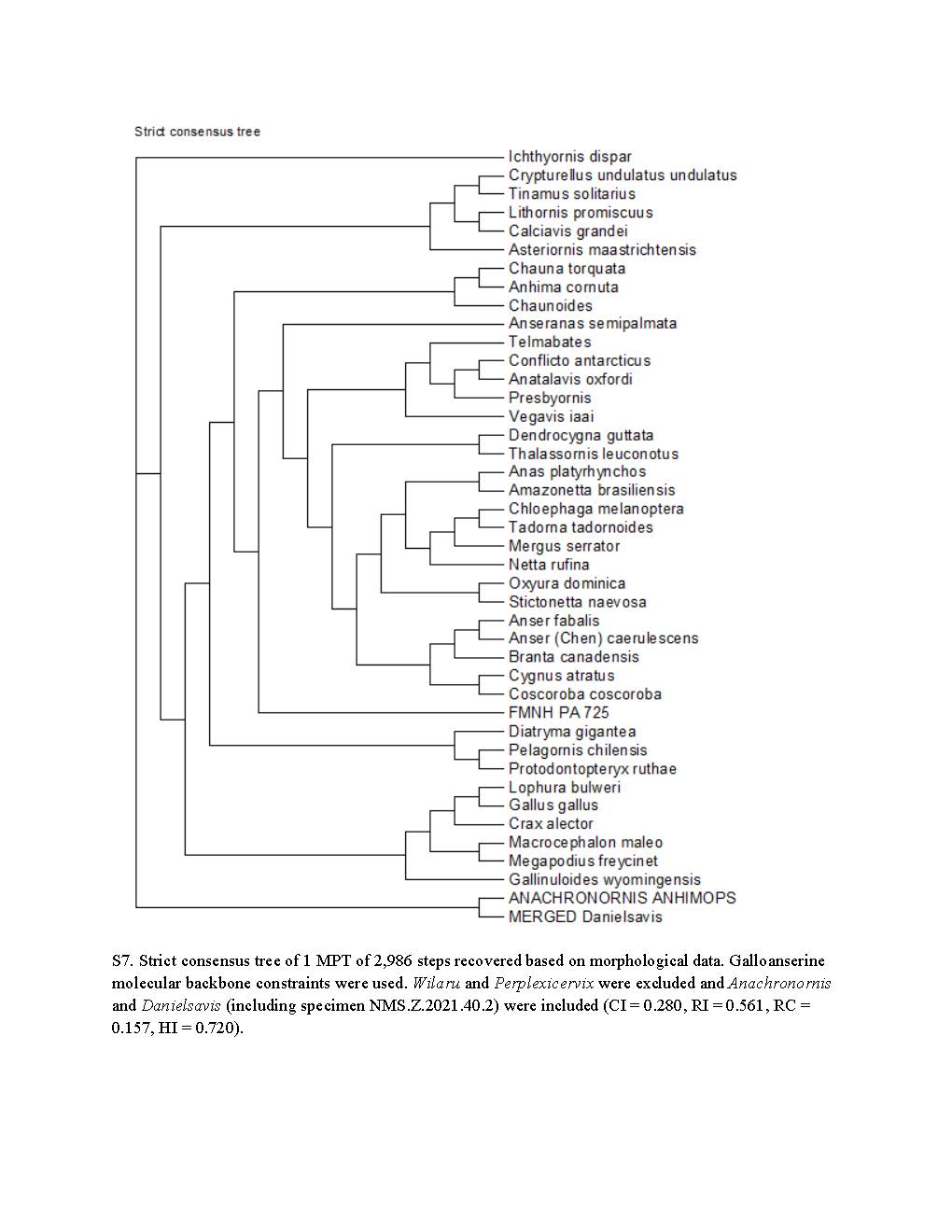

Supplement: S7 Fig — Galloanserine molecular backbone constraints were used. Wilaru and Perplexicervix were excluded and Anachronornis and Danielsavis (including specimen NMS.Z.2021.40.2) were included (CI = 0.280, RI = 0.561, RC = 0.157, HI = 0.720). (JPG) [file pone.0278737.s007.jpg]
