# Supplementary material for: A new Paleogene fossil and a new dataset for waterfowl (Aves: Anseriformes) clarify phylogeny, ecological evolution, and avian evolution at the K-Pg Boundary
Source: PLoS One. 2024 Jul 30;19(7):e0278737. doi: 10.1371/journal.pone.0278737 (PMC11288464; doi:10.1371/journal.pone.0278737)
Supplement: S1 Appendix — (DOCX) [file pone.0278737.s008.docx]

Appendix Character Descriptions

State (0) is reserved for absence. Citations are provided where significant overlap with a previously created character has occurred and/or or where the character has been modified based on assessment of previously created characters. Citations of previously created characters are not meant to represent a comprehensive list of character overlap, and largely comprise the characters (and citations therein) of Musser and Cracraft [77] and Musser and Clarke [83] due to our building on this dataset. Characters are anglicized as much as possible.

Skull: Rostrum

1. Premaxilla, dorsal aspect of rostral apex, distinct pair of foramina: absent (0); present (1); present, with associated rostral sulcus (2). Musser and Clarke [83], character 1; Musser and Clarke [77], character 1.
2. Premaxilla, rostral apex, curvature: slightly curved inferiorly (1); markedly curved inferiorly, hooked (2); lacking inferior curvature so that bill appears to be flat or recurved superiorly (3). Musser and Clarke [83], character 2; Musser and Clarke [77], character 4.
3. Rostrum, ventral aspect, caudal mediolateral width equal to or broader than width across the paroccipital processes: no (1); yes, subequal or rostrum is wider through anterior portion (2); yes, subequal or rostrum is wider at base but is slender toward anterior portion (3).
4. Rostrum, if as wide or wider than width across paroccipital processes, width of anterior portion of rostrum: tapered (1); subequal (2); further expanded laterally (3). Noncomparable where rostrum is mediolaterally less wide than the width across the paroccipital processes.
5. Rostrum, if as wide or wider than width across paroccipital processes, dorsal thickening of dorsomedial margin of rostral apex: absent (0); present (1). Noncomparable where rostrum is mediolaterally less wide than the width across the paroccipital processes.
6. Rostrum, so extensively fused that nares are small, slit-like and appear non-perforate: no (1); yes (2).
7. Maxilla, anterocaudal length along ventral rostrum compared to that of premaxilla: maxilla makes up approximately ¾ of premaxilla (1); ½ of premaxilla (2); 1/3 or less of rostrum (3).
8. Premaxilla, processus frontalis, caudal terminus and associated suture between premaxilla and nasals: triangular (1); rounded (2). Noncomparable where suture obscured by nasal and/or frontal. Musser and Clarke [83], character 5.
9. Rostrum, curvature along ventral margin, not including anterior terminus of premaxilla: lacking inferior curvature so that bill appears to be flat or recurved superiorly (1); curved inferiorly (2); curved extremely inferiorly, distal half of rostrum essentially perpendicular to proximal half (3); distal half of rostrum recurved dorsally (4). Musser and Clarke [83], character 6.
10. Rostrum, ventral aspect, tomial crest, caudomedial extent and ventral enclosure of rostrum and consequent shape of ventromedial fenestra: almost completely fused along midline to border of antorbital angle so that rostrum essentially closed by tomial crest ventrally, slit-like fenestra along midline may be visible in ventral aspect (1); fused along about 1/2 of midline or less near rostral apex so that pair of cylindrical cavities at apex visible but separated toward antorbital fenestra, creating "leaf-shaped" fenestra that is wider near antorbital fenestra (2); ventral

enclosure of rostrum lost completely (3); like state 2 but over 1/2 of rostral portion is enclosed, pair of concavities concealed by tomial crest (4); completely closed ventrally by inflated bone

(5). Musser and Clarke [83], character 8.

1. Rostrum, tomial crest, lateral margins, ventral extension: essentially none, tomial crest at same level of ventral face of rostrum (1); marked caudal to apex (2); marked at rostral apex (3). Not applicable if rostrum not closed ventrally by tomial crest. Musser and Clarke [83], character 9.
2. Lamellae for filter feeding: absent (0); present, vestigial (1); present, well developed (2). Mayr and Clarke [81], character 3; Worthy et al. [27], character 6.
3. Nares, length as compared to total length of rostrum: less than 1/2 of rostrum (1); 1/2 of rostrum (2); over 1/2 length of rostrum (3). Musser and Clarke [83], character 14.
4. Rostrum, lateral aspect, tomial margin ventral to nares, dorsovental width at midpoint of nares compared to dorsoventral width of dorsal narial bar: narrow, subequal (1); wide, at least three times the width of the dorsal narial bar (2).
5. Rostrum, lateral aspect, nasal bar, anterocaudal width at dorsoventral midpoint of caudal portion of nares: narrow, mediolateral width subequal to dorsoventral width of dorsonarial bar

(1); wide, at least twice width (2).

1. Premaxilla, length facies apertura nasalis compared to width craniofacial hinge, which in Palaeognathae is interpreted as the rostral side of lacrimal: long, greater than width of craniofacial hinge (1); intermediate, subequal (2); short, much less than width craniofacial hinge
2. Worthy et al. [27], character 2.
3. Nares, nasal sulcus beginning from rostral limit of nares: present, complete to terminal end of rostrum (1); lost or truncated (2). Musser and Clarke [83], character 12; Musser and Clarke [77],

character 9; Livezey and Zusi [88], character 272.

1. Nares, nasal sulcus beginning from rostral limit of nares, depth and magnitude: shallow or barely visible (1); extremely deep and elongate, length over half of rostrum (2). Noncomparable where sulcus absent.
2. Nares, rostral margin: rounded (1); acuminate (2).
3. Nares, ventral margin: sharply defined as a crista (1); softened and lost evidently due to growth of maxillpalatines and maxilla at same level of margin in anterolateral aspect (2).
4. Nares, internal bony septum ankylosed to ventral face of dorsal bar along midline: absent (0); present (1). Musser and Clarke [83], character 14.
5. Nares, maxillary process, caudal margin, thickness of dorsal limit of maxillary process of nasals compared to thickness of ventral limit: subequal in rostrocaudal width, narial bar often thin and rod like (1); wider at dorsal end (2); wider at ventral end (3). Musser and Clarke [83], character 16.
6. Nasals, maxillary process, ventral terminus, lack of fusion to ventral rostrum: present (1); lost, fused (2). Musser and Clarke [83], character 17.
7. Nasals, maxillary processes: rostrocaudally flattened (1); mediolaterally flattened (2); both, narial bars mediolaterally flattened rostrally and rostrocaudally flattened caudally (3). Not comparable where nasals are extremely small and slit-like, i.e. in *Diatryma* or *Pelagornis*. Musser and Clarke [83], character 18.
8. Zona flexoria craniofacialis, depth: slight to moderate (1); deep, concave and fissure-like (2). Musser and Clarke [83], character 19.
9. Zona flexoria craniofacialis, mediolateral alignment of caudal margin of processus frontalis of premaxilla and caudal margin of premaxillary (and maxillary, if separated as in schizorhiny)

processes of nasal bone: absent, processus frontalis rostral to premaxillary processes of nasal bone (1); present, caudal termini of processes aligned mediolaterally (2); lost, caudal margin of processus frontalis of nasal bone extends further caudally than those of premaxillary processes of nasal bone (3). Not comparable when frontonasal suture indiscernible or where caudal termini of sutures are obscured by frontal bone. Musser and Clarke [83], character 20; Musser and Clarke [77], character 14; Livezey and Zusi [88], character 603.

1. Zona flexoria craniofacialis: zona absent or indistinct, no transverse sulcus, overlap of processus frontalis of premaxilla and frontals and os nasales continuous over zona (1); well developed zona present marked by transverse sulcus, nasals and processus frontalis cross zona

(2). Mayr and Clarke [81], character 5; Worthy et al. [27], character 8; Livezey [13], character

35.

1. Nasals and/or frontal, (typically) subtriangular depression just caudal to caudal limit of processus frontalis of premaxilla, status (regardless of furrowing of interorbital area): present (1); lost (2). Musser and Clarke [83], character 21.
2. Nasals and/or frontal, rostrally protruding so that processus frontalis of premaxilla (and sometimes premaxillary processes of nasal bone) is obscured: no (1); yes, small, 1-2 rounded rostral bulb(s) protruding dorsal to proccessus frontalis of nasal bone (2); yes, mediolaterally wide, rostrally protruding lamina (3); yes, extremely dorsally inflated frontal bone protrudes dorsal to proccessus frontalis of nasal bone (4). Musser and Clarke [83], character 27.
3. Rostrum, dorsal face, midline length from the apex of the premaxilla to the zona flexoria craniofacialis relative to that of the cranium: rostrum is less than cranial length (1); subequal (2); rostrum longer than cranium (3). Worthy et al. [27], character 1.
4. Antorbital angle, angle created by ankylosis of maxillary process of nasal and maxilla: large, essentially 90 degrees (1); small, approximately 45 degrees (2). Musser and Clarke [83],

character 27.

1. Mesethmoid, osseous interorbital septum, rostral extension: restricted to posterior or another just surpassing premaxillae/frontal contact in rostral extent does not surpass posterior edge of external nares in rostral extent (1); extending rostral to posterior extent of frontal processes of premaxillae and rostral to posterior edge of external nares (2). Clarke and Norell [136], character 26.
2. Orbital margin, salt gland sulcus: absent or non-distinct (0); present (1). Musser and Clarke [83], character 30; Musser and Clarke [77], character 17; Mayr and Clarke [81], character 25;

Livezey [129], character 72.

1. Orbital margin, salt gland sulcus (if present), depth: shallow (1); deep, typically mediolaterally expansive (2). Noncomparable where absent. Musser and Clarke [83], character 31.
2. Interorbital area, midline: convex and not furrowed (1); furrowed along midline, concave (2). Musser and Clarke [83], character 32; Musser and Clarke [77], character 18; Livezey [129],

character 70.

1. Cranium (postorbital braincase), general form: craniocaudally elongate (1); craniocaudally compressed (2). Musser and Clarke [83], character 34; Musser and Clarke [77], character 16;

Livezey and Zusi [88], character 7.

1. Cranium (postorbital braincase), dorsal aspect, concavity or convexity along parietal to occipital: neither is present (1); concave, furrowed (2); convex, with a cristae present along midline (3).
2. Frontoparietal suture, status in adults: present (1); lost (2). Musser and Clarke [83], character 35; Musser and Clarke [77], character 20; Livezey and Zusi [88], character 213; Mayr and

Clarke [81], character 32; Clarke and Norell [136], character 51; Worthy et al. [27], character 15.

1. Maxilla, tomial crest, tomial angle, extension of caudal terminal end as distinct tubercle or short process caudal to jugomaxillary suture and lateral to, and variably free from, arc of jugum: absent (0); present (1). Musser and Clarke [83], character 36; Livezey and Zusi [88], character

408; Livezey [13], character 37.

1. Maxilla, tomial crest, tomial angle, extension of caudal terminal end as distinct tubercle or short process caudal to jugomaxillary suture and lateral to, and variably free from, arc of jugum (if present), shape: triangular (1); subrectangular (2). Noncomparable where absent. Musser and Clarke [83], character 37; Musser and Clarke [77], character 21; Livezey and Zusi [88], character

408.

1. Maxilla, tomial crest, tomial angle, extension of caudal terminal end as distinct tubercle or short process caudal to jugomaxillary suture and lateral to, and variably free from, arc of jugum (if present), length: truncate (1); elongate (2). Noncomparable where absent.
2. Maxillopalatine processes, fused along midline: yes (1); no (2). Musser and Clarke [83], character 38; Mayr and Clarke [81], character 14; Worthy et al. [27], character 7; Livezey [13],

character 38.

1. Maxillopalatine processes, fused to caudal margin of nasal bar so that most of maxillopalatine process visible in antorbital fenestra: no (1); yes (2).
2. Maxillopalatine processes, lateral portion if fused to nasal bar, in line with lateral margin of nasal bar so that maxillopalatine process appears to be a caudal extension of nasal bar: no (1); yes (2). Noncomparable where not fused to caudolateral portion of nasal bar. Worthy et al. [27], character 33.
3. Maxillopalatine processes, craniocaudal elongation: present, extreme (1); lost (2); present, moderate (3). Musser and Clarke [83], character 41.
4. Maxillopalatine processes, ventral margin, dorsoventral location relative to palatines: ventral or subequal to palatines (1); dorsal to palatines (2). Musser and Clarke [83], character 42.
5. Maxillopalatine processes, extensive ankylosis to and/or contact with palatines: present (1); lost (2). Musser and Clarke [83], character 43.
6. Maxillopalatine processes, enclosure of lateral (when mediolaterally flattened) or dorsal (when dorsoventrally flattened) face so that inflated, cone-like structure is created: not enclosed

(1); partially enclosed (2); completely enclosed (3). Musser and Clarke [83], character 44;

Musser and Clarke [77], character 22; Livezey and Zusi [88], character 420; Ericson [14],

character 13.

1. Orbital margin, crista-like orbital margin or supraorbital crest (see Musser and Cracraft 2019): absent, orbital margins not laterally extended and are mostly or completely rounded (0); present (1). Musser and Clarke [83], character 49; Musser and Clarke [77], character 27.
2. Orbital margin, large pores: absent (0); present (1). Musser and Clarke [83], character 52;

Musser and Clarke [77], character 23.

1. Postorbital process, length: truncate (1); extremely elongate, subequal in length to height of quadrate (2). Musser and Clarke [83], character 54; Musser and Clarke [77], character 26; Mayr

and Weidig [52], character 2; Ericson [14], character 2.

1. Postorbital process, orientation of apex: facing rostroventrally (1); facing ventrally (2). Musser and Clarke [83], character 55; Mayr and Weidig [52], character 2.
2. Postorbital process, concavity: convex (1); concave along caudal length of process (2). Musser and Clarke [83], character 56; Musser and Clarke [77], character 27.
3. Postorbital process, dorsal aspect, extends lateral to cranium: no (1); yes (2).
4. Interorbital septum, mediolateral thickness: thin, in some places translucent (1); thick, opaque throughout (2). Musser and Clarke [83], character 57; Musser and Clarke [77], character 28.
5. Lacrimofrontal suture: lost or indistinguishable (1); present (2). Musser and Clarke [83],

character 58; Ericson [14], character 5; Livezey [13], character 15.

1. Lacrimals, site of ankylosis to nasals relative to zona flexoria craniofacialis (ZFC): at same level of ZFC (1); caudal to ZFC (2); rostral to ZFC (3). Musser and Clarke [83], character 61.
2. Lacrimal, ankylosed to jugal or touching jugal bar: absent (0); present (1). Musser and Clarke [83], character 62; Musser and Clarke [77], character 30; Livezey and Zusi [88], character 195;

Mayr and Clarke [81], character 12.

1. Lacrimal, supraorbital process, length: truncate (1); elongate (2). Musser and Clarke [83],

character 64; Livezey and Zusi [88], character 206.

1. Lacrimal, rostral margin, retractor notch: absent (0); present (1). Noncomparable where lacrimal absent [130]. Cracraft [138].
2. Lacrimal, especially supraorbital process, inflated: no (1); yes (2). Musser and Clarke [83], character 65.
3. Lacrimal, supraorbital process, general mediolateral width: narrow (1); wide (2). Musser and Clarke [83], character 66.
4. Lacrimal, supraorbital process, gap present between interorbital area and caudal terminus of supraorbital process: no (1); yes (2). Musser and Clarke [83], character 67; Musser and Clarke [77], character 31; Worthy et al. [27], character 13; Livezey and Zusi [88], character 206.
5. Lacrimal, rostral terminus of head of lacrimal, shape of apex: rounded or subrectangular (1); acuminate (2). Musser and Clarke [83], character 70.
6. Lacrimal, rostral terminus of head of lacrimal, ankylosed to nasals: yes (1); no (2). Musser and Clarke [83], character 77.
7. Lacrimal, rostral terminus of head of lacrimal, extends rostrally with terminus medial to nasals: no (1); yes (2). Musser and Clarke [83], character 73.
8. Lacrimal, additional rostral process (if present), shape: rounded (1); acuminate (2); subrectangular (3). Noncomparable where absent or where incomplete ossification present, and noncomparable where region fused to bill or mesethmoid or jugal. Musser and Clarke [83], character 75.
9. Lacrimal, body/orbital process, foramen: absent (0); present (1). Musser and Clarke [77],

character 32; Livezey and Zusi [88], character 197. Musser and Clarke [83], character 83.

1. Lacrimal, descending process: wide and robust (1); narrow (2). Musser and Clarke [83], character 78; Musser and Clarke [77], character 33; Livezey [13], character 14.
2. Lacrimal, descending process, convexity: concave (1); convex (2). Musser and Clarke [83],

character 138.

1. Lacrimal, descending process: elongate (1); truncate (2). Musser and Clarke [83], character

81; Worthy et al. [27], character 12; Livezey [13], character 14.

1. Lacrimal, ventral apex, shape: rounded or subrectangular (1); acuminate (2). Musser and Clarke [83], character 88.
2. Ectethmoid, pneumatic foramen (exclusive of foramen orbitonasale laterale): absent (0); present, one (1); present, 2 or more (2). Noncomparable in absence of ectehmoid. Musser and Clarke [83], character 84; Musser and Clarke [77], character 37.
3. Ectethmoid, size: absent (0); well developed (1); poorly developed (2). Musser and Clarke [83], character 85; Musser and Clarke [77], character 38; Livezey and Zusi [88], character 189;

Mayr [52], character 1; Mayr and Clarke [81], character 14.

1. Ectethmoid, ankylosed to lacrimal: present (1); lost (2). Noncomparable where absent. Musser and Clarke [83], character 86; Musser and Clarke [77], character 44.
2. Ectehmoid, foramen orbitonasalis medialis: v-shaped (1); small, circular (2); elongate, ovoid

(3); lost (4). Nonomparable when absent due to lack of ankylosis of ectehmoid to ventral portion of antorbital area. Musser and Clarke [83], character 87.

1. Olfactory sulcus, status and depth: shallow (1); deep (2). Musser and Clarke [83], character 88.
2. Fonticulus interorbitalis, status: present (1); lost, only optic foramen present (2). Musser and Clarke [83], character 89.
3. Fonticulus orbitocranialis, caudal extent: limited or essentially absent (1); extensive, continues along coronal plane of squamosal region (2). Musser and Clarke [83], character 91.
4. Jugal, slight medial bowing (concavity): absent (0); present (1). Musser and Clarke [83], character 97.
5. Jugal, maxillojugal articulation, triangular projection on dorsal margin that points dorsally: absent (0); present (1).
6. Jugal, maxillojugal articulation, rostrocaudal location: within rostral 1/3 of jugal (1); just rostral to rostrocaudal midpoint of jugal (2).
7. Jugal, maxillary portion, ovoid projection along rostral portion of ventral margin that may contact maxilla: absent (0); present (1).
8. Vomer contacts premaxilla: absent (0); present (1). Clarke and Norell [136], character 17.
9. Vomers, mediolaterally wide: yes (1); no (2). Musser and Clarke [83], character 98; Worthy

et al. [27], character 39; Mayr and Clarke [81], character 20.

1. Vomers, forming a midline, narrow, and dorsoventrally high lamella: yes (1); no (2). Musser and Clarke [83], character 99; Worthy et al. [27], character 40; Mayr and Clarke [81], character

21.

1. Vomers, caudal ends not fused, more or less deeply cleft: no (1); yes (2). Musser and Clarke [83], character 130; Worthy et al. [27], character 37; Mayr and Clarke [81], character 19.
2. Extensive articulation between the vomer and pterygoid: no (1); yes (2). Clarke and Norell [136], character 14; Livezey [13], character 43.
3. Palatines, contact with premaxilla or maxilla: palatines have contact with maxillae only (1); palatines have contact with premaxillae (2). Musser and Clarke [83], character 131; Musser and Clarke [77], character 45; Clarke and Norell [136], character 16; Cracraft and Clarke [85],

character 8.

1. Palatines, long and thin, especially anteriorly, poorly developed posteriorly, and widely separated anteriorly: absent (0); present (1). Musser and Clarke [83], character 132; Cracraft and Clarke [85], character 36; Ericson [14], character 9.
2. Palatines, contact each other along midline along ventromedial margin: yes (1); no (2). Musser and Clarke [83], character 133; Worthy et al. [27], character 36; Mayr and Clarke [81],

character 17.

1. Palatines, ventral height of medial and lateral crests relative to each other: height of lateral crest greater than that of medial crest (1); heights subequal or crests barely visible (2); height of medial crest is greater than that of lateral crest (3). Musser and Clarke [83], character 134; Musser and Clarke [77], character 52.
2. Palatines, inflated: no (1); yes (2). Musser and Clarke [83], character 135.
3. Palatines, lateral portion: well developed (1); rudimentary or vestigial (2). Musser and Clarke [83], character 136; Musser and Clarke [77], character 47; Worthy et al. [27], character 34;

Livezey and Zusi [88], character 447; Mayr and Clarke [81], character 16; Livezey [13],

character 41.

1. Palatines, ventrally raised oblique crest that extends from lateral to medial margins of ventral face: absent (0); present (1). Musser and Clarke [83], character 138; Musser and Clarke [77],

character 50; Livezey and Zusi [88], character 457.

1. Palatines, caudomedial angle, pneumatic foramen: absent (0); present (1). Noncomparable where absent. Musser and Clarke [83], character 143; Musser and Clarke [77], character 51.
2. Palatines, choanalis, ventral lamella, medial separation of bilateral lamellae: moderate (1); great (2). Musser and Clarke [83], character 141; Musser and Clarke [77], character 53; Livezey

and Zusi [88], character 444.

1. Palatines, choanalis, ventral lamella, rostrocaudal position of caudomedial angle relative to that of caudolateral angle: coincident (1); rostral (2); caudal (3). Not comparable where caudolateral angle and/or caudomedial angle absent. Musser and Clarke [83], character 142; Musser and Clarke [77], character 55; Livezey and Zusi [88], character 443.
2. Palatines, caudolateral angle: absent (0); present (1). Musser and Clarke [83], character 143.
3. Palatines, caudolateral angle, position relative to area of ankylosis with pterygoids: rostral

(1); subequal (2); caudal (3). Not comparable where caudolateral angle absent. Musser and Clarke [83], character 144.

1. Palatines, caudolateral angle, shape: rounded (1); acuminate (2). Not comparable where caudolateral angle absent. Musser and Clarke [83], character 145.
2. Palatines, pterygoid process, pneumatic foramen: present (1); lost (2). Not comparable in absence of pterygoid process. Musser and Clarke [83], character 146; Musser and Clarke [77], character 57.
3. Pterygopalatine juncture, form: syndesmosis and pterygo-palatina propria, extensive rostrocaudally, caudal terminus approaching processus quadraticus pterygoidei (1); articulatio mesipterygo-palatina, with rudimentary gomphosis intrapterygoidea (2); articulatio pterygo- palatina simplex (3); articulatio mesipterygo-palatina, with complete gomphosis intrapterygoidea
4. Musser and Clarke [83], character 147; Musser and Clarke [77], character 58; Worthy et al.

[27], character 43; Livezey and Zusi [88], character 601; Livezey [13], characters 44 and 45.

1. Palatines and pterygoids: sutured (1); segmented (articulated) (2). Musser and Clarke [83],

character 148; Musser and Clarke [77], character 59; Worthy et al. [27], character 41; Mayr and

Clarke [81], character 22; Clarke and Norell [136], character 15; Cracraft and Clarke [85],

character 6.

1. Pterygoid, facies articularis palatina, dorsoventral site of pterygopalatine juncture relative to parasphenoid rostrum (regardless of articulation via basipterygoid processes): slightly ventral, articulatio pterygo-rostroparasphenoidalis absent (1); on rostrum, articulatio pterygo- rostroparasphenoidalis present (2); markedly ventral, articulation of pterygo- rostroparasphenoidalis absent (3). Musser and Clarke [83], character 149; Musser and Clarke [77], character 61; Livezey and Zusi [88], character 4138; Ericson [14], character 8.
2. Pterygoid, rostral end markedly widened: absent (0); present (1). Noncomparable for Paleognathae. Musser and Clarke [83], character 120; Bertelli et al. [139], character 9; Mayr (2008).
3. Pterygoid, regardless of rostral widening, extreme cranial elongtaion of lateral process at pterygo-palatine articulation: no (1); yes (2). Noncomparable where fused to Palatines, eg. Palaeognathae.
4. Pterygoid, regardless of rostral widening, extreme cranial elongtaion of medial process at pterygo-palatine articulation: no (1); yes (2). Noncomparable where fused to Palatines, eg. Palaeognathae.
5. Pterygoid, dorsomedial margin, craniocaudally elongate crista that is well projected dorsally and/or medially, may or may not have a facet for articulation with basipterygoid processes: absent (0); present (1). Musser and Clarke [83], character 121.
6. Pterygoid, dorsomedial margin, craniocaudally elongate crista that projects dorsally and/or medially (if present), rostrocaudal length: truncate, typically the length of roughly 1/4-1/2 of corpus (1); elongate, makes up entire length of pterygoid (2). Noncomparable where crista absent. Musser and Clarke [83], character 122.
7. Pterygoid, dorsolateral margin, concavity: lost or slight (1); deep (2). Musser and Clarke [83], character 123.
8. Pterygoid, ventromedial concavity: lost or slight (1); deep (2). Musser and Clarke [83], character 127.
9. Pterygoid, area medial to area of articulation with quadrate, additional craniocaudally elongate crista projecting dorsally, present along dorsal margin: absent (0); present (1).
10. Pterygoid, basipterygoid processes, continuous with medial crista or lamina: no, projects well medially beyond crista (1); yes (2). Noncomparable where absent. Clarke and Norell [136], character 23.
11. Basisphenoid, basipterygoid processes, rostrocaudal location: well rostral to hypoglossal canals (typically well rostral to basioccipital) (1); just rostral to hypoglossal canals (typically within margin of basioccipital) (2). Noncomparable where absent. Musser and Clarke [83], character 128; Worthy et al. [27], characters 28 and 44; Clarke and Norell [136], character 21;

Cracraft and Clarke [85], character 33.

1. Basisphenoid, basipterygoid processes, orientation: project ventrally (1); project laterally

(2). Noncomparable where absent. Clarke and Norell [136], character 22.

1. Basisphenoid, basipterygoid processes, mediolateral proximity of pair: widely separated (1); coincident, sometimes in contact long midline (2). Noncomparable where absent.
2. Basisphenoid, basipterygoid processes, mediolateral length: elongate (1); truncate (2). Noncomparable where absent. Musser and Clarke [83], character 130; Worthy et al. [27], character 29; Mayr and Clarke [81], character 27; Clarke and Norell [136], character 19.
3. Basisphenoid, basipterygoid processes, craniocaudal length of facet: craniocaudally truncate

(1); craniocaudally elongate (2). Noncomparable where absent.

1. Basiparasphenoid plate is inflated, rounded and broad: absent (0); present (1). Musser and Clarke [83], character 132; Mayr and Clarke [81], character 26; Cracraft and Clarke [85].
2. Alaparasphenoidalis is inflated by the rostral tympanic diverticulum: absent (0); present (1). Cracraft and Clarke [85], charater 13.
3. Cranium, ventral view, location of external aperture of carotid canal: posterior to paroccipital notch (1); in paroccipital notch, including if within fossa parabasalis (2); anterior to paroccipital notch (3). Worthy et al [27], character 32.
4. Quadratopterygoid juncture: articulatio complex, involving both broad contact on facies medialis of processus orbitalis supplementary to condylus pterygoideus quadraticum (0); articulatio duplex, moderate dorsal extension on medial face of orbital process combined with condylus pterygoideus (1); articulatio simplex, retaining vestigial contact on basis of processus orbitalis in addition to primary articulatio with condylus pterygoideus (2); articulatio simplex, virtually limited to condylus pterygoideus (3). Musser and Clarke [83], character 134; Musser and Clarke [77], character 65; Livezey and Zusi [88], character 600; Mayr and Clarke [81],

character 26; Clarke and Norell [136], character 32.

1. Temporal fossa and/or sometimes segmented fossa caudal to crista m. depressor mandibulae, extent: mostly only visible from lateral view, terminal end located ventrocranially to nuchal crest (1); edges of temporal fossae almost meet or do meet above nuchal crest (2). Musser and Clarke [83], character 135.
2. Temporal fossa, depth: shallow (1); deep (2).
3. Temporal fossa, crest: shallow, at same level of top of skull (1); consists of a distinctive crest that is raised prominently (2). Musser and Clarke [83], character 136; Musser and Clarke [77], character 67.
4. Crista m. depressor mandibulae or nearby crista, continued dorsally so that it divides temporal fossa: no (1); yes (2).
5. Zygomatic process, status: present (1); lost (2). Musser and Clarke [83], character 137;

Worthy et al. [27], character 17; Mayr and Clarke [81], character 33.

1. Zygomatic process, if present, dorsoventral location relative to head of quadrate: immediately dorsal to head of quadrate (1); well dorsal to head of quadrate (2).
2. Zygomatic process, length: truncate (1); elongate, reaches level of base of orbital process of quadrate (2). Noncomparable where absent. Musser and Clarke [83], character 138; Musser and Clarke [77], character 68; Livezey and Zusi [88], character 152; Clarke and Norell [136],

character 30.

1. Zygomatic process, ankylosed to postorbital process: no (1); yes (2). Musser and Clarke [83], character 139. Noncomparable where absent.
2. Zygomatic process, articular notch for lateral head of quadrate: notch facing cranially (1); notch angled ventrally (2). Noncomparable in absence of the zygomatic process. Musser and Clarke [83], character 142; Musser and Clarke [77], character 70.
3. Zygomatic process, ossifications (aponeurosis): absent (0); present (1). Noncomparable where absent. Musser and Clarke [83], character 143; Worthy et al. [27], character 18; Mayr and Weidig [52], character 2.
4. Processus suprameaticus: absent as distinct processus, in most or all cases the homologous bone evidently continuous as rostral margin of meatus acusticus externus (0); present as variably prominent postorbital process (1). Musser and Clarke [83], character 145; Musser and Clarke [77], character 77; Livezey and Zusi [88], character 143.
5. Processus suprameaticus (if present), shape: subrectangular (1); acuminate (2). Noncomparable where absent. Musser and Clarke [83], character 152.
6. Occipital, foramen dorsomediana, cf. foramen (ostium): absent, foramina bilaterally symmetrical within occipital region (0); present, distinctly dorsal, often proximate to transverse nuchal crest (1). Musser and Clarke [83], character 147; Musser and Clarke [77], character 72;

Livezey and Zusi [88], character 83.

1. Occipital, transverse nuchal crest, dorsal portion: shallow or barely visible (1); distinct and prominent, raised dorsally (2). Musser and Clarke [83], character 148; Musser and Clarke [77], character 73.
2. Occipital, fonticuli occipitalis: absent (0); present, large, ovoid and perforate (1). Musser and Clarke [83], character 149; Musser and Clarke [77], character 74; Worthy et al. [27],

character 30; Mayr and Clarke [81], character 27; Livezey [16], character 9; Ericson [14],

character 1; Livezey [13], character 5.

1. Occipital, foramen v. occipitalis externae: present or very distinctly visible (1); absent or smoothed to the point of being hardly or not visible (2). Musser and Clarke [83], character 150; Musser and Clarke [77], character 76.
2. Occipital, supraoccipital eminence: right and left portions of occipital complex indistinguishable or have very faint, smoothed division (1); occipital complex distinctly separated by median nuchal crest, appearance of a line or extremely bulbous process dividing the complex (2). Musser and Clarke [83], character 151; Musser and Clarke [77], character 83.
3. Occipital, foramen n. abducentis: round (1); craniocaudally elongate (2). Musser and Clarke [83], character 153; Musser and Clarke [77], character 85.
4. Occipital, occipital condyle, form: essentially circular (1); distinctly bilobate or reniform, lobes partitioned by medial condylar notch, lateromedially elongate (2); essentially round but flattened along ventral margin of foramen magnum (3). Musser and Clarke [83], character 154; Musser and Clarke [77], character 138; Livezey and Zusi [88], character 21; Livezey [13],

character 1.

1. Occipital, occipital condyle, rostrocaudal position relative to exoccipital, processus paroccipitalis: rostral (1); approximately equal or caudal (2). Musser and Clarke [83], character 155; Musser and Clarke [77], character 81; Livezey and Zusi [88], character 27.
2. Occipital, subcondylar fossa: deep (1); shallow (2); lost (3). Musser and Clarke [83],

character 156; Musser and Clarke [77], character 88.

1. Occipital, hypoglossal nerve foramen: circular (1); elongate (2). Musser and Clarke [83],

character 157; Musser and Clarke [77], character 83.

1. Occipital, dorsal apex of nuchal crest, dorsoventral position: subequal or ventral to dorsal base of postorbital process (1); dorsal to postorbital process (2). Musser and Clarke [83], character 159.
2. Occipital, basioccipital and basiparasphenoid: medially compressed (1); laterally splayed
3. Musser and Clarke [83], character 160; Musser and Clarke [77], character 86.
4. Occipital, fossa parabasalis: absent (0); present (1). Musser and Clarke [83], character 161;

Musser and Clarke [77], character 87; Livezey and Zusi [88], character 120.

1. Cranium, proc. paroccipitalis, strongly protruding caudally or caudoventrally, caudally convex: no (1); yes (2). Worthy et al. [27], character 45; Livezey [13], character 3.
2. Occipital, basioccipital, crista basilaris transversa: absent or barely visible (0); present (1). Musser and Clarke [83], character 163; Livezey [13], character 6.
3. Occipital, basioccipital, crista basilaris transversa or homologous site, deeply cleft: no (1); yes (2).
4. Occipital, basiparasphenoid, pneumatic foramen in center: absent (0); present (1). Musser and Clarke [83], character 164; Musser and Clarke [77], character 92.
5. Occipital, basioccipital/basiparasphenoid, concave: no, convex (1); yes (2). Musser and Clarke [83], character 165.
6. Occipital, basioccipital (parasphenoid), ventrally prominent crista running craniocaudally along midline (regardless of concavity): absent (0); present (1).
7. Parasphenoid, medial process, alignment: lateral (1); ventral (2).
8. Parasphenoid, lateral process, alignment: lateral (1); ventral (2).
9. Eustacian tubes, direction of opening: open laterally (1); open at midline (2). Clarke and Norell [136], character 27; Cracraft and Clarke [85], character 12.
10. Basiparasphenoid, eustacian tubes fossa: deep (1); shallow (2). Noncomparable where basisphenoid covering homologous site. Musser and Clarke [83], character 167; Musser and Clarke [77], character 94.
11. Basisphenoid, pair of foramina on either side of eustacian tubes fossa, mediolateral position: on either side of fossa, contacting or near contacting fossa (1); caudal to and further lateral form eustacian tubes fossa (2).
12. Basisphenoid, pair of foramina on either side of eustacian tubes fossa, shape: small, circular

(1); craniocaudally elongate, ovoid (2).

1. Basisphenoid, crista on either side of eustacian tubes fossa, ventral prominence: shallow (1); prominent (2). Noncomparable where ala sphenoid covering homologous site.
2. Skull with highly pneumatic bone posterior to the articulation with the quadrate: absent (0); present (1). Cracraft and Clarke [85], character 14.
3. Quadrate, otic process, lateral face: convex or flat (1); slightly concave (2); deeply concave
4. Musser and Clarke [83], character 177; Musser and Clarke [77], character 96.
5. Quadrate, otic process, medial head, markedly extended caudally compared to lateral head: no, positions subequal (1); yes (2). Noncomparable where heads not well separated. Musser and Clarke [83], character 172.
6. Quadrate, medial condyle: globose (1); linear (2). Musser and Clarke [83], character 174.
7. Quadrate, lateral capitulum, rostrocaudal position relative to quadratojugal articulation: quadratojugal articulation rostral (1); subequal (2); caudal to lateral head (3). Musser and Clarke [83], character 175.
8. Quadrate, tuberculum subcapitulare (if present), prominence: prominent, tuberculate (1); diminuitive, barely raised scar (2). Noncomparable where absent. Musser and Clarke [83], character 183.
9. Quadrate, tuberculum subcapitulare (if present), pars profunda: separated from squamosal capitulum (1); contiguous with squamosal capitulum (2). Noncomparable where absent.
10. Quadrate, tuberculum subcapitulare (if present), orientation of projection: rostral (1); lateral

(2). Noncomparable where absent.

1. Quadrate, otic process, crista lateralis, prominence: absent or extremely shallow (1); present, prominent crista (2).
2. Quadrate, otic process, crista tympanica, prominence: absent or indiscernible (1); present, shallow (2); present, extremely prominent crista (3). Musser and Clarke [83], character 185.
3. Quadrate, otic process, crista tympanica, ventral apex, dorsoventral location: within dorsal half of quadrate or at midpoint (1); within ventral half of quadrate (2). Not comparable where crista absent. Musser and Clarke [83], character 1438.
4. Quadrate, development of intercotylar incisure between prootic and squamosal cotylae: absent (0); present (1). Clarke and Norell [136], character 36; Cracraft and Clarke [85], character

18.

1. Quadrate, otic process, cotylae, degree of separation: cotylae confluent (1); cotylae completely separated (2). Musser and Clarke [83], character 188; Musser and Clarke [77],

characters 97-98; Worthy et al. [27], character 49; Livezey and Zusi [88], characters 150 and

551; Mayr and Clarke [81], character 34; Ericson [14], character 16.

1. Quadrate, otic process, cotyla, relative size: medial head significantly smaller than lateral hed, almost absent (1); subequal (2); medial head larger (3).
2. Quadrate, otic process, caudal face dorsal to crista tympanica (or homologous site), deeply concave: no (1); yes (2). Musser and Clarke [83], character 183.
3. Quadrate, cluster of pneumatic foramina on posterior surface of the tip of dorsal process: absent (0); present (1). Clarke and Norell [136], character 39.
4. Quadrate, otic process, capitulum, medial face, pneumatic foramen (if present), location with respect to crista medialis and crista tympanica: rostral to crista medialis and crista tympanicum (1); rostral to crista tympanicum but caudal to crista medialis or homologous site

(2). Noncomparable where foramen absent. Musser and Clarke [83], character 186.

1. Quadrate, otic process, capitulum, medial face, pneumatic foramen, dorsoventral location: present within dorsal 2/3 (1); present within ventral third (2). Noncomparable where foramen absent. Musser and Clarke [83], character 187.
2. Quadrate, otic process, capitulum, medial face, additional large pneumatic foramen: no (1); yes (2).
3. Quadrate, otic process, capitulum, caudal face, pneumatic foramen, status: present (1); lost

(2). Musser and Clarke [83], character 188; Musser and Clarke [77], character 130; Worthy et al.

[27], characters 51 and 55; Livezey and Zusi [88], character 554; Mayr and Clarke [81],

character 36.

1. Quadrate, orbital process, medial face, depressio protractoris, form: flattened or only slightly concave (1); deeply concave (2). Noncomparable where orbital process absent. Musser and Clarke [83], character 190.
2. Quadrate, orbital process, lateral concavity: convex (1); concave (2). Noncomparable where orbital process absent.
3. Quadrate, orbital process, medially bowed: yes (1); no (2). Noncomparable where orbital process absent. Musser and Clarke [83], character 193; Livezey and Zusi [88], character 535.
4. Quadrate, orbital process, crista orbitocaudilaris, prominence: shallow (1); prominent (2). Noncomparable where orbital process absent.
5. Quadrate, orbital process, crista orbitalis, prominence: shallow (1); prominent (2). Noncomparable where orbital process absent. Musser and Clarke [83], character 194.
6. Quadrate, orbital process, shape of terminus: terminus subequal or wider than orbital process (1); exceptionally tapered toward terminus (2). Noncomparable where orbital process absent. Musser and Clarke [83], character 195; Musser and Clarke [77], character 134-135;

Bertelli et al. [139], character 25; Livezey and Zusi [88], character 535 and 538.

1. Quadrate, orbital process, further medial bowing of tip even if rest of process is medially bowed: no (1); yes (2). Noncomparable where orbital process absent.
2. Quadrate, orbital process, rostral tip, shape: subrectangular or rounded (1); acuminate (2). Noncomparable where orbital process absent. Elzanowski and Stidham [49], character 5.
3. Quadrate, medial condyle, orientation: aligned cranio-caudally (1); aligned medio-laterally

(2). Musser and Clarke [83], character 196; Musser and Clarke [77], character 137; Livezey [13],

character 52.

1. Quadrate, mandibular process, facies articulais pterygoidea (ventral face in those taxa having two): facies articularis, with slight anteromedial eminentia on basis (1); condylar,

tubercular, or jugosublinear (2). Musser and Clarke [83], character 198; Musser and Clarke [77], character 139; Worthy et al. [27], character 56; Livezey and Zusi [88], character 523.

1. Quadrate, orbital process, pterygoid articulation: pterygoid articulation does not reach anteriormost tip (1); pterygoid articulation with no extent up orbital process, restricted to quadrate corpus (2). Clarke and Norell [136], character 31.
2. Quadrate, mandibular articulation: bicondylar articulation with mandible (0); tricondylar articulation, with additional caudal condyle (1); functionally bicondylar articulation, with additional third articulation developed as a shelf caudal and dorsal to lateral condyle (2). Clarke and Norell [136], character 37; Livezey [13], character 51.
3. Quadrate, lateral condyle, location relative to articulation with quadratojugal: caudal (1); rostral (2); subequal (3). Musser and Clarke [83], character 201.
4. Quadrate, lateral condyle, oriented lateromedially: absent (0); present (1). Musser and Clarke [83], character 202; Cracraft and Clarke [85], character 38.
5. Quadrate, facies articularis quadratojugalis: fovea and cotyla (1); incisura—concave, troughlike, raised margin lacking entirely or at least in two, geometrically opposing points (2). Musser and Clarke [83], character 207; Musser and Clarke [77], character 144; Livezey and Zusi [88], character 514.
6. Quadrate, lateral margin of processus lateralis dorsal to cotyla quadratojugalis, prominent tubercle present that projects rostrolaterally past cotyla quadratojugalis: absent (0); present (1). Noncomparable where cotyla/fovea absent. Musser and Clarke [83], character 208; Musser and Clarke [77], character 40; Livezey and Zusi [88], character 514; Clarke and Norell [136],

character 34.

1. Quadrate, facies articularis quadratojugalis, cotyla quadratojugalis, exceptional dorsoventral thickening of the rostroventral margin of cotyla: absent (0); present (1). Noncomparable where cotyla/fovea absent. Musser and Clarke [83], character 214.
2. Quadrate, caudal aspect, prominentia submeatica, status: absent (0); present (1). Noncomparable where full caudal condyle present.
3. Quadrate, caudal aspect, processus submeaticus that diverges from complete contact with quadratojugal articulation, status: absent (0); present (1). Noncomparable where full caudal condyle present.

Skull: Mandible

1. Articular surface, having single centrally located ridge oriented anteroposteriorly: absent (0); present (1). Musser and Clarke [83], character 213; Worthy et al. [27], character 60; Mayr

and Clarke [81], character 38; Cracraft and Clarke [85], character 39; Ericson [14], character 18;

Livezey [13], character 26.

1. Articular surface, having full articulation for caudal condyle: present (1); lost (2).
2. Articular surface, posteromedial and lateral walls: absent (0); present (1). Musser and Clarke [83], character 214; Cracraft and Clarke [85], character 39.
3. Symphysis, lamina ventral to foramina neurovascularis, craniocaudal alignment with dorsal lamina of symphysis: subequal, dorsal and ventral lamina craniocaudally aligned (1); ventral lamina extends caudal to dorsal lamina (2). Musser and Clarke [83], character 215; Musser and Clarke [77], character 145; Livezey and Zusi [88], character 654; Clarke and Norell [136],

character 47; Livezey [13], character 16.

1. Symphysis, length as proportion of total length of mandible (not including retroarticular process if present): short, less than 1/5 (1); intermediate, between 1/5 and 1/3 (2); long, between 1/3 and 1/2 (3). Musser and Clarke [83], character 216; Musser and Clarke [77], character 146;

Livezey and Zusi [88], character 676.

1. Symphysis, symphyseal foramen/foramina: single (1); paired (2). Clarke and Norell [136],

character 52.

1. Symphysis, two strong grooves on ventral surface: absent (0); present (1). Mayr and Clarke [81], character 40; Clarke and Norell [136], character 7.
2. Symphysis, dorsal surface essentially flat: no (1); yes (2). Mayr and Clarke [81], character 43; Clarke and Norell [136], character 44.
3. Ramus, deep groove in ventral surface of the anterior portion: absent (0); present (1). Worthy et al. [27], character 62; Ericson [14], character 20.
4. Symphysis, orientation: straight or dorsally oriented (1); apex ventrally recurved (2). Musser and Clarke [83], character 218.
5. Prominent coronoid process: lost or obsolete (0); present (1). Livezey [13], character 18.
6. Ramus, portion rostral to coronoid process, curvature: present, variably but distinctly decurved (1); obsolete, i.e. virtually straight (2). Musser and Clarke [83], character 219; Musser and Clarke [77], character 149; Livezey and Zusi [88], character 673.
7. Ramus, dorsal ramus rostral to coronoid process, mediolateral thickening and dorsoventral flattening: no (1); yes (2). Livezey [13], character 23.
8. Coronoid process, caudal apex, shape: rounded (1); subrectangular (2). Noncomparable where coronoid does not protrude above surangular.
9. Coronoid process, lateral aspect, concave: no (1); yes (2). Noncomparable where coronoid does not protrude above surangular.
10. Coronoid process or homologous site, lateral aspect, prominent crista that runs rostroventally from fenestra rostralis mandibulae: absent or shallow (1); present, prominent (2).
11. Coronoid process, caudal apex, ramus between coronoid process and articular surface for quadrate: well ventral to caudal apex (1); at same dorsal height as caudal apex (2).
12. Coronoid process, caudal apex, dorsoventral location relative to articular surface for quadrate: immediately dorsal to or subequal to articular surface of quadrate (1); well dorsal (2). Noncomparable where coronoid does not protrude above surangular.
13. Coronoid process, rostral apex, height compared to rostral ramus: well dorsal to ramus (1); at same dorsal height as rostral ramus (2). Noncomparable where coronoid does not protrude above surangular.
14. Coronoid process, dorsal height relative to height of dentary/splenial below: subequal or shorter (1); roughly 2x that of dentary/splenial (2). Noncomparable where coronoid does not protrude above surangular.
15. Splenial, anterior extent: splenial stops well posterior to mandibular symphysis (1); extending to mandibular symphysis, though noncontacting (2); extending to proximal tip of mandible, contacting on midline (3). Clarke and Norell [136], character 43.
16. Coronoid process, rostral and caudal apices, relative heights: subequal (1); caudal apex higher (2); rostral apex higher (3). Noncomparable where coronoid does not protrude above surangular.
17. Ramus, portion caudal to coronoid process or homologous site, medial curvature of dorsal margin: present (1); lost (2).
18. Ramus, portion caudal to coronoid process or homlogous site, medial aspect, depth caudal to fenestra rostralis mandibulae: shallowly concave (1); extremely deep and concave (2).
19. Articular, ventral border caudal to fenestra caudalis mandibulae, medial aspect concavity: convex (1); concave (2).
20. Tuberculum m. adductor mandibulae externus, pars articularis, caput externus (see tuberc. m. AME in Livezey and Zusi [88]), laterally extensive: absent (0); present (1). Musser and Clarke [83], character 221.
21. Ventral angle: absent or indistinct (0); present, marked (1). Musser and Clarke [83], character 222, Musser and Clarke [77], character 120; Livezey and Zusi [88], character 681;

Livezey [13], character 17.

1. Fenestra caudalis mandibulae, medial aspect, large circular foramen penetrating caudally: absent (0); present (1).
2. Surangular, dorsoventrally thick: no (1); yes (2). Musser and Clarke [83], character 225.
3. Surangular and dentary, dorsoventrally thicker than articular area/angular: no (1); yes (2). Musser and Clarke [83], character 226.
4. Angulus mandibulae, dorsally prominent: no (1); yes (2). Noncomparable for Anseriformes. Musser and Clarke [83], character 227.
5. Articular, notch between articular area and medial articular process (recessus conicalis of Tambussi et al. [28]): absent (0); present (1).
6. Medial articular process, length: elongate (1); truncate (2). Musser and Clarke [83],

character 236; Worthy et al. [27], character 68; Mayr and Clarke [81], character 45.

1. Medial articular process, foramen on dorsal face: absent (0); present (1). Musser and Clarke [83], character 233; Worthy et al. 2017, character 66; Clarke and Norell [136], character 41.
2. Medial articular process, rostrally flattened cotyle located at terminus: yes (1); no (2). Noncomparable where truncate.
3. Medial articular process, concave along medial margin: no (1); yes (2). Noncomparable where truncate.
4. Medial articular process, concave along lateral margin: no (1); yes (2). Noncomparable where truncate.
5. Medial articular process, dorsal prominence (distinctly exceeding fossa articularis quadratica): no (1); yes (2). Noncomparable where truncate. Livezey [13], character 25.
6. Medial articular process, orientation: medial (1); caudal (2).
7. Condylar area, lateral condyle, mediolaterally compressed: no (1); yes (2). Noncomparable where truncate.
8. Retroarticular process (if present), dorsoventral thickening: absent, processes thin and elongate (1); present, processes dorsoventrally thick and mediolaterally flattened (2). Noncomparable where absent. Musser and Clarke [83], character 230; Livezey and Zusi [88], character 620.
9. Retroarticular process (if present), ventral protrusion of rostral portion of ventral margin of process relative to ventral margin of articular area: significantly ventral (1); slightly ventral (2); in line or rostral (3). Noncomparable where absent.
10. Dentary, caudal development: strongly forked (1); weakly forked posteriorly into dorsal and ventral rami (2). Musser and Clarke [83], character 231; Musser and Clarke [77], character 123;

Cracraft and Clarke [85], character 4.

1. Pseudotooth bony projections: absent (0); present (1).
2. Area of articulation with quadrate, lateral cotyle, orientation of rostral margin compared to

that of caudal margin: oriented laterally (1); oriented medially (2). Musser and Clarke [83], character 232; Musser and Clarke [77], character 127.

1. Area of articulation with quadrate, cotylae fossae articularis, tuberculum intercotylare: variably tuberculate with intervening depressions (1); single, centrally positioned, rostrocaudally oriented jugum (2). Musser and Clarke [83], character 235; Livezey and Zusi [88]; character 698.
2. Area of articulation with quadrate, processus lateralis mandibulae, size: small, diminutive

(1); prominent (2). Note: not to be confused with cotyla lateralis, it is located rostral to this feature. Musser and Clarke [83], character 237.

1. Proc. lat. mand., process continues rostrally past articular surface: no (1); yes (2).
2. If only 2 condylar articulations present and/or single ridge present, lateral articulation, size: rounded, robust (1); craniocaudally compressed to a sliver (2). Noncomparable where articulation for caudal condyle is not a ridge. Livezey [13], character 27.
3. Condylar area, dorsally projecting hook-like accessory projection caudolateral to cotyla caudalis: absent (0); present (1). Musser and Clarke [83], character 238; Musser and Clarke [77], character 129; Bertelli et al. [139], character 26. Note: This character is not to be confused with a true retroarticular process seen in eg. galloanserines and Phoenicopteridae. The difference is that while the hook-like process seen in Neoaves (eg. Gruiformes) is only present caudolateral to the caudal condyle (between area of articulation of caudal and lateral condyles of quadrate), the true retroarticular process is medial to or within the margin of the caudal cotyla or medial to the cotyla lateralis where the articulation for the caudal condyle is absent. The true retroarticular process must incorporate medial lamina of the mandible. Some Sphenisciformes appear to have a true retroarticular process, but that is actually the area of the caudal fossa of the mandible that has been elevated and flattened dorsally.
4. Condylar area, dorsally projecting hook-like accessory projection present caudolateral to cotyla caudalis, if present, form: present, dorsoventrally elongate and hook-like (1); present but truncate and rounded, sometimes present as a pedestal-like projection with a flattened dorsal surface (2). Musser and Clarke [83], character 238; Musser and Clarke [77], character 129;

Bertelli et al. [139], character 26.

1. True retroarticular process: absent (0); present (1). Musser and Clarke [83], character 270;

Worthy et al. [27], character 64; Mayr and Clarke [81], character 44; Ericson [14], character 19;

Livezey [13], character 20.

1. True retroarticular process, if present, medial margin and impr. m. mand., recessus conicalis present: no (1); yes (2). Noncomparable where absent. Musser and Clarke [83], character 271; Worthy et al. [27], character 65; Livezey [13], character 21.
2. True retroarticular process, if present, rostrocaudal length: truncate (1); elongate (2). Noncomparable where absent. Worthy et al. [27], character 64.
3. True retroarticular process, if present, rostroventral margin ventral to ventral margin of articular: no (1); yes (2). Noncomparable where absent.
4. True retroarticular process, if present, exceptionally tapered throughout: no (1); yes (2). Noncomparable where absent.
5. True retroarticular process, if present, recurvature: abrupt, terminus just dorsally at almost a right angle (1); homogenously recurved (2). Noncomparable where absent. Ericson [14], character 19.
6. Area of fossa caudalis/impressio m. depressor mandibulae, ventral apex, rostrocaudal position: apex subequal in rostrocaudal position to caudal margin of area of articulation with quadrate (1); rostral, apex rostral to caudal margin of area of articulation with quadrate (2);

caudal, apex extended caudally so that caudal fossa visible in dorsal aspect (3). Musser and Clarke [83], character 273.

1. Fossa caudalis, status and depth: absent (0); present, shallow (1); present, deep (2). Noncomparable where hollow. Musser and Clarke [83], character 274. Noncomparable where recessus conicalis present.

Vertebrae

1. Number of presacral vertebrae: 20-22 (1); 18-19 (2); 23 or more (3). Musser and Clarke

[83], character 275; Mayr and Clarke [81], character 55. Vertebrae: Cervical Vertebrae

1. Atlas, processus ventralis corporis, location of caudal apex: caudal (1); ventrocaudal (2). Musser and Clarke [83], character 276; Livezey and Zusi [88], character 832.
2. Atlas, fossa condyloidea, shape: a complete, circular facet (1); lateromedially elongate with a subrectangular facet, does not form circle (2). Musser and Clarke [83], character 277.
3. Atlas, foramina transversaria: absent (0); present (1). Worthy et al. [27], character 72; Mayr

and Clarke [81], character 47.

1. Axis, spinous process, terminus caudal to corpus: no (1); yes (2). Musser and Clarke [83], character 278.
2. Axis, ventral process: essentially absent (0); present, represented by variably thick, rounded or subangular crista (1); present, represented by a ventrally elongated, caudally deflected, rounded spina (2); present, represented by a ventrally elongated, bilaterally compressed, craniocaudally restricted lamina (3). Musser and Clarke [83], character 285; Musser and Clarke [77], character 133; Worthy et al. [27], character 75; Livezey and Zusi [88], character 783.
3. Axis, foramen transversarium: absent (0); present (1). Note: related in part to prominence of processus costalis. Musser and Clarke [83], character 251; Musser and Clarke [77], character 135; Worthy et al. [27], character 73; Mayr and Clarke [81], character 49; Livezey [129],

character 134.

1. Axis, lateral lamina of arcus, pneumatic foramen: absent (0); present (1). Musser and Clarke [83], character 252; Musser and Clarke [77], character 136; Mayr and Clarke [81], character 48;

Livezey [129], character 137.

1. Cervical vertebrae, marked heterogeneity of form involving relative elongation of intermediate elements (regardless of length): present (1); lost (2). Musser and Clarke [83], character 253; Musser and Clarke [77], character 137; Livezey [129], character 141.
2. Cervical vertebrae, corpus, extremely craniocaudally elongate and mediolaterally compressed: no (1); yes (2). Musser and Clarke [83], character 254 and 255; Mayr and Clarke

[81], character 54.

1. Cervical vertebrae, section I, arcus interzygopophysialis lateralis: absent on all elements (0); present (1). Musser and Clarke [83], character 256; Musser and Clarke [77], character 138;

Worthy et al. [27], character 76; Livezey and Zusi [88], character 1386; Mayr and Clarke [81],

character 52; Livezey [129], character 1386.

1. Cervical vertebrae, section I, arcus vertebrae, spinous processes, craniocaudal location of apices: close to cranial margin or midpoint of vertebrae (1); reaching caudal margin of vertebrae

(2). Musser and Clarke [83], character 257.

1. Cervical vertebrae, section II, arcus vertebrae, spinous processes, craniocaudal locations of apices: close to cranial margin of vertebrae (1); at midpoint of vertebrae (2). Musser and Clarke [83], character 260.
2. Cervical vertebrae, section II, zygapophysis caudalis, dorsal portion (torus dorsalis): convex

(1); concave (2). Musser and Clarke [83], character 261.

1. Cervical vertebrae, section II, zygapophysis caudalis: craniocaudally elongate (1); craniocaudally truncate (2). Musser and Clarke [83], character 262.
2. Cervical vertebrae, section II, ansa costotransversaria: as craniocaudally elongate as at approximately half of vertebra (1); craniocaudally short so that ansa is as elongate as 1/3 of vertebra or less (2); like state 1 but toward cranial elements can be essentially as craniocaudally elongate as entire corpus (3). Musser and Clarke [83], character 264.
3. Cervical vertebrae, section II, costal processes (if present), ansa costotransversaria, foramen: absent (0); present (1). Noncomparable where absent. Musser and Clarke [83], character 265.

Vertebrae: Thoracic Vertebrae

1. Number of thoracic vertebrae: 6 or less (1); 8-13 (2); 14 or more (3). Clarke and Norell

[136], character 54.

1. Thoracic vertebrae, spinous process with pneumatic fossa between corpus and spinous process: absent (0); present (1). See description of this feature in Clarke [53].
2. Thoracic vertebrae, corpus, lateral face, pneumatic foramen: absent (0); present (1). Musser and Clarke [83], character 266; Musser and Clarke [77], character 143; Livezey and Zusi [88],

character 850; Clarke and Norell [136], character 58; Ericson [14], character 23; Livezey [13],

character 83.

1. Thoracic vertebrae, corpus, bilateral compression manifested by virtually laminar structure of corpus between facies articulares craniali et caudalis: no, corpus cylindrical (1); present (2). Musser and Clarke [83], character 267; Livezey and Zusi [88], character 858; Ericson [14],

character 27.

1. Thoracic vertebrae, dorsal margins of spinous processes, overlapping craniocaudally (excluding those within notarium, if present): no (1); yes (2). Musser and Clarke [83], character 268.
2. Thoracic vertebrae, dorsal surface and spinous processes, extensive aponeurosis ossificans: no (1); yes (2). Musser and Clarke [83], character 269.
3. Thoracic vertebrae, dorsal lamina of arcus, recessus dorsocranialis pneumatici: absent (0); present (1). Musser and Clarke [83], character 270; Musser and Clarke [77], character 145;

Livezey and Zusi [88], character 866.

1. Thoracic vertebrae, dorsal lamina of arcus, fovea interzygopophysialis, pneumatic foramina: absent (0); present (1). Musser and Clarke [83], character 277; Musser and Clarke [77], character

152; Livezey and Zusi [88], character 867.

1. Thoracic vertebrae, transverse processes, craniocaudal gradual but distinct lateral elongation of processes in seratium among presynsacral elements: absent, transverse processes essentially of uniform width throughout the presynsacral elements or moderately reversed grade (0); present, transverse processes distinctly increasing in width throughout the presynsacral elements (1). Musser and Clarke [83], character 272; Musser and Clarke [77], character 147; Livezey and Zusi [88], character 875.
2. Thoracic and sacral vertebrae, caudalmost elements (cranial to synsacral vertebrae), facies articularis caudalis (of penultimate element) and facies articularis cranialis (of ultimate element), type: series completely heterocoelous (1); at least part of series with subround, central articular surfaces (e.g., amphicoelous/opisthocoelous) that lack the dorsoventral compression and saddle- shaped articular surface seen in heterocoelous vertebrae (2). Musser and Clarke [83], character 273; Musser and Clarke [77], character 148; Livezey and Zusi [88], character 888; Mayr and

Clarke [81], character 57; Ericson [14], character 22.

1. Thoracic vertebrae, notarium: present (1); lost (2). Musser and Clarke [83], character 274;

Musser and Clarke [77], character 151; Worthy et al. [27], character 78; Livezey and Zusi [88],

character 892; Mayr and Clarke [81], character 55; Clarke and Norell [136], character 60;

Ericson [14], character 25; Livezey [13], character 56.

1. Thoracic vertebrae, ventral processes, widely bifurcated (lateral termini extending laterally beyond costal facets): no (1); yes (2). Musser and Clarke [83], character 278.
2. Caudalmost presacral vertebrae with deep lateral excavations: no (1); yes (2). Musser and Clarke [83], character 285; Worthy et al. [27], character 77; Mayr and Clarke [81], character 58;

Clarke and Norell [136], character 58; Ericson [14], character 23.

1. Caudalmost praesacral vertebrae deep lateral excavations, if present, form: deep depressions that are not medially deeper than corpus of vertebrae (1); truly pleurocoelous, with clearly defined hole-like appearance (2). Clarke and Norell [136], character 58; Ericson [14], character

23.

Vertebrae: Synsacrum and Caudal Vertebrae

1. Synsacrum (maximally including thoracic vertebrae): 14-19 (1); 13-13 (2). Musser and

Clarke [83], character 2138; Musser and Clarke [77], character 154; Livezey and Zusi [88],

character 897; Mayr and Clarke [81], character 91; Clarke and Norell [136], character 61;

Livezey [13], character 57.

1. Synsacrum, cranialmost vertebrae caudales synsacri, lateral faces of vertebrae, penetration and visibility through foramen ilioischiadicum (lateral perspective): absent (0); present (1). Musser and Clarke [83], character 281; Musser and Clarke [77], character 158; Livezey and Zusi [88], character 933.
2. Synsacrum, mediolateral compression of the corpi of the synsacro-thoracic and synsacro- lumbar vertebrae: not very mediolaterally compressed (1); two or more vertebrae conspicuously mediolaterally compressed (2). Ericson [14], character 28.
3. Free caudal vertebrae, number (including pygostyle): 5-6 (1); 7-8 (2); 9-13 (3). Musser and

Clarke [83], character 288; Clarke and Norell [136], character 63.

1. Pygostyle, fused to last free caudal via spinous process of caudal vertebra(e): no (1); yes

(2). Musser and Clarke [83], character 283.

1. Pygostyle, fused to last free caudal(s) via ventral processes: no (1); yes (2). Musser and Clarke [83], character 284.
2. Pygostyle, foramen in lateral lamina: absent (0); present (1). Musser and Clarke [83], character 285.
3. Free caudal vertebrae, spinous processes, mediolaterally wide bifurcation throughout series, status: present (1); lost (2). Musser and Clarke [83], character 286.
4. Free caudal vertebrae, ventral processes, status: present (1); lost (2). Musser and Clarke [83], character 287; Mayr and Clarke [81], character 59.
5. Free caudal vertebrae, ventral processes (if present), general orientation of apex: cranioventral (1); cranial (2). Musser and Clarke [83], character 288.
6. Free caudal vertebrae, spinous process, form: rounded (1); subrectangular (2). Musser and Clarke [83], character 289.
7. Free caudal vertebrae, transverse processes: truncate (1); elongate (2). Musser and Clarke [83], character 290; Clarke and Norell [136], character 65.
8. Pygostyle, apex, shape: subrectangular, tapered (1); rounded (2); hamate (3); spatulate (4).

Musser and Clarke [83], character 291.

1. Pygostyle, apex, dorsoventral departure from major axis of pygostyle: absent (0); dorsal (1); ventral (2). Noncomparable if spatulate. Musser and Clarke [83], character 292.
2. Pygostyle, craniocaudal length: elongate (1); truncate (2). Musser and Clarke [83], character

293.

1. Spina externa rostri: absent or obsolete (0); present (1). Musser and Clarke [83], character 294; Musser and Clarke [77], character 159; Worthy et al. [27], character 85; Livezey and Zusi

[88], character 1457; Mayr and Clarke [81], character 70; Livezey [13], character 60.

1. Spina externa rostri, length: truncate, shorter than craniolateral processes (1); elongate, longer than craniolateral processes (2). Noncomparable where spina externa absent. Musser and Clarke [83], character 296; Musser and Clarke [77], character 159; Worthy et al. [27], character

85; Livezey and Zusi [88], character 1457; Mayr and Clarke [81], character 70.

1. Spina externa rostri, shape: fan-shaped (1); spine-like (2). Musser and Clarke [83], character 297; Worthy et al. [27], character 85; Mayr and Clarke [81], character 70.
2. Spina interna rostri: present (1); lost (2). Musser and Clarke [83], character 299; Worthy et

al. [27], character 83; Bertelli et al. [139], character 45.

1. Spina interna rostri (if present), pneumatic foramen: absent (0); present (1). Noncomparable where spina interna absent. Musser and Clarke [83], character 301.
2. Spina externa rostri (if present), pneumatic foramen: absent (0); present (1). Noncomparable where spina externa absent. Musser and Clarke [83], character 302.
3. Spina externa rostri (if present), lateral faces deeply concave: no (1); yes (2). Musser and Clarke [83], character 306.
4. Depressiones (sulcus) articulares coracoidei, ventral lip: reaches ventrally (1); reaches dorsally (2). Musser and Clarke [77], character 162. Musser and Clarke [83], character 308.
5. Depressiones (sulcus) articulares coracoidei, crossed: no (1); yes (2). Musser and Clarke [83], character 309; Mayr and Clarke [81], character 69; Clarke and Norell [136], character 75;

Ericson [14], character 34.

1. Processus craniolateralis: extremely long, creates fenestra (1); prominent but more truncate than state 1 (2); extremely truncate, barely projected (3). Musser and Clarke [83], character 313;

Musser and Clarke [77], character 164; Livezey and Zusi [88], character 1441; Livezey [13],

character 62.

1. Processus craniolateralis with respect to major axis of carina, angle: extending laterally (1); extending rostrally (2). Musser and Clarke [83], character 314; Musser and Clarke [77], character 165; Livezey and Zusi [88], character 1442.
2. Processus craniolateralis, apex, shape: trapezoidal (1); rounded (2); cruciate (3). Musser and

Clarke [83], character 312.

1. Processus craniolateralis, apex, cranial extension of lateral margin (sometimes accompanied by cranial extension of dorsal margin and/or caudal extension of lateral margin): no (1); yes (2); yes, extreme (3).
2. Processus craniolateralis, apex, width compared to width of base: subequal (1); apex more narrow than base (2). Musser and Clarke [83], character 313.
3. Ventral face of sternum, base of processi craniolateralis and/or processus craniolateralis proprius, impressio origii m. sternocoracoidei: present (1); lost or extremely shallow (2). Musser and Clarke [77], character 166; Livezey and Zusi [88], character 1449. Musser and Clarke [83],

character 314.

1. Visceral face of sternum, sulcus medianus sterni immediately caudal to cranial margin, pneumatic foramen and/or depression exclusive of pneumatic pores: absent (0); present, undivided, enclosing pneumatic pores and os spongiosum (1); present, divided medially by osseus lamina or trabecula, enclosing pneumatic pores and os spongiosum (2). Musser and Clarke [83], character 315; Musser and Clarke [77], character 167; Livezey and Zusi [88],

character 1438; Mayr and Clarke [81], character 72; Livezey [13], character 66.

1. Visceral face of sternum, sulcus medianus sterni and/or pneumatic foramen: begins within margin of or is immediately caudal to pila coracoidea (1); significantly caudal to pila coracoidea, approximately caudal to base of carina (2). Musser and Clarke [83], character 316; Musser and Clarke [77], character 168; Livezey and Zusi [88], character 1439.
2. Visceral face of sternum, pneumatic pores, exclusive of those included within pneumatic foramen: absent (0); present, at cranial margin (1); present, along medial sulcus (2); present, within both cranial margin and medial sulcus (3). Musser and Clarke [83], character 317; Musser and Clarke [77], character 169; Worthy et al. [27], characters 85 and 138; Livezey and Zusi [88],

character 1413; Livezey [13], character 64.

1. Facies visceralis sterni, sulcus medianus sterni: present (1); lost (2). Livezey and Zusi [88], character 1414.
2. Ventral face, facies muscularis, sulcus ventrolateralis (longitudinal trough on ventral surface of element immediately medial to processus costales): absent or indistinct (1); distinct, typically for length of costal margin (2). Musser and Clarke [83], character 318; Musser and Clarke [77], character 177; Livezey [129], character 153.
3. Costal margin, craniocaudal length relative to that of entire sternum along median axis: less than ¼ (1); between 1/4 and ¾ (2); greater than ¾ (3). Musser and Clarke [83], character 319; Musser and Clarke [77], character 172; Worthy et al. [27], character 86; Livezey and Zusi [88],

character 1414; Livezey [129], character 1414.

1. Costal margin, articular surfaces for sternal ribs: 4 or less (1); 5-7 (2); 8-9 (3). Musser and

Clarke [83], character 320; Musser and Clarke [77], character 173; Worthy et al. [27], character

81; Mayr and Clarke [81], character 77; Clarke and Norell [136], character 76.

1. Costal margin, linea intermuscularis dorsolateralis: absent (0); present (1). Musser and Clarke [83], character 322; Clarke and Norell [136], character 83.
2. Costal margin, linea intermuscularis dorsolateralis, mediolateral location of caudal terminus: medial only to trabecula caudolateralis (if present) (1); medial to trabecula cauolateralis and intermediana (if present) but lateral to mediana (2); medial to lateral margin of trabecula mediana (3). Noncomparable where linea intermuscularis dorsolateralis absent. Musser and Clarke [83], character 323.
3. Costal margin, linea intermuscularis dorsolateralis, caudal extent: within rostral half of sternum only (1); extends to caudal margin of sternum or well within caudal half of sternum (2). Noncomparable where linea intermuscularis dorsolateralis absent. Musser and Clarke [83], character 327.
4. Carina, cranial margin, lateral crest: present (1); lost (2). Noncomparable for taxa without a carina. Musser and Clarke [83], character 325; Musser and Clarke [77], character 176; Livezey and Zusi [88], character 1207.
5. Carina, cranial margin, sulcus carinae: absent (0); present (1). Not comparable for taxa without a carina. Musser and Clarke [83], character 326; Musser and Clarke [77], character 183; Livezey and Zusi [88], character 1213.
6. Carina, sulcus carinae or homologous site, pneumatic foramen: absent (0); present, variable size and shape (1). Not comparable for taxa without a carina. Musser and Clarke [83], character 327; Musser and Clarke [77], character 178; Livezey and Zusi [88], character 1218.
7. Carina, apex, site relative to spina externa rostri or most proximal point of sternum: subequal to caudal location of spina externa rostri or homologous site (1); located caudally (2); located extremely cranially (3). Noncomparable where carina absent. Musser and Clarke [83], character 329; Musser and Clarke [77], character 185; Livezey and Zusi [88], character 1498;

Clarke and Norell [136], character 72; Ericson [14], character 35.

1. Carina, apex, facet for articulation with furcula: absemt (0); present as incisura or cavity (1). Musser and Clarke [77], character 178; Musser and Clarke [83], character 327; Livezey and Zusi 2006), character 1218.
2. Carina, cranial margin, relative widths of dorsal and ventral portions: dorsal margin wider

(1); ventral margin wider (2); width of both portions subequal (3). Noncomparable where carina absent. Musser and Clarke [83], character 332; Musser and Clarke [77], character 181.

1. Carina, maximal depth ventral and normal to body of sternum, facies muscularis, relative to minimal width of body of sternum (exclusive of processes laterales, if present) across points on costal margin directly lateral to that of maximal depth of carina: height is equal to or greater than width of body, not including processus craniolateralis (1); height is less than width of body (2). Noncomparable where carina absent. Musser and Clarke [83], character 333; Musser and Clarke [77], character 188; Livezey and Zusi [88], character 1499; Ericson [14], character 35.
2. Carina, apex, shape: rounded (1); acuminate (2); trapezoidal (3); bifurcated (4). Noncomparable where carina absent. Musser and Clarke [83], character 334.
3. Carina, recurvature of cranial margin: absent (0); present (1). Noncomparable where carina absent. Musser and Clarke [83], character 335.
4. Carina, apex and ventral keel, reinforced with thickened jugum: no (1); yes (2). Noncomparable where carina absent. Musser and Clarke [83], character 336.
5. Carina, linea intermuscularis ventromedialis, status: present (1); lost (2). Noncomparable where carina absent. Musser and Clarke [83], character 337.
6. Carina, caudal margin, continues to caudal margin of trabecula mediana: yes (1); no (2). Noncomparable where carina absent. Musser and Clarke [83], character 339.
7. Sternum, caudal margin: with 4 or more notches/fenestrae (1); with 2 notches/fenestrae (2);

without notches/fenestrae (3); with 3 notches/fenestrae (4). Musser and Clarke [83], character

340; Musser and Clarke [77], character 183; Livezey and Zusi [88], character 1488; Mayr and

Clarke [81], character 73.

1. Incisura and fenestra caudolateralis (if present), cranial extent (length of fenestra measured using body of sternum, not trabecula lateralis): elongate—length of incisura and fenestra greater than 2/3 craniocaudal length of corpus sterni, approaching terminis caudalis of processus costales sterni (1); intermediate—length of incisura and fenestra between 1/3 and 2/3 of craniocaudal length of corpus sterni (2); abbreviate—length of incisura and fenestra less than 1/3 craniocaudal length of corpus sterni (3). Noncomparable where incisura (fenestra) and/or trabecula absent.

Musser and Clarke [83], character 341; Musser and Clarke [77], character 184; Livezey and Zusi [88], character 1483.

1. Processus caudolateralis (if present), orientation relative to body of sternum as reflected (in part) by angle defined by incisura caudolateralis, cranial vertex of angle: angle undefined, processus is parallel to costal margin and "vertex" is ellipsoidal (1); laterally splayed, approximately 45 degrees (2); angle acute, less than 15 degrees (3). Noncomparable where incisura (fenestra) and/or trabecula absent. Musser and Clarke [83], character 342; Musser and Clarke [77], character 185; Livezey and Zusi [88], character 1484.
2. Trabecula lateralis, status: absent (0); present (1). Musser and Clarke [83], character 344.
3. Trabecula caudolateralis (if present), shape of caudal terminal margin: rounded or (sub)rectangular (1); (sub)acuminate (2); cruciate, with transverse pila (3). Noncomparable where presence of trabecula uncertain or known to be absent. Musser and Clarke [83], character 345; Musser and Clarke [77], character 186; Livezey and Zusi [88], character 1485.
4. Trabecula caudolateralis (if present), orientation of caudal terminal margin: caudal, does not deviate from trabecula lateralis axis (1); medial (2); lateral (3). Noncomparable where presence of trabecula uncertain or known to be absent. Musser and Clarke [83], character 352.
5. Trabecula mediana, thickened, raised and bifurcated crista that connects to the ventral margin of the carina and extends to the caudolateral margin of trabecula mediana: absent (0); present (1). Noncomparable where carina absent. Musser and Clarke [83], character 347.
6. Dorsal lip, labrum interna sterni, pair of rostrally oriented projections: absent (0); present
7. Musser and Clarke [83], character 348; Musser and Clarke [77], character 161.
8. Dorsal lip, labrum interna sterni, pair of rostrally oriented projections (if present), mediolateral position: gap present between 2 processes (1); 2 processes contact each other (2). Noncomparable where processes absent. Musser and Clarke [83], character 352.
9. Dorsal lip, labrum interna sterni, pair of rostrally oriented projections (if present), mediolateral width: wide (1); narrow (2). Noncomparable where processes absent. Musser and Clarke [83], character 353.
10. Trabecula mediana, margin and caudal terminus, tapering: untapered or weakly tapered (1); distinctly tapered (2). Musser and Clarke [83], character 355; Livezey and Zusi [88], character 1491.
11. Trabecula mediana, caudal terminus, shape: subrectangular (1); rounded or acuminate (2); cruciate with rounded transverse pila extending laterally beyond mediana (3); cruciate with elongate, acuminate transverse pila extending laterally beyond mediana (4). Musser and Clarke [83], character 356; Musser and Clarke [77], character 189; Livezey and Zusi [88], character

1491.

1. Trabecula mediana or intermediana, fused to trabecula lateralis: no (1); yes (2). Noncomparable where trabeculae absent. Musser and Clarke [83], character 357.
2. Trabecula mediana, mediolateral width: mediolaterally narrow, essentially confined to midline of sternum (1); mediolaterally wide (2). Musser and Clarke [83], character 359.
3. Trabecula caudolateralis and mediana, relative caudal extents: mediana>caudolateralis (1); caudolateralis>mediana (2). Noncomparable if no notches or fenestrae present. Musser and Clarke [83], character 360; Musser and Clarke [77], character 190; Livezey and Zusi [88],

character 1492; Livezey [13], character 63. Ribs

1. Thoracic ribs, medial face, pneumatic foramina between capitulae: present (1); lost (2). Musser and Clarke [83], character 366; Musser and Clarke [77], character 191; Livezey [129],

character 132; Livezey [13], character 59.

1. Thoracic ribs, dorsal portion, craniocaudally thickened: yes (1); no (2). Musser and Clarke [83], character 367.
2. Uncinate processes on thoracic ribs: absent (0); present (1). Musser and Clarke [83], character 368; Mayr and Clarke [81], character 74; Livezey [13], character 58.
3. Uncinate processes on thoracic ribs, fusion to ribs: fused (1); unfused (2). Noncomparable where absent. Musser and Clarke [83], character 369; Clarke and Norell [136], character 69.
4. Uncinate processes on thoracic ribs, shape of terminus: subrectangular (1); rounded (2). Noncomparable where absent. Musser and Clarke [83], character 370.
5. Uncinate processes on thoracic ribs, angle of articulation: oriented caudally (1); oriented dorsocaudally (2). Noncomparable where absent. Musser and Clarke [83], character 377.
6. Uncinate processes on thoracic ribs, length: truncate (1); elongate (2). Noncomparable where absent. Musser and Clarke [83], character 373.

Shoulder girdle: Coracoid

1. Omal end, processus acrocoracoideus: craniocaudally aligned (1); mediolaterally aligned (2).
2. Coracoid, acrocoracoid tip: straight (1); hooked medially (2). Clarke and Norell [136],

character 95.

1. Coracoid, processus acrocoracoideus, orientation relative to facies articularis humeralis in dorsal aspect: acrocoracoid directed primarily cranially, forming wide angle with humeral facet

(1); directed primarily medially forming near right angle with medial margin of humeral facet

1. Worthy et al. [27], character 99.
2. Coracoid, omal end, facies articularis humeralis: concave (1); flat or convex (2). Worthy et al. [27], character 130.
3. Coracoid, omal end, facies articularis humeralis, orientation: dorsoventral (1); oblique, dorsolateroventral (2); horizontal, mediolateral (3). Worthy et al. [27], character 132.
4. Coracoid, recess. infra acrocoracoid, single prominent ridge present along midline, stretching craniocaudally: absent (0); present (1).
5. Coracoid, recess. infra acrocoracoid, large pneumatic foramen: absent (0); present (1). Worthy et al. [27], character 96.
6. Coracoid, insertion of ligamentum acrocoracoprocoracoideum strongly ventromedially protruding: no, essentially coplanar with craniocaudal axis of shaft of coracoid (1); yes (2). Musser and Clarke [83], character 375; Musser and Clarke [77], character 193; Livezey and Zusi [88], character 1267.
7. Coracoid, facies articularis clavicularis overhanging sulcus supracoracoideus or homologous site: no (1); yes (2). Musser and Clarke [83], character 376; Mayr and Clarke [81],

character 64; Clarke and Norell [136], character 95.

1. Coracoid, facies articularis clavicularis, if overhanging sulcus supracoracoideus, (medial aspect): subhorizontal (1); diagonal, with most proximal portion being located along dorsal margin (2). Noncomparable where not overhanging. Musser and Clarke [83], character 378.
2. Coracoid, facies articularis clavicularis, if overhanging sulcus supracoracoideus, insertion of ligamentum acrocoracoprocoracoideum, markedly concave: no (1); yes (2).
3. Coracoid processus acrocoracoideus, impressio ligamenti acrocoracohumeralis: deep (1); shallow (2). Musser and Clarke [83], character 385; Musser and Clarke [77], character 195;

Livezey and Zusi [88], character 1276; Mayr and Clarke [81], character 67.

1. Processus procoracoideus, angle of articulation with shaft of coracoid: at right angle to shaft

(1); at acute angle (2). Noncomparable where processus absent.

1. Processus procoracoideus, medial length: truncate (1); elongate (2). Noncomparable where processus procoracoideus absent. Musser and Clarke [83], character 388; Musser and Clarke [77], character 197; Livezey and Zusi [88], character 1283.
2. Processus procoracoideus, terminus, shape: no spatulation or expansion present (1); spatulation and expansion of terminus present (2); tapering present (3). Noncomparable where processus procoracoideus absent or fused to head of coracoid. Musser and Clarke [83], character 385.
3. Processus procoracoideus, terminus, caudally projecting, acuminate flange: absent (0); present (1). Noncomparable where processus procoracoideus absent or fused to head of coracoid.
4. Processus procoracoideus, tuberculum apicalis procoracoidei, ventral curvature: absent or slight (1); present (2). Noncomparable where processus procoracoideus absent. Musser and Clarke [83], character 387.
5. Processus procoracoideus, extending distally as a craniocaudally elongate, sharp, crest along midline of shaft (crista procoracoidei of Livezey [137]): no (1); yes (2). Noncomparable where processus procoracoideus absent. Musser and Clarke [83], character 388; Livezey and Zusi [88], character 1283.
6. Scapular cotyla, form: shallow (1); deep, cuplike (2). Musser and Clarke [83], character 391; Worthy et al. [27], character 95; Bertelli et al. [139], character 37; Clarke and Norell [136],

character 84; Ericson [14], character 40.

1. Coracoid, scapular cotyla, shape of surrounding labrum: triangular, all three apices acuminate (1); proximal apex acuminate, distal apices rounded (2); all apices rounded (3); two (typically distal) apices acuminate, one (typically proximal) apex rounded (4).
2. Coracoid, scapular cotyla, dorsal prominence of labrum: prominent along all margins (1); only prominent along caudal margin, omal margin grades into shaft (2); no margins prominent

(3); like state 2 except medial margin grades into shaft, lateral omal margin prominence is preserved (4).

1. Scapular cotyla, large pneumatic foramen or foramina: absent (0); present (1). Musser and Clarke [83], character 392.
2. Coracoid, scapular cotyla, angle of primary axis as determined by location of proximal apex versus those of distal apices: skewed laterally, proximal apex lateral to midline (1); central, proximal apex exactly in line between distal apices in accordance with coracoid axis craniocaudally (2); skewed medially (3).
3. Coracoid, scapular cotyla, proximodorsal elevation along caudal margin: none, cotyla flush with scapula (1); present (2).
4. Scapular cotyla, distolateral apex (or apices), lateral extension beyond level of proximal apex: absent or negligible (1); present (2).
5. Coracoid, scapular cotyla, crista above cotyle extending to head, form: mediolaterally wide at base so a triangular space is created (1); mediolaterally compressed so that a true, single crista is present (2).
6. Coracoid shaft, foramen nervi supracoracoidei: absent (0); present (1). Musser and Clarke [83], character 393; Worthy et al. [27], character 93; Livezey and Zusi [88], character 1286;

Mayr and Clarke [81], character 63; Clarke and Norell [136], character 96; Ericson [14],

character 37; Livezey [13], character 69.

1. Processus procoracoideus, pneumatic foramen directly below facies articularis scapularis which does not penetrate shaft: absent (0); present (1). Musser and Clarke [83], character 394; Musser and Clarke [77], character 198; Livezey and Zusi [88], character 1286.
2. Processus procoracoideus, large, ovoid pneumatic foramen directly below facies articularis scapularis which does not penetrate shaft: absent (0); present (1). Note: homology with previous character uncertain. Mayr and Clarke [81], character 66; Ericson [14], character 39.
3. Coracoid shaft, general form sensu length relative to width of facies articularis sternalis (not including additional projections): elongate—length between 3 and 4x width (1); typically proportioned—length between 2 and 3x width (2); truncate (less than 2x width) (3). Musser and Clarke [83], character 395; Musser and Clarke [77], character 199; Livezey and Zusi [88],

character 1292; Clarke and Norell [136], character 86.

1. Coracoid shaft, linea musc. vent., prominent and raised crista craniocaudally: no (1); yes

(2). Clarke and Norell [136], character 93.

1. Coracoid shaft, dorsal surface, lateral angle, crista extending cranially, position: extends to medial portion of shaft proximally just under scapular cotyla (1); lateral, in line with lateral margin of coracoid (2).
2. Sternal coracoid, impressio musculi sternocoracoidei on dorsal surface of extremitas sternalis: shallow (1); deep (2). Musser and Clarke [83], character 396; Musser and Clarke [77],

character 200; Livezey and Zusi [88], character 1294; Mayr and Clarke [81], character 67.

1. Sternal coracoid, impressio musculi sternocoracoidei, sulcus m. sternocoracoideus: absent, area smooth (0); present, defined ridges present (1). Musser and Clarke [83], character 397; Worthy et al. [27], character 135; Ericson [14], character 38.
2. Sternal coracoid, dorsal surface, impressio m. sternocoracoidei, pneumatic foramen, status: absent (0); present (1). Musser and Clarke [83], character 398; Musser and Clarke [77], character

201; Worthy et al. [27], character 134; Bertelli and Chiappe [140], character 29; Mayr and

Clarke [81], character 67; Livezey [13], character 70.

1. Sternal coracoid, crista articularis sternalis, cranial recurvature: absent (0); present (1). Musser and Clarke [83], character 399.
2. Sternal coracoid, crista articularis sternalis, ventral recurvature: absent (0); present (1). Musser and Clarke [83], character 400.
3. Sternal coracoid, medial angle: rounded (1); acuminate (2). Musser and Clarke [83],

character 401; Livezey [129], character 194.

1. Sternal coracoid, medial angle, additional cranially oriented projection along cranial margin: absent (0); present (1). Noncomparable where crista extends along length of coracoid. Musser and Clarke [83], character 402.
2. Sternal coracoid, medial angle, additional cranially oriented projection along cranial margin (if present), shape of apex: rounded (1); acuminate (2). Noncomparable where absent. Musser and Clarke [83], character 403; Musser and Clarke [77], character 202; Livezey [129], character

194.

1. Sternal coracoid, medial angle, additional cranially oriented projection along cranial margin, if present, craniocaudal length: craniocaudally truncate (1); craniocaudally elongate (2). Noncomparable where absent. Musser and Clarke [83], character 402.
2. Sternal coracoid, processus lateralis, length: truncate (1); elongate (2). Musser and Clarke [83], character 405; Clarke and Norell [136], character 92.
3. Sternal coracoid, processus lateralis, shape of terminus: rounded or subrectangular (1); acuminate (2). Musser and Clarke [83], character 406.
4. Sternal coracoid, processus lateralis, recurvature of cranial margin: absent or negligible (0); present (1). Musser and Clarke [83], character 407.
5. Sternal coracoid, processus lateralis, recurvature of caudal margin: absent or negligible (0); present (1). Musser and Clarke [83], character 408.
6. Sternal coracoid, processus lateralis, apex, orientation: lateral (1); rostral (2); caudal (3). Musser and Clarke [83], character 409; Musser and Clarke [77], character 203; Livezey and Zusi [88], character 1303.
7. Sternal coracoid, processus lateralis, apex, craniocaudal location: well cranial to lateral angle (1); just above lateral angle or subequal (2). Musser and Clarke [83], character 413.
8. Sternal coracoid, lateral angle, mediolateral position relative to shaft of coracoid: medial to lateral margin of shaft (1); lateral to lateral margin of shaft (2). Musser and Clarke [83], character 414.
9. Sternal coracoid, ventral aspect, distolateral corpus (just medial to lateral angle), deeply concave: no (1); yes (2). Musser and Clarke [83], character 412.
10. Sternal portion of coracoid, ventral aspect, distomedial corpus (just lateral to medial angle), deeply concave: no (1); yes (2). Musser and Clarke [83], character 413.
11. Sternal coracoid, facies articularis sternalis, labrum externa, general cranial extent of cranial margin (and correlated cranial expanse) relative to those of labrum interna: former approximately equal to latter, producing facies articularis of dorsoventrally equal expanse (1); former distinctly caudal to latter, producing internally (dorsally) angled facies articularis (2); former significantly cranial to the latter, producing externally (ventrally) angled facies articularis (3). Musser and Clarke [83], character 414; Livezey and Zusi [88], character 1314.
12. Sternal coracoid, facies articularis sternalis, labrum externa, labrum along lateral angle ventrocranially angled: no (1); yes (2).
13. Sternal coracoid, facies articularis sternalis, labrum externa, labrum along medial angle ventrocranially angled: no (1); yes (2). Musser and Clarke [83], character 415; Musser and Clarke [77], character 205; Livezey and Zusi [88], character 1314.
14. Sternal coracoid, facies articularis sternalis, notch in labrum: absent (0); present (1). Based on discussion in Mayr et al. [11].

Shoulder girdle: Furcula

1. General shape: laterally splayed, "u-shaped" (1); mediolaterally compressed, "v-shaped"
2. Musser and Clarke [83], character 416; Worthy et al. [27], character 144.
3. Apophysis, status: absent (0); present (1). Musser and Clarke [83], character 417; Mayr and

Clarke [81], character 63; Ericson [14], character 52; Livezey [13], character 67.

1. Apophysis if present, caudal prominence: diminutive, barely raised (1); extremely elongate

(2); intermediate (3). Noncomparable where apophysis absent. Musser and Clarke [83], character 418; Worthy et al. [27], character 142; Clarke and Norell [136], character 138; Ericson [14],

character 52.

1. Apophysis (if present), continues ventrally beyond symphysis and is dorsoventrally elongate: no (1); yes (2). Noncomparable where apophysis absent. Musser and Clarke [83], character 419.
2. Apophysis (if present), mediolaterally wide: no (1); yes (2). Noncomparable where apophysis absent. Musser and Clarke [83], character 420.
3. Scapus clavicle, mediolateral width: intermediate (1); extremely thick (2). Musser and Clarke [83], character 422; Ericson [14], character 44.
4. Scapus clavicle, ventral curvature: absent (0); present (1). Musser and Clarke [83], character 423.
5. Scapus clavicle, pneumatic foramen on lateral face: absent (0); present (1). Musser and Clarke [83], character 427; Worthy et al. [27], character 143.
6. Scapus clavicle, craniocaudal length: truncate (1); elongate (2). Musser and Clarke [83],

character 425.

1. Scapus clavicle, direction of compression: craniocaudal (1); mediolateral (2). Musser and Clarke [83], character 426.
2. Symphysis, orientation of compression: craniocaudally flattened (1); slightly dorsoventrally flattened (2); completely dorsoventrally flattened (3). Musser and Clarke [83], character 427.
3. Furcula, symphysis, shape: not prominent, in line with furcula (1); rectangular, deviates from symphysis (2).
4. Processus acrocoracoideus, length: truncate (1); elongate (2). Musser and Clarke [83],

character 428.

1. Processus acromialis, length: truncate (1); elongate (2). Musser and Clarke [83], character

429; Clarke and Norell [136], character 88.

1. Processus acromialis, mediolateral orientation: does not deviate from axis of scapus clavicle

(1); lateral (2); medial (3). Musser and Clarke [83], character 431.

1. Processus acromialis, shape: blunt or broadly rounded (1); tapered to a point (2). Worthy et al. [27], character 143.
2. Scapus clavicle, width compared to that of acromion and acrocoracoideus region: more narrow (1); subequal (2); larger (3).
3. Scapus clavicle, width of lateral margin compared to that just lateral to symphysis: more narrow (1); subequal (2); larger (3).

Shoulder Girdle: Scapula

1. Acromion, cranially elongate, reaches cranially beyond other articular faces: no (1); yes (2). Worthy et al. [27], character 89; Clarke and Norell [136], character 133; Ericson [14], character

48; Livezey [13], character 68.

1. Acromion, hooked: no (1); yes (2). Clarke and Norell [136], character 134.
2. Collum and head, width as compared to fac. art. hum.: width of fac. art. hum. subequal to width of collum (1); width of collum at least 3x width of fac. art. hum. (including fac. art. hum.) (2).
3. Collum, medial face, pointed, ventrally oriented projection: absent (0); present (1). Musser and Clarke [83], character 433; Musser and Clarke [77], character 206; see also Musser et al. (2019), character 41 for further discussion.
4. Collum, medial face, large pneumatic foramen: present (1); lost (2). Musser and Clarke [83], character 434; Worthy et al. [27], character 90.
5. Scapus, monotonic ventral curvature general to scapus, regardless of deviation of terminal end: straight to moderate, body and distal margin of scapula is slightly to moderately convex (1); pronounced, body and distal margin of scapula conspicuously convex (2). Musser and Clarke

[83], character 436; Musser and Clarke [77], character 207; Livezey and Zusi [88], character

1260; Clarke and Norell [136], character 131; Ericson [14], character 49.

1. Scapus, lateral face, concavitas longitudinalis: present, concave throughout (1); lost, essentially planar throughout or shallow concavitas limited to cranial and medial portion (2). Musser and Clarke [83], character 437; Musser and Clarke [77], character 208; Livezey and Zusi [88], character 1257.
2. Scapus, ventrolateral face, tubercle of variable size located cranially, often accompanied by pitted crest trailing distally: present (1); lost (2). Musser and Clarke [83], character 438; Musser and Clarke [77], character 209.
3. Dorsal angle: prominent, deviates from body of scapula (1); small or lost, does not interrupt curvature of body of scapula (2). Musser and Clarke [83], character 439.
4. Dorsal angle of scapula, apex, if discernible: cranial to midpoint of shaft (1); at midpoint of shaft (2); caudal to midpoint of shaft (3). Musser and Clarke [83], character 440.
5. Terminal margin, narrowing and/or expansion (regardless of prominence of dorsal angle): none (1); spatulation and expansion of terminus present (2); tapering present (3). Musser and Clarke [83], character 442; Musser and Clarke [77], character 213; Livezey and Zusi [88],

character 1264; Clarke and Norell [136], character 130.

1. Terminal margin, tip, dorsal orientation of terminus: no (1); yes (2). Musser and Clarke [83], character 444.
2. Scapula, length: shorter than humerus (1); as long or longer than humerus (2). Mayr (2004), character 4; Clarke and Norell [136], character 132.

Forelimb: Humerus

1. Tuberculum ventrale and crista m. scapulohumeralis caudalis, general form and caudal prominence: smoothed and often rounded, not very prominent (1); extremely prominent and laterally flattened, projects well beyond rest of bone caudally (2). Musser and Clarke [83], character 445; Musser and Clarke [77], character 214; Worthy et al. [27], character 128 and 142;

Livezey and Zusi [88], character 1364; Ericson [14], character 54.

1. Tuberculum ventrale, proximal elevation regardless of fossa pneumotricipitalis exposure: located inferior to tuberculum dorsale, projects caudally (1); subequal in elevation to that of tuberculum dorsale, projects caudally (2); elevated proximally to (above) tuberculum dorsale, projects caudally or caudoproximally (3). Musser and Clarke [83], character 452; Musser and Clarke [77], character 213.
2. Tuberculum dorsale, proximodistally elongate: no (1); yes, tuberculum dorsale at least 1.5 times as long as it is wide (2). Musser and Clarke [83], character 447; Musser et al. [142], character 65l; Worthy et al. [27], character 125; Mayr and Clarke [81], character 76.
3. Incisura capitis, depth: extremely deep and prominent (1); shallow (2). Musser and Clarke [83], character 448; Musser and Clarke [77], character 215; Livezey and Zusi [88], character

1358.

1. Incisura capitis, distal closure: not closed by additional structures distally (1); enclosed by distal projection of caput humeri (2); closed by transverse ridge (3). Musser and Clarke [83], character 449; Musser and Clarke [77], character 221; Norell and Clakre [86], character 139;

Bertelli et al. [139], character 54; Ericson [14], characters 52 and 53.

1. Incisura capitis, distal closure or homologous site, location: well proximal, incisura truncate

(1); distocaudal, located along same axis as center of shaft (2).

1. Tuberculum ventrale and crista along proximal margin of fossa pneumotricipitalis: typical, flattened craniocaudally (1); domed and distally prominent/overhanging (2).
2. Proximal humerus, attachment site of m. scapulohumeralis cranialis, location relative to crus dorsal fossae: partly on or dorsal to crus dorsal fossa (1); ventral to crus (2). Note: see Hiroshige and Yoshikazu (2007). Worthy et al. [27], character 127.
3. Fossa pneumotricipitalis: pneumatic (1); apneumatic (2). Musser and Clarke [83], character

450; Musser and Clarke [77], character 216; Livezey and Zusi [88], character 1414-1415; Mayr

and Clarke [81], character 83.

1. Fossa pneumotricipitalis, large pneumatic foramen: absent (0); present (1). Mayr and Clarke [81], character 83.
2. Fossa pneumotricipitalis (fossa pneumotricipitalis dorsalis): absent (0); present (1). Musser and Clarke [83], character 451; Worthy et al. [27], character 148.
3. Fossa pneumotricipitalis dorsalis (if present), depth: shallow (1); deep and concave (2). Noncomparable where absent. Musser and Clarke [83], character 452; Worthy et al. [27], character 149.
4. Crista between tuberculum ventrale and capital ridge, deep triangular depression: absent (0); present (1). Based on description in Clarke and Norell [126].
5. Crista between tuberculum ventrale and capital ridge, deep triangular depression if present, location: on caudal surface (1); on ventral surface (2). Noncomparable where absent.
6. Proximal portion of humerus, caudal surface, fossa pneumotricipitalis, crus dorsale fossae: dorsoventrally narrow (1); dorsoventrally broad (2). Musser and Clarke [83], character 453;

Musser and Clarke [77], character 219; Livezey [129], character 202.

1. Head of humerus, craniocaudally flattened: no, globose (1); yes (2). Musser and Clarke [77], character 220; Musser and Clarke [83], character 454.
2. Head, distally reaching apex near ventral tubercle: present (1); lost (2). Musser and Clarke [83], character 455.
3. Head, orientation: horizontal or dorsoventral (1); diagonal (2). Musser and Clarke [83],

character 456.

1. Impressio coracobrachialis: absent or shallow (1); deep (2). Musser and Clarke [77],

character 222; Livezey and Zusi [88], character 1428. Musser and Clarke [83], character 457;

Ericson [14], character 57.

1. Sulcus ligamentosus transversus, dorsoventral length: abbreviate (1); elongate, at least half dorsoventral width of humeral head (2). Musser and Clarke [83], character 458; Musser and Clarke [77], character 223; Livezey and Zusi [88], character 1431.
2. Sulcus ligamentosus transversus, depth: shallow (1); deep (2). Musser and Clarke [83],

character 459; Musser and Clarke [77], character 223; Livezey and Zusi [88], character 1431.

1. Impressio m. pect: shallow or absent (1); deep (2).
2. Bicipital crest, proximodistal length relative to that of deltopectoral crest: less than 1/2 length of deltopectoral crest (1); over 1/2 length of deltopectoral crest (2). Musser and Clarke [83], character 520; Musser and Clarke [77], character 231; Worthy et al. [27], character 122;

Livezey and Zusi [88], character 1383; Bertelli, Giannini and Goloboff [141], character 52.

1. Bicipital crest, terminus on body of humerus, ventral margin: abruptly discontinued proximally on corpus humeri, ventral margin (1); gradually continued by shallow, low but distinct jugum along corpus humeri, ventral margin (2). Musser and Clarke [83], character 521; Musser and Clarke [77], character 227; Livezey and Zusi [88], character 1413.
2. Head of humerus, separation from crista deltopectoralis and tuberculum dorsale ("externus"): pronounced, caput offset and well distinguished from tuberculum dorsale (1); diminished, caput low and poorly distinguished adjacent features (2). Musser and Clarke [83], character 522; Musser and Clarke [77], character 228; Livezey and Zusi [88], character 1354.
3. Crista deltopectoralis, orientation relative to head of humerus: cranial (1); craniolateral (2). Musser and Clarke [83], character 523; Musser and Clarke [77], character 229; Livezey and Zusi [88], character 1374; Clarke and Norell [136], character 142; Ericson [14], character 56.
4. Crista deltopectoralis, shape: rounded (1); trapezoidal (2). Musser and Clarke [83],

character 524.

1. Crista deltopectoralis, comparative proximodistal length relative to that of body of humerus: great, well developed and extending at least 1/3 length of corpus humeri (1); small, diminuative, extending less than 1/3 length of corpus humeri (2). Musser and Clarke [83], character 525; Musser and Clarke [77], character 230; Livezey and Zusi [88], character 1388.
2. Crista deltopectoralis, width compared to that of shaft: less than shaft width (1); same width

(2); dorsoventral width greater than shaft width (3). Clarke and Norell [136], character 143.

1. Proximal humerus, long and narrow accessory scar for m. supracoracoideus: absent (0); present (1). Musser and Clarke [83], character 526; Musser and Cracraft [77], character 61;

Worthy et al. [27], character 126.

1. Proximal section of shaft with triangular cross section: absent (0); present (1). Musser and Clarke [83], character 527; Musser and Clarke [77], character 232; Bertelli et al. [139], character

56.

1. Capital ridge, present and caudally prominent proximal to dorsomedial attachment of crista defining fossa pneumotricipitalis: no (1); yes (2). Worthy et al. [27], character 145.
2. Capital ridge shaft, when present and prominent proximal to dorsomedial attachment of crista defining fossa pneumotricipitalis: directed towards head (1); directed towards region between head and tuberculum dorsale (2); directed towards and/or extends to dorsal tuberosity
3. Noncomparable where not prominent. Worthy et al. [27], character 146.
4. Caudal surface of proximal shaft with centrally positioned, raised muscle attachment scar for m. latissimus dorsi, pars caudalis: absent (0); present (1). Musser and Clarke [83], character 528.
5. Additional, ovate muscle attachment scar for m. latissimus dorsi pars cranialis located along anteriodorsal margin of capital ridge: absent (0); present, an elongate but narrow ovoid depression that is shallow (1); present, round, large, deep ovoid depression (2).
6. Shaft with essentially parallel sides in caudal or cranial aspect: yes (1); no, narrowest near midshaft point (2).
7. Fossa olecrani, depth: limited depth (1); present, markedly deep (2). Musser and Clarke [83], character 529; Musser and Clarke [77], character 236; Worthy et al. [27], character 145;

Livezey and Zusi [88], character 1488.

1. Flexor process, proximodistally elongate: no (1); yes (2). Musser and Clarke [83], character 477; Worthy et al. [27], character 137; Clarke and Norell [136], character 122.
2. Sulcus tendinis m. scapulotricipitalis: absent (0); present, weakly defined, typically broad, shallow and truncate (1); present, conspicuously defined, usually narrow, deep and elongate (2). Not comparable for Spheniscidae. Musser and Clarke [83], character 472; Musser and Clarke [77], character 237; Livezey and Zusi [88], character 1488; Mayr and Clarke [81], character 81;

Clarke and Norell [136], characters 127 and 140.

1. Fossa m. brachialis, dorsoventral position relative to median axis of humerus: ventral (1); medial (2). Musser and Clarke [83], character 473; Musser and Clarke [77], character 239;

Livezey and Zusi [88], character 1520.

1. Impression of brachialis anticus (brachial depression): shallow and small, ovoid, or brachial depression nonexistent (1); deep and part of brachial depression, distal portion of humerus appears to have been "scooped" out (2). Musser and Clarke [83], character 474; Musser and Clarke [77], character 270; Livezey and Zusi [88], character 1456; Mayr and Clarke [81],

character 138.

1. Condylus dorsalis humeri, proximal extent relative to distal margin of fossae m. brachialis: condylus distal to distal terminus of fossa and typically separated from distal margin of fossae m. brachialis by smooth area of bone from latter (1); condylus (proximal margin) typically extending at least proximal to distal margin of fossae m. brachialis (2); condylus markedly proximal to fossa, including extreme proximal condylorum dorsalis and ventralis (3). Musser and Clarke [83], character 475; Musser and Clarke [77], character 274; Livezey and Zusi [88],

character 1447.

1. Condylus ventralis humeri, length: length of long axis of condyle less than the same measure of the dorsal condyle (1); same or greater (2). Clarke and Norell (2001), character 125.

Forelimb: Ulna

1. Processus cotylaris dorsalis, pronounced ventral orientation such that apex processi is approximately coplanar with dorsal face of ulna: absent, apex of process variably elevated dorsally to body of ulna (0); present (1). Not comparable for Spheniscidae. Musser and Clarke [83], character 478; Musser and Clarke [77], character 275; Livezey and Zusi [88], character

1492.

1. Processus cotylaris dorsalis, facies articularis relative to that of cotyla ventralis: less expansive (1); subequal (2). Not comparable for Spheniscidae. Musser and Clarke [83], character 485; Musser and Clarke [77], character 276; Livezey and Zusi [88], character 1496.
2. Processus cotylaris dorsalis, crista intercotylaris: crista rudimentary but evident despite more typically conformed cotylae (1); variably prominent, cotylae dorsalis and ventralis distinct

(2). Musser and Clarke [83], character 4138; Musser and Clarke [77], character 277; Livezey and

Zusi [88], character 1497.

1. Impressio insertii m. brachialis: absent or shallow (1); deep (2). Musser and Clarke [83], character 481; Musser and Clarke [77], character 278; Livezey and Zusi [88], character 1502.
2. Incisura radialis: absent or indistinct (0); present and variably pronounced (1). Musser and Clarke [83], character 488; Musser and Clarke [77], character 285; Livezey and Zusi [88],

character 1505.

1. Trochlea humeroulnaris: no articular facet visible or exhibits shallow articular facet (1); exhibits deep articular facet (2). Musser and Clarke [83], character 483.
2. Impressio scapulotricipitalis, depth: shallow (1); deep (2). Musser and Clarke [83],

character 484.

1. Olecranon process, proximal prominence: prominent (1); limited, at about level of cotylae

(2). Musser and Clarke [83], character 485.

1. Bicipital tubercle: absent (0); present (1). Clarke and Norell [136], character 133.
2. Distal ulna, dorsal aspect, tab-like distally oriented projection along ventromedial margin of labrum condyli dorsalis: absent (0); present (1). Musser and Clarke [83], character 486.
3. Olecranon fossa, depth: shallow (1); deep (2). Musser and Clarke [83], character 487.
4. Depressio radialis, depth: shallow (1); deep (2). Musser and Clarke [83], character 488;

Worthy et al. [27], character 153; Mayr and Clarke [81], character 84.

1. Incisura tub. carp., depth: shallow (1); extremely deep and pit-like (2).

Forelimb: Carpometacarpus

1. Trochlea carpalis, sulcus trochlearis: shallow, rounded in cranial or caudal view, or is somewhat deep laterally but not cranially (1); deep, subangular in cranial or caudal view (2). Musser and Clarke [83], character 490; Musser and Clarke [77], character 251; Worthy et al. [27], character 156; Livezey [129], character 236.
2. Marked ridge between processus pisiformis and trochlea carpalis: absent (0); present (1). Musser and Clarke [83], character 491; Worthy et al. [27], character 161; Livezey [129],

character 272; Mayr (2013), character 27.

1. Fossa infratrochlearis, depth: shallow (1); extremely deep and pit-like (2). Worthy et al. [27], character 160; Clarke and Norell [136], character 152.
2. Extensor process, spur or prominent calcar alae: no (1); yes (2). Livezey [13], character 73.
3. Extensor process, concave facet at terminus: absent (0); present (1).
4. Extensor process, length: elongate (1); truncate (2). Worthy et al. [27], character 163;

Clarke and Norell [136], character 142.

1. Metacarpal 1, proximodistal length: truncate (1); elongate, extends distally onto shaft below head (2).
2. Metacarpal III strongly bowed, delimiting a large spatium intermetacarpale: no (1); yes (2). Musser and Clarke [83], character 492; Mayr (2004), character 7; Mayr and Clarke [81],

character 85; Livezey [13], character 75.

1. Trochlea carpalis, dorsal aspect, labrum dorsalis, proximal terminus of dorsal rim of trochlea: weakly angular or rounded (1); strongly angular, almost pointed, elongated proximally

(2). Musser and Clarke [83], character 493; Musser and Clarke [77], character 252; Livezey

[129], character 238.

1. Metacarpal III, ventral aspect, tuberculum intermetacarpalis (small tubercle on metacarpal III immediately distal to synostosis metacarpalis proximalis): elongate (1); distinct and rounded

(2); obsolete (3). Musser and Clarke [83], character 494; Musser and Clarke [77], character 253;

Worthy et al. [27], character 169; Clarke and Norell [136], character 149; Livezey [129],

character 274; Livezey [13], character 74.

1. Synostosis metacarpalis distalis, dorsal aspect, sulcus interosseus ventralis: present, but shallow (1); lost (2); present, deep (3). Musser and Clarke [83], character 495; Musser and

Clarke [77], character 254; Livezey [129], character 253.

1. Synostosis metacarpalis distalis, synostosis of II and III, craniocaudal length of synostosis: truncate (1); elongate (2). Worthy et al. [27], character 177; Clarke and Norell [136], character

147.

1. Synostosis metacarpalis distalis, synostosis of II and III, craniocaudal length of digits: subequal (1); II distally > III (2); III distally > II (3). Worthy et al. [27], character 172; Clarke and Norell [136], character 148.

Forelimb: Manual Phalanges

1. Manual digit II: phalanx 1, index process: absent or barely protruding (1); present, prominent (2). Musser and Clarke [83], character 496.

Pelvis

1. Pelvis, corpus of first synsacrothoracic vertebra: about equally compressed mediolaterally, as are the corpi of the following vertebrae (1); first synsacrothoracic vertebra significantly more mediolaterally compressed than in the following vertebrae (2). Ericson [14], character 27.
2. Preacetabular iliac blades and synsacrum, fusion: fused (1), unfused (2). Bertelli et al. [139], character 178; Musser and Clarke [83], character 498.
3. Thoracic vertebrae, if fused within preacetabular synsacrum and iliac blades, degree of fusion of transverse processes of thoracic vertebrae to iliac blades: transverse processes of 3 caudalmost thoracic vertebrae incompletely fused to preacetabular iliac blades (1); transverse processes of 1-2 caudalmost thoracic vertebrae incompletely fused to preacetabular iliac blades
4. ; transverse processes of all thoracic vertebrae completely fused to preacetabular iliac blades
5. ; transverse processes of all thoracic vertebrae incompletely fused to preacetabular iliac blades
6. Note: Here incomplete fusion is defined by a lack of fusion across entire or almost entire ventral suture. Noncomparable where iliac blades completely unfused to synsacrum. Musser and Clarke [83], character 499; Musser and Clarke [77], character 281.
7. Preacetabular and postacetabular ilium, dorsal face, relative craniocaudal lengths, indexed by ratio of length of former divided by length of latter: preacetabular ilium greater in craniocaudal length than postacetabular ilium, ratio significantly greater than unity (1); subequal

(2); postacetabular ilium greater in craniocaudal length than preacetabular ilium, ratio less than unity (3). Musser and Clarke [83], character 500; Musser and Clarke [77], character 255; Worthy

et al. [27], character 173; Livezey and Zusi [88], character 1890.

1. Preacetabular ilium, dorsal face, dorsomedial margin, dorsal iliac crest, dorsomedial synostosis forming carina iliaca dorsales: present (1); lost (2). Musser and Cracraft (219), character 256; Livezey and Zusi [88], character 1814; Mayr and Clarke [81], character 852.
2. Carina iliaca dorsales (if present) extent of dorsal fossae created on either side of crista spinosa synsacra: moderate, approximately half or less the length of the carina (1); extremely elongate, extending proximally over half of the carina, typically extending proximolaterally (2); essentially absent (3). Noncomparable where carina iliaca dorsales absent. Musser and Clarke [83], character 503.
3. Preacetabular synsacrum, dorsal prominence of crista spinosa synsacra or carina iliaca dorsales relative to postacetabular synsacrum: subequal in dorsoventral height (1); dorsally prominent and elevated above level of postacetabular synsacrum (2). Musser and Clarke [83], character 504; Musser and Clarke [77], character 257; Livezey and Zusi [88], character 1819.
4. Preacteabular ilium, lateral face, angle respective to transverse plane of synsacrum: oblique or subvertical (1); subhorizontal (2). Musser and Clarke [83], character 505; Musser and Clarke [77], character 258; Livezey and Zusi [88], character 1883.
5. Preacetabular ilium, shape of anterior terminus: subrectangular, does not narrow cranially

(1); rounded (sometimes exhibits incomplete ossification) (2); trapezoidal, narrows cranially (3); triangular, narrows cranially toward apex (4). Musser and Clarke [83], character 506.

1. Preacetabular ilium, anterior terminus, extends anteriorly well beyond proximal margin of synsacrum: no (1); yes (2). Musser and Clarke [83], character 507; Clarke and Norell [136],

character 161.

1. Preacetabular tubercles, status: present (1); lost (2). Musser and Clarke [83], character 508;

Musser and Clarke [77], character 259; Worthy et al. [27], character 184; Livezey and Zusi [88],

character 1813; Mayr and Clarke [81], character 93; Clarke and Norell [136], character 159.

1. Preacetabular tubercles, length: elongate (1); diminutive, close to body of pelvis (2). Noncomparable where absent. Musser and Clarke [77], character 259; Livezey and Zusi [88], character 1813; Mayr ad Clarke (2003), character 93.
2. Preacetabular tubercles, shape of termini: subrectangular (1); acuminate (2). Noncomparable where preacetabulear tubercles absent. Musser and Clarke [83], character 513; Musser and Clarke [77], character 259; Livezey and Zusi [88], character 1813.
3. Acetabular ilium, dorsal face, interacetabular width relative to synsacral length: great, exceeds 1/2 synsacral length (1); moderate, 1/2 to 1/3 synsacral length (2); small, approximately 1/4 synsacral length (3); extremely small, approximately 1/6 synsacral length (4). Musser and Clarke [83], character 514; Musser and Clarke [77], character 260; Worthy et al. [27], character

181; Livezey and Zusi [88], character 1845.

1. Acetabular and postacetabular dorsal synsacrum, osseous sheet covering fenestrae intertransversae synsacrales: yes (1); lost or incomplete along medial or lateral margins (2). Musser and Clarke [83], character 512.
2. Antitrochanter, lateral face: raised dorsally so that it is more visible and robust (1); depressed ventrally so that it is less visible and somewhat obscures the acetabulum (2). Musser and Clarke [77], character 263.
3. Ilioischiadic foramen: absent (0); oblong (1); essentially circular (2). Musser and Clarke

[83], character 515; Musser and Clarke [77], character 265; Worthy et al. [27], character 188;

Livezey and Zusi [88], character 1851.

1. Obturator foramen, closed caudally: no (1); yes (2). Musser and Clarke [83], character 516;

Musser and Clarke [77], character 268; Livezey and Zusi [88], character 1783.

1. Obturator foramen, shape: circular, often with wider dorsoventral margin (1); elongate, ovoid (2). Musser and Clarke [83], character 517; Musser and Clarke [77], character 268;

Livezey and Zusi [88], character 1783.

1. Obturator foramen, axis of orientation: dorsal cranially and ventral caudally (1); craniocaudal or horizontal (2); ventral cranially and dorsal caudally (3). Musser and Clarke [83], character 518; Musser and Clarke [77], character 269.
2. Vertex craniolateralis ilii, dorsally protrudes above antitrochanteric sulcus: no (1); yes (2). Musser and Clarke [83], character 519; Livezey [13], character 84.
3. Vertex craniolateralis ilii, if dorsally protruding above antitrochanteric sulcus, shape: crest- like (1); elongate and tab-like, typically laterally directed (2). Noncomparable where not dorsally protruding. Musser and Clarke [83], character 520.
4. Vertex caudolateralis ilii: tab-like laterally directed (1); a simple crest (2); lost (3); a ventrally directed flange (4). Noncomparable for Paleognathae. Musser and Clarke [83], character 521; Musser and Clarke [77], character 272; Livezey [129], character 286.
5. Postacetabular ilium and ischium, lateral face, fusion: unfused posteriorly (0); fused posteriorly, ilioischiatic fenestra is closed (1). Musser and Clarke [83], character 522; Musser and Clarke [77], character 273; Worthy et al. [27], character 183; Mayr and Clarke [81],

character 94; Clarke and Norell [136], character 154; Cracraft and Clarke [85], character 27;

Livezey [13], character 78.

1. Postacetabular ilium and ischium, lateral face, synchondrosis ilioischiadica: extremely smooth, barely visible or absent (1); line-like and distinctly etched craniocaudally or caudoventrally (2). Noncomparable for Paleognathae. Musser and Clarke [83], character 523; Musser and Clarke [77], character 275.
2. Caudal extent of caudal terminus of postacetabular ilium relative to that of ischium: ilium distinctly cranial to ischium (1); ischium distinctly cranial to ilium (2); subequal (3). Noncomparable for Paleognathae. Musser and Clarke [83], character 525; Musser and Clarke [77], character 278; Livezey and Zusi [88], character 1892.
3. Acetabular synsacrum, ankylosis of acetabular transverse process (often synostotic) to acetabular area beyond lateral margin of synsacrum: absent, transverse process does not ankylose to acetabular area (0); present, ankyloses to dorsal margin of acetabulum, often within dorsocaudal margin of the acetabulum (1); present, ankyloses to craniodorsal margin of ilioischiadic foramen with variable separation between acetabulum and ankylosis, accompanied by sessile concavity between ankylosis and margin of acetabulum (2); present, ankyloses to ventral site between caudal acetabular margin and cranial margin of ilioischiadic foramen, ankylosed to pila that extends from ankyloses between the ilioischiadic foramen toward the dorsal margin of the obturator foramen (3). Noncomparable for Paleognathae. Musser and Clarke [83], character 527; Musser and Clarke [77], character 2138.
4. Acetabular synsacrum, ankylosis of acetabular transverse process (often synostotic) to acetabular area beyond lateral margin of synsacrum, if present: present, ankyloses to dorsal margin of acetabulum, often within dorso-caudal margin of the acetabulum (1); present, ankyloses cranio-dorsal margin of ilioischiadic foramen with variable separation between acetabulum and ankylosis, accompanied by sessile concavity between ankylosis and margin of acetabulum (2); ankylosed to extreme dorsal area between caudal acetabular margin and cranial margin of ilioischiadic foramen, ankylosed to pila that extends from ankyloses between the described foramina and dorsal margin of obturator foramen (3). Noncomparable where absent or where ilioischiadic foramen not closed caudally.
5. Syndesmosis of synsacrum and iliac blades, acetabular and postacetabular portion in dorsal aspect, shape: caudalmost portion more narrow than acetabular portion and appears as a diamond shape (1); width subequeal throughout (2).
6. Preacetabular and acetabular synsacrum, ventral face, ventral sulcus of synsacrum: present

(1); lost or barely discernible (2). Musser and Clarke [83], character 528; Musser and Clarke [77], character 288.

1. Ischium, forked, dorsal process present (1); straight, no dorsal process present (2). Noncomparable where fused posteriorly to close ilioischiadic foramen. Clarke and Norell 2001, character 155.
2. Postacetabular ischium, ventral face, foramen in oblique iliac crest: present (1); lost (2). Musser and Clarke [83], character 529; Musser and Clarke [77], character 283.
3. Fossa renalis, depth: shallow or lost (1); deep (2). Musser and Clarke [83], character 531; Musser and Clarke [77], character 284; Livezey and Zusi [88], character 1853; Clarke and Norell [136], character 163.
4. Lamina of ischium anteriorly extensive so as to create pons for recessus caudalis fossae: yes

(1); no (2). Noncomparable for Paleognathae. Musser and Clarke [83], character 532; Musser

and Clarke [77], character 286; Worthy et al. [27], character 1438; Livezey and Zusi [88],

character 14382; Mayr and Clarke [81], character 95; Ericson [14], character 31; Livezey [13],

character 85.

1. Ischial lamina creating pons for recessus caudalis fossae (if present), visible through ilioischiadic foramen in lateral aspect: no (1); yes (2). Noncomparable where recessus caudalis fossae absent. Musser and Clarke [83], character 533.
2. Recessus caudalis fossae (if present): shallow (1); deep (2). Noncomparable where absent. Musser and Clarke [83], character 534; Musser and Clarke [77], character 286; Worthy et al. [27], character 1438; Livezey and Zusi [88], character 14382; Mayr and Clarke [81], character

95.

1. Caudal margin of postacetabular ilium and in relation to caudal portion of synsacrum: caudal portion of synsacrum separating from and continuing caudally past caudal margin of ilium and splaying laterally towards apex (1); caudal portion of synsacrum fused to caudal margin of ilium completely (2); caudal portion of synsacrum separating from and continuing caudally past caudal margin of ilium and tapered toward apex (3). Musser and Clarke [83], character 536; Musser and Clarke [77], character 289.
2. Caudalmost synsacral vertebrae, fused to costal processes of closest proximal vertebra: no

(1); yes (2). Musser and Clarke [83], character 537.

1. Iliosynsacral incisura, shape: semicircular (1); subrectangular (2). Musser and Clarke [83],

character 539.

1. Processus marginis caudalis, termini invaginated on caudodorsal surface so that termini are medially oriented: no (1); yes (2). Musser and Clarke [83], character 541; Musser and Clarke [77], character 292; Ericson [14], character 33.
2. Processus marginis caudalis, length: diminuitive, close to body of pelvis (1); intermediate

(2); extremely elongate (3).

1. Processus marginis caudalis, exceptional tapering present: no (1); yes (2).
2. Processus marginis caudalis, direction of flattening: mediolateral (1); dorsoventral (2).
3. Processus marginis caudalis, concavity present on lateral or dorsal surface: absent (0); present (1).
4. Processus terminalis ischii, shape: acuminate (1); subrectangular (2); rounded (3). Musser et

al. (2019), character 88. Musser and Clarke [83], character 542.

1. Processus terminalis ischii, length: diminutive, close to body of pelvis (1); intermediate (2); extremely elongate (3).
2. Processus terminalis ischii, direction of terminus: caudal (1); ventral (2).
3. Processus terminalis ischii, ankylosed to pubis: no (1); yes (2). Musser and Clarke [83], character 543.
4. Spatium ischiopubicum, dorsoventral width: wide (1); narrow, ischium and pubis nearly in contact (2). Musser and Clarke [83], character 544; Ericson [14], character 30.
5. Scapus pubis, exclusive of marked departures in apex: recurved, dorsal margin variably concave (1); essentially straight or slightly sigmoid, approximately aligned with major axis of apex of pubis (2); decurved, dorsal margin convex (3). Musser and Clarke [83], character 545; Musser and Clarke [77], character 294; Worthy et al. [27]; character 185; Livezey and Zusi [88],

character 1928.

1. Scapus and apex of pubis, length of extension beyond ischium: pubis not extending beyond caudal margin of ischium or, if it does, extended portion makes up less than one fourth of entire length of pubis (1); portion extending beyond caudal portion of ischium makes up at least one

fourth that of entire length of pubis or more (2). Musser and Clarke [83], character 552; Musser and Clarke [77], character 295; Livezey and Zusi [88], character 2006.

1. Apex pubis, dorsoventral orientation: projecting dorsally (1); projecting ventrally (2). Musser and Clarke [83], character 547; Musser and Clarke [77], character 296; Livezey and Zusi [88], character 1945; Livezey [13], character 81.
2. Apex pubis, dorsomedial form: dorsoventrally spatulate and cruciate (1); spatulation negligible, apex pubis subparallel along ventral and dorsal margins, terminal end subrectangular or acuminate (2). Note: Not to be confused with dorsal angle (can be seen in Rallidae) that is a point of attachment between pubis and ischium. Musser and Clarke [83], character 548; Musser and Clarke [77], character 297; Worthy et al. [27], character 186; Livezey and Zusi [88],

character 1940.

1. Apex pubis, mediolateral curvature: absent or slight (1); present, prominent departure from craniocaudal axis, apex of pubis medial to caudal ischium (2); present, apex of pubis curves lateral to caudal ischium (3). Musser and Clarke [83], character 549.

Ossified Tendons

1. Intratendinous ossification in hindlimbs: absent (0); present (1). Musser and Clarke [77],

character 298; Bertelli et al. [139], character 138. Musser and Clarke [83], character 550. Femur

1. Crista trochanteris, markedly projected cranially: yes (1); no (2). Musser and Clarke [83], character 552; Musser and Clarke [77], character 299; Mayr and Clarke [81], character 97.
2. Crista trochanteris, caudal face distal to facies articularis antitrochantericus: thickening developed (1); lacking distinct, distal thickening (2). Musser and Clarke [83], character 553;

Musser and Clarke [77], character 300; Livezey [129], character 301.

1. Fossa trochantericus: lost or shallow (1); present, deep and extends across entire width of facies articularis antitrochantericus (2). Musser and Clarke [83], character 554; Musser and Clarke [77], character 301; Worthy et al. [27], character 189; Livezey and Zusi [88], character

1978.

1. Collum femoris, facies articularis antitrochanterica, caudal margin: located at 90 degree angle to femoral body (1); projecting proximally (2). Musser and Clarke [83], character 555; Musser and Clarke [77], character 303; Livezey and Zusi [88], character 1975.
2. Proximal femur, pneumatic foramen on craniolateral side: absent (0); present (1). Musser and Clarke [83], character 556; Worthy et al. [27], character 192; Mayr and Clarke [81],

character 98.

1. Proximal femur, large circular depression on caudal face, located laterally near impr. m. obtur.: absent (0); present (1).
2. Proximal femur, large circular depression on caudal face if present, depth: shallow (1); deep
3. Noncomprable where absent.
4. Proximal femur, impr. m. obtur., depth: shallow or absent (1); deep (2).
5. Proximal femur, impr. m. iliotrocant., depth: shallow or absent (1); deep (2).
6. Proximal femur, impr. m. iliotrocant., prominent cristae on either side of scar: diminutive or lost (1); present (2).
7. Fossa poplitea: shallow, weakly delimited (1); deep (2). Musser and Clarke [83], character 558; Musser and Clarke [77], character 306; Worthy et al. [27], character 215; Livezey and Zusi

[88], character 2047.

1. Fossa poplitea, pneumatic foramen: small or absent (1); present, large (2). Musser and Clarke [83], character 559; Musser and Clarke [77], character 307; Worthy et al. [27], character

216; Livezey and Zusi [88], character 2048.

1. Impressio ligamentum collateralis lateralis, depth: shallow (1); deep (2). Musser and Clarke [83], character 560; Worthy et al. [27], character 127.
2. Epicondylus medialis, prominence of tubercle: prominently elevated, typically tubercular

(1); not prominently elevated, grading essentially smoothly (2). Musser and Clarke [83], character 561; Musser and Clarke [77], character 314; Livezey [129], character 308.

1. Epicondylus lateralis, elongate ridge extending proximal from this to roughly half shaft of femur: absent (0); present (1).
2. Condylus lateralis, crista tibiofibularis, distal extent relative to condylus medialis: subequal to condylus medialis (1); distinctly and significantly distal to condylus medialis (2). Musser and Clarke [83], character 562; Musser and Clarke [77], character 312; Worthy et al. [27], character

213; Livezey and Zusi [88], character 2012.

1. Shaft of femur, cranial recurvature, status: absent (0); present (1).
2. Condylus lateralis, caudal aspect, lateromedial width relative to that of condylus medialis: subequal (1); lateral condyle significantly wider (2); lateral condyle significantly more narrow
3. Musser and Clarke [83], character 564; Musser and Clarke [77], character 314; Livezey and

Zusi [88], character 2027.

1. Condylus lateralis, relative proximodistal position of crista tibiofibularis and trochlea fibularis: crista tibiofibularis crest located more proximally than trochlea fibularis (1); subequal in proximal position (2); trochlea fibularis more proximal (3). Musser and Clarke [83], character 565; Musser and Clarke [77], character 315; Worthy et al. [27], character 219; Livezey and Zusi

[88], character 2013.

1. Condylus medialis, caudal aspect, general form: spherical, distal portion greatly rounded, extremely convex (1); triangular, distal portion somewhat rounded (2). Musser and Clarke [83], character 567; Musser and Clarke [77], character 316; Worthy et al. [27], character 212; Livezey

and Zusi [88], character 2030.

1. Crista supracondylaris medialis, medial aspect, form relative to condylus medialis and facies caudalis corporis: absent or rudimentary, condylus not extended by crista, caused by one distinct (often abrupt) angle or incision above the medial condyle, rarely also a second is present (comparatively proximal), interrupting an otherwise gradual curving crest (1); present and prominent, medial condyle continued by crest without interruption by angle or incision above the medial condyle (2). Musser and Clarke [83], character 569; Musser and Clarke [77], character 318; Worthy et al. [27], character 218; Livezey and Zusi [88], character 2042.
2. Fovea tendineus m. tibialis cranialis, depth: shallow (1); deep (2). Musser and Clarke [83], character 570.
3. Femur, length compared to that of tibiotarsus: 1/2 length of tibiotarsus (1); over 1/2 length of tibiotarsus (2); less than 1/2 length of tibiotarsus (3). Musser and Clarke [83], character 572.

Patella

1. Patella, foramen: absent (0); present (1).
2. Patella, size: large, trihedral (1); small, a sliver of bone (2).

Tibiotarsus

1. Cnemial fossa retropatellaris: shallow (1); deep (2). Musser and Clarke [83], character 573;

Musser and Clarke [77], character 321; Livezey and Zusi [88], character 2142.

1. Cnemial fossa retropatellaris, shape: circular (1); elongate and ovoid (2). Musser and Clarke [83], character 574.
2. Cnemial fossa retropatellaris or homologous site, recessus and/or pneumatic foramina (pores): no (1); yes (2). Musser and Clarke [83], character 575; Musser and Clarke [77],

character 322; Livezey and Zusi [88], character 2143.

1. Lateral fossa retropatellaris: shallow (1); deep (2). Musser and Clarke [83], character 576.
2. Lateral fossa retropatellaris or homologous site, recessus and/or pneumatic foramina (pores): no (1); yes (2). Musser and Clarke [83], character 583.
3. Pneumatic foramen underneath jugum between facies articularis lateralis and area interarticularis (typically found distal to incisura, if present): present, 1 (1); present, 2 or more

(2); lost (3). Musser and Clarke [83], character 585; Musser and Clarke [77], character 327. 606. Fossae retropatellares, angle relative to facies articularis medialis and lateralis: on same plane as articular area (1); proximocranially oriented between 45 and 138 degrees (2); proximocranially oriented at approximately 90 degrees so that fossae are perpendicular to articular areas (3). Musser and Clarke [83], character 5138.

1. Crista cnemialis and crista patellaris, apex, proximodistal height relative to head of tibiotarsus: at same height of fossae retropatellares or only slightly elevated above fossae (1); well projected proximally to fossae (2); extremely proximally projected, at least 3 times the length of the fossae above the fossae (3). Musser and Clarke [83], character 581; Worthy et al. [27], character 222; Mayr and Clarke [81], character 99; Livezey [13], character 85.
2. Crista cnemialis lateralis apex, proximodistal height relative to head of tibiotarsus: essentially at level of fossae retropatellares (1); proximal to fossae retropatellares (2); extremely proximally projected, at least 3 times the length of the fossae above the fossae (3); extremely distally projected (4). Musser and Clarke [83], character 588.
3. Crista cnemialis lateralis and cranialis, distal apices (distal angles of cristae, not attachment locations on shaft of tibiotarsus), relative proximodistal heights: subequal (1); apex of crista cnemialis lateralis located proximally to that of crista cnemialis cranialis (2); apex of crista cnemialis cranialis proximal to that of crista cnemialis lateralis (3). Musser and Clarke [83], character 583.
4. Crista cnemialis cranialis, pronounced lateral curvature (especially proximally): present (1); lost (2). Musser and Clarke [83], character 584; Musser and Clarke [77], character 329; Worthy

et al. [27], character 225; Livezey and Zusi [88], character 2088.

1. Crista cnemialis lateralis, shape: hamate but intermediate, but hook rounded and does not protrude beyond jugum of lateral crista (1); more prominent and hamate, acuminate hook protrudes distolaterally beyond lateral crest jugum (2); rounded, tuberculate (3). Musser and Clarke [83], character 585.
2. Crista cnemialis cranialis, especially cranially projected: no, but is still well projected (1); yes (2); no, extremely reduced (3). Musser and Clarke [83], character 586.
3. Crista cnemialis cranialis, apex of crista cnemialis cranialis and crista patellaris or apex of crista cnemialis cranialis only, shape: apex lost (0); rounded (1); acuminate (2). Musser and Clarke [83], character 588.
4. Crista cnemialis cranialis, concavity of lateral and medial surfaces: both surfaces concave

(1); medial surface convex, lateral surface concave (2); both surfaces convex (3); medial surface concave, lateral surface convex (4). Musser and Clarke [83], character 589; Musser and Clarke [77], character 328; Livezey and Zusi [88], character 2078.

1. Crista cnemialis cranialis, anteriorly raised crista that descends at least 1/3 of shaft: lost (1); present (2). Note: not to be confused with muscular linea.
2. Crista cnemialis lateralis, concavity of lateral and medial surfaces: both surfaces concave

(1); medial surface convex, lateral surface concave (2); both surfaces convex (3); medial surface concave, lateral surface convex (4). Musser and Clarke [83], character 590; Musser and Clarke [77], character 328; Livezey and Zusi [88], character 2078.

1. Crista cnemialis lateralis, proximal crista, width compared to crista patellaris: extremely wide (1); narrow, subequal in width to crista patellaris (2). Musser and Clarke [83], character 591.
2. Lateral fossa retropatellaris, extremely reduced: no (1); yes (2). Musser and Clarke [83], character 592.
3. Facies articularis medialis: convex (1); concave (2). Musser and Clarke [83], character 593.
4. Crista between fossae retropatellares: absent (0); present (1). Musser and Clarke [83],

character 594.

1. Facies articularis lateralis, concavity: absent or small (1); present, large (2). Musser and Clarke [83], character 595.
2. Interarticular area: convex (1); concave (2). Musser and Clarke [83], character 596.
3. Crista cnemialis lateralis, lateral prominence relative to articulated fibula: not lateral to fibular head (1); lateral to fibular head (2). Musser and Clarke [83], character 597; Musser and Clarke [77], character 331; Livezey and Zusi [88], character 2094.
4. Cristae cnemiales cranialis and lateralis, comparative distal extents on shaft of tibiotarsus: crista cnemialis cranialis terminating distinctly distal to crista cnemialis lateralis (1); cristae subequal in distal extent (typically truncated), both lacking jugae (2). Musser and Clarke [83], character 598; Musser and Clarke [77], character 332; Worthy et al. [27], character 229; Livezey

and Zusi [88], character 2099.

1. Crista cnemialis cranialis, recurvature of distal margin: absent (0); present (1). Musser and Clarke [83], character 599.
2. Fibular crest, length: over 1/4 length of tibiotarsus (1); about 1/4 length of tibiotarsus (2); less than 1/4 length of tibiotarsus (3). Musser and Clarke [83], character 601; Worthy et al. [27], character 230.
3. Fibular crest, lateral prominence: little to none (1); prominently projected (2). Musser and Clarke [83], character 602.
4. Foramen interosseum proximale: sublinear and incisurate, relatively narrow (1); approximately ovate, relatively spacious (2). Musser and Clarke [83], character 605; Musser and Clarke [77], character 335; Livezey and Zusi [88], character 2128.
5. Foramen interosseum distale, length as compared to length of the tibiotarsus: more than 1/4 length of the tibiotarsus (1); less than 1/4 length of tibiotarsus (2); exactly 1/4 length of tibiotarsus (3). Musser and Clarke [83], character 606; Musser and Clarke [77], character 336;

Livezey and Zusi [88], character 2129.

1. Proximal shaft of tibiotarsus, cranial aspect: rounded (1); craniocaudally flattened and mediolaterally broad (2). Musser and Clarke [83], character 607.
2. Epicondylus medialis: pronounced tubercle (1); lost or diminutive (2). Musser and Clarke [83], character 608; Musser and Clarke [77], character 337; Worthy et al. [27], character 232. 632. Epicondylus lateralis: lost or diminutive (1); pronounced tubercle (2). Musser and Clarke [83], character 609.
3. Trochlea cartilaginis tibialis, lateral and medial margins: splayed laterally (1); medially compressed (2); only medial margin splayed laterally (3). Musser and Clarke [83], character 613; Musser and Clarke [77], character 338.
4. Condylus lateralis, depressio epicondylaris lateralis: shallow or absent (1); deep (2). Musser and Clarke [83], character 613; Musser and Clarke [77], character 341.
5. Condylus lateralis, depressio epicondylaris lateralis, accessory depression located along caudal margin of condylus lateralis: absent (0); present (1). Musser and Clarke [83], character 614; Musser and Clarke [77], character 341.
6. Condylus lateralis, depressio epicondylaris lateralis, accessory depression (if present), depth: shallow (1); deep (2). Noncomparable where depressio epicondylaris lateralis absent. Musser and Clarke [83], character 615.
7. Condylus medialis, depressio epicondylaris medialis: shallow or absent (1); deep (2). Musser and Clarke [83], character 617.
8. Condylus medialis, depressio epicondylaris medialis, accessory depression (if present), depth: shallow (1); deep (2). Noncomparable where depressio epicondylaris absent. Musser and Clarke [83], character 619.
9. Condylus medialis, medial deflection: no (1); yes (2). Worthy et al. [27], character 274;

Livezey [13], character 87.

1. Condylus medialis and condylus lateralis, relative positions: pulled in medially (1); spread laterally (2). Musser and Clarke [83], character 620; Worthy et al. [27], character 275; Livezey

and Zusi [88], character 2142.

1. Ossified pons supratendinous: absent (0); present (1). Worthy et al. [27], character 272;

Mayr and Clarke [81], character 130; Clarke and Norell [136], character 1438; Ericson [14],

character 68.

1. Tuberositas retinaculi extensorius lateralis, if ossified retinaculum absent, comparative prominence of lateral and medial cristae: subequal (1); medial crista more prominent (2); lateral crista more prominent (3). Musser and Clarke [83], character 623.
2. Tubercle laterodistal to pons supratendinous: absent (0); present (1). Mayr and Clarke [81], character 131. Musser and Clarke [83], character 627; Mayr and Clarke [81], character 131.
3. Tubercle laterodistal to pons supratendinous (if present), prominence: diminutive (1); prominent (2). Noncomparable where tubercle absent. Musser and Clarke [83], character 625; Mayr and Clarke [81], character 131.
4. Crista extending proximally from tubercle laterodistal to pons supratendinous or homologous site: absent (0); present (1). Musser and Clarke [83], character 626.
5. Crista extending proximally from tubercle laterodistal to pons supratendinous or homologous site (if present), length: proximodistally truncate (1); proximodistally elongate (2). Noncomparable where crista absent. Musser and Clarke [83], character 627.
6. Crista extending proximally from tubercle laterodistal to pons supratendinous or homologous site (if present), foramen at base: absent (0); present (1). Noncomparable where crest absent. Musser and Clarke [83], character 628.
7. Medial and lateral condyles, relative cranial extensions (compare looking at distal surface): subequal (1); medial condyle extended more cranially (2). Musser and Clarke [83], character 630; Worthy et al. [27], character 235; Clarke and Norell [136], character 185; Livezey [13],

character 86.

1. Medial and lateral condyles, relative mediolateral thickness (compare looking at distal surface): subequal (1); medial condyle mediolaterally thinner (2); lateral condyle thinner (3). Musser and Clarke [83], character 631; Clarke and Norell [136], character 188.
2. Tuberositas retinaculi extensorius medialis (if present), form: tab-like, prominent (1); small and diminutive crest (2). Noncomparable where absent. Musser and Clarke [83], character 632. 651. Tuberositas retinaculi extensorius medialis (if present), craniomedial orientation: oriented medially (1); oriented cranially (2). Noncomparable where absent. Musser and Clarke [83], character 633.
3. Tuberositas retinaculi extensorius lateralis, extreme lateral extension: no (1); yes (2). Musser and Clarke [83], character 636; Worthy et al. [27], character 277.
4. Pons supratendinous, location of distal opening: above medial condyle (1); just lateral to medial condyle but within medial margin of tibiotarsus (2); centered along midline (3). Musser and Clarke [83], character 637; Worthy et al. [27], character 273; Ericson [14], character 69.
5. Canalis extensorius, depth: shallow (1); deep (2); extremely deep and cavernous (3). Musser and Clarke [83], character 638.
6. Medial and lateral condyles, relative proximal extents: lateral condyle located more proximally than medial condyle (1); subequal (2); medial condyle located more proximally than lateral condyle (3). Musser and Clarke [83], character 639.
7. Length of Tibiotarsus compared to that of tarsometatarsus: tarsometatarsus roughly half length of tibiotarsus or less (1); subequal or tarsometatarsus well over half of tibiotarsus (2). Livezey [13], character 88.

Tarsometatarsus

1. Calcaneum: small, unfused to ascending process (1); large, fused to ascending process (2). Cracraft and Clarke [85], character 28.
2. Medial cotyle, dorsolateral margin margin, exceptionally projected proximally and crista- like: no (1); yes (2). Musser and Clarke [83], character 641.
3. Medial cotyle, plantar margin, exceptionally projected proximally and crista-like: no (1); yes, moderately projected to roughly the same level of the proximal margin of the hypotarsal eminence (2); yes, prominently projected proximal to hypotarsal eminence (3). Musser and Clarke [83], character 642.
4. Medial cotyle, proximodistal orientation of dorsal margin: cotyla perpendicular to shaft of tarsometatarsus, proximal surface of cotyla not visible in dorsal aspect (1); dorsal margin of cotyla positioned slightly more distally than that dorsal half of cotyla somewhat visible in dorsal aspect (2); dorsal margin of cotyla positioned much more distally than that of plantar margin so that cotyla is largely visible in dorsal aspect (3). Musser and Clarke [83], character 643.
5. Lateral cotyle, dorsal margin, crista-like margin and/or subtending angle present: yes (1); no, dorsal margin of cotyle essentially absent and cotyla is coplanar with and almost indistinguishable from cotyla corpus (2). Musser and Clarke [83], character 644.
6. Lateral cotyle, depth: deep, concave (1); flat or only slightly concave (2). Musser and Clarke [83], character 645.
7. Lateral cotyle, proximodistal orientation of dorsal margin: dorsal margin of cotyla positioned slightly more distally than that dorsal half of cotyla somewhat visible in dorsal aspect

(1); cotyla perpendicular to shaft of tarsometatarsus, proximal surface of cotyla not visible in dorsal aspect (2); dorsal margin of cotyla positioned much more distally than that of plantar margin so that cotyla is largely visible in dorsal aspect (3). Musser and Clarke [83], character 652.

1. Hypotarsal eminence, proximally prominent: no (1); yes (2). Musser and Clarke [83],

character 647; Worthy et al. [27], characters 254 and 255.

1. Lateral and medial cotylae (dorsal perspective), relative proximal elevation: cotyla lateralis distinctly distal to cotyla medialis (1); cotyla lateralis subequal to cotyla medialis (2); cotyla medialis distinctly distal to cotyla lateralis (3). Musser and Clarke [83], character 648; Musser and Clarke [77], character 352; Livezey and Zusi [88], character 2250.
2. Fossa parahypotarsalis lateralis, depth: shallow or barely visible (1); deep (2). Musser and Clarke [83], character 649; Musser and Clarke [77], character 347; Livezey and Zusi [88],

character 2258.

1. Fossa parahypotarsalis medialis, depth: shallow or barely visible (1); deep (2). Musser and Clarke [83], character 650; Musser and Clarke [77], character 348; Wrothy et al. (2017),

character 261; Livezey and Zusi [88], character 2257.

1. Hypotarsus, crista(e) medialis hypotarsi and crista(e) lateralis hypotarsi, relative plantar prominence: crista(e) medialis hypotarsi more plantarly prominent than crista(e) lateralis hypotarsi (1); crista(e) subequal in plantar prominence (2); crista(e) medialis hypotarsi less plantarly prominent than crista(e) lateralis hypotarsi (3). Musser and Clarke [83], character 651; Musser and Clarke [77], character 349; Livezey [129], character 336.
2. Crista medialis hypotarsi and crista lateralis hypotarsi, relative distal extents and magnitude: distal-most terminus(i) of medial crest(s) much more distally extensive, lateral crista(e) proximodistally truncate, about 1/2-2/3 proximodistal length of the medial crista (1); subequal

(2); distal-most terminus(i) of lateral crest(s) more distally extensive, medial crista(e) proximodistally truncate, approximately 1/2 proximodistal length of the lateral crista, and reduced to a mediolaterally thin, osseous sheet (3); distal-most terminus(i) of lateral crest(s) more distally extensive, medial crista(e) proximodistally truncate, reduced to at least approximately 1/3 or less proximodistal length, thin lamina (4). Musser and Clarke [83], character 652.

1. Hypotarsus, tendon of m. flexor digitorum longus (FDL) enclosed by a canal: no, sulcus present for m. (1); m. enclosed in a canal (2). Noncomparable where muscle absent or sulcus/canal for muscle absent/indiscernible, or where homology is uncertain. Musser and Clarke [83], character 653; Musser and Clarke [77], character 350; Livezey and Zusi [88], characters

2284-2286; Mayr and Clarke [81], character 133.

1. Hypotarsus, canal for tendon of m. flexor digitorum longus (FDL, if present), centrally located: no (1); yes (2). Noncomparable where not enclosed in canal. Musser and Clarke [83], character 654.
2. Hypotarsus, tendon of m. flexor perforatus digiti II (FPII) enclosed in a canal: no, sulcus present for m. (1); m. enclosed in a canal (2). Noncomparable where muscle absent or sulcus/canal for muscle absent/indiscernible, or where homology is uncertain. Musser and Clarke [83], character 655; Musser and Clarke [77], character 352; Bertelli et al. [139], character 75.
3. Hypotarsus, tendon of m. flexor hallicis longus (FHL) enclosed in a canal: no, sulcus present for m. (1); m. enclosed in a canal (2). Noncomparable where muscle absent or

sulcus/canal for muscle absent/indiscernible, or where homology is uncertain. Musser and Clarke [83], character 657; Mayr and Clarke [81], character 136.

1. Hypotarsus, major hypotarsal ridge, distal end: markedly hooked caudodistally forming notch, includes if joined to crista plantaris medialis (1); ridge terminates abruptly, drops steeply to shaft (2); ridge terminates by gradually lowering to shaft (3). Worthy et al. [27], character 258. 675. Hypotarsus, number of ridges separating grooves: 1 (1); 2 (2); 3 (3). Noncomparable where absent or only one large canal is present.
2. Hypotarsus, depth of grooves: 3 subequally deep grooves present (1); 1 deep groove or canal and up to 2 shallow grooves present (2); all grooves shallow (3).
3. Fossa infracotylaris dorsalis, depth: shallow (1); deep (2). Musser and Clarke [83], character 658; Musser and Clarke [77], character 354; Livezey and Zusi [88], character 2259; Mayr and

Clarke [81], character 134.

1. Proximal tarsometatarsus, dorsal aspect (medial portion), arcus extensiorius (ossified retinaculum): not present, remains of retinaculum are only impressio retinaculi extensorii (1); present (2). Musser and Clarke [83], character 660; Mayr (2004), character 35.
2. Proximal portion of tarsometatarsus, dorsal aspect, foramina vascularia proximalia: essentially equal in height (1); lateral foramina distinctly distal to medial foramina (2); medial foramina distal to lateral foramina (3). Musser and Clarke [83], character 661; Musser and Clarke [77], character 353; Livezey and Zusi [88], character 2264.
3. Sulcus extensorius, proximal portion medial to dorsal foramina vascularia proximalia, depth: essentially absent (1); present, shallow (2); present, deep (3). Musser and Clarke [83], character 662.
4. Sulcus extensorius, proximal portion lateral to dorsal foramina vascularia proximalia, depth: essentially absent (1); present, shallow (2); present, deep (3). Musser and Clarke [83], character 663.
5. Dorsal foramina vascularia proximalia, enclosed in ovoid depression that is plantar to plane of sulcus extensorius: yes (1); no, foramina coplanar with sulcus extensorius (2); yes, but only lateral foramen is plantar to sulcus extensorius (3). Musser and Clarke [83], character 664; Musser and Clarke [77], character 354; Livezey and Zusi [88], character 2259.
6. Proximal portion of tarsometatarsus, dorsal aspect, additional foramen or pair of circular foramina proximal to foramina vascularia proximalia that do not open to plantar face: absent (0); present, one foramen (1); present, pair of foramina (2). Musser and Clarke [83], character 667.
7. Proximal portion of tarsometatarsus, plantar aspect, foramina vascularia proximalia, number: a pair is present (1); a pair is present with an additional foramen located on the lateral side (2); only one foramen is present (3); a pair is present with at least an additional foramen located on the medial side (4). Musser and Clarke [83], character 668; Musser and Clarke [77], character 355; Livezey and Zusi [88], character 2264.
8. Proximal portion of tarsometatarsus, plantar aspect, foramina vascularia proximalia: lateral foramina distinctly distal to medial foramina (1); lateral and medial foramina about equal in height (2). Noncomparable where only one foramen is present. Musser and Clarke [83], character 669; Musser and Clarke [77], character 356. Ksepka and Clarke (2012), character 78.
9. Sulcus extensorius, depth along shaft of tarsometatarsus: shallow or absent (1); deep sulcus depth is roughly half that of tarsometatarsus (2); cavernous, only planter lamina of tarsometatarsus present (3). Musser and Clarke [83], character 670; Musser and Clarke [77],

character 357; Worthy et al. [27], character 262; Livezey and Zusi [88], character 2305.

1. Sulcus extensorius, medial and lateral cristae, relative dorsal prominence: medial crista more dorsally prominent than lateral crista (1); subequal (2); lateral crista more dorsally prominent than medial crista (3). Musser and Clarke [83], character 677.
2. Tuberositas m. tibialis cranialis, dorsal prominence: prominent and raised dorsal to sulcus extensorius (1); diminutive, barely raised dorsally (2). Musser and Clarke [83], character 672;

Worthy et al. [27], character 264.

1. Tuberositas m. tibialis cranialis, number: 1 (1); 2 (2). Noncomparable for taxa with scoring

(1) for character (canal) due to fusion of tuberosities. Musser and Clarke [83], character 673; Worthy et al. [27], character 263.

1. Metatarsal II, plantar aspect, fossa for metatarsi I: shallow notch (1); conspicuous ovoid fossa (2). Clarke and Norell [136], character 196; Worthy et al 2017, character 277; Livezey

1997, character 91.

1. Processus calcaris: absent or diminutive (1); present (2). Musser and Clarke [83], character 676; Worthy et al. [27], character 272; Livezey and Zusi [88], character 2312.
2. Vasculare distale, plantar opening: opens flush onto plantar surface (1); directed distoplantarly, so partially recessed into incisura intertrochlearis lateralis (2). Worthy et al. [27], character 283; Livezey [13], character 92.
3. Fossa supratrochlearis plantaris: absent or indistinct (0); present, distinctly concave (1). Musser and Clarke [83], character 683; Musser and Clarke [77], character 363; Livezey and Zusi [88], character 2329.
4. Trochlea metatarsi II, plantarly prominent eminentia (medio) plantaris (ala): absent (0); present (1). Musser and Clarke [83], character 678; Worthy et al. [27], character 276; Livezey

and Zusi [88], character 2352.

1. Trochlea metatarsi II, eminentia (medio) plantaris (ala), form: rounded (1); subrectangular

(2). Noncomparable in absence of ala. Musser and Clarke [83], character 685; Musser and Clarke [77], character 364; Livezey and Zusi [88], character 2352.

1. Trochlea metatarsi II, small, tab-like eminence present proximal to sulci (not ala): absent (0); present (1).
2. Trochlea metatarsi III, proximodistal length as compared to that of body of tarsometatarsus: length from proximal to distal end is less than 1/5 length of entire tarsometatarsus from top of eminentia intercotylaris to distal end of trochlea metatarsi III (1); length from proximal to distal end is 1/5 or greater length of entire tarsometatarsus (2). Musser and Clarke [83], character 681; Musser and Clarke [77], character 365.
3. Trochlea metatarsi III, plantar aspect, proximal extent of lateral and medial edges of trochlea: trochlear edges approximately equal in proximal extent (1); lateral edge extends farther

(2). Clarke and Norell [136], character 201.

1. Trochlea metatarsi IV, foveae ligamentorum collateralium, status and form sensu depth and width relative to that of associated trochlea: present, moderate depression (1); small, shallow, almost absent (2). Musser and Clarke [83], character 688; Musser and Clarke [77], character 366;

Livezey and Zusi [88], character 2349.

1. Trochleae metatarsalia II-IV, relative distal extents: II<III>=IV and II<IV (1); II<III>IV and II subequal to IV (2); II>IV, III>=IV (3). Musser and Clarke [83], character 684; Worthy et al. [27], character 273; Clarke and Norell [136], character 202; Livezey [13], character 94.
2. Distal portion of tarsometatarsus, trochlea metatarsi II, facies articularis phalangealis, sulcus trochlearis (narrow groove between lateral and medial rims of trochlea): remains distinct on facies dorsalis (1); obsolete on facies dorsalis, terminating medially toward fovea ligamentorum

collaterallium at distal apex of trochlea (2). Musser and Clarke [83], character 685; Musser and Clarke [77], character 368; Worthy et al. [27], character 274; Clarke and Norell [136], character

197; Livezey [129], character 354.

1. Trochlea metatarsi II, plantarly deflected: no (1); yes (2). Mayr and Clarke [81], character

138. Musser and Clarke [83], character 686; Clarke and Norell [136], character 138.

1. Trochlea metatarsi IV, plantar ala: not prominent, plantar extent significantly less than that of trochlea metatarsi II (1); conspicuously prominent, plantar extent subequal to that of trochlea metatarsi II (2). Note: not to be confused with flange on plantar surface of trochlea metatarsi II, common in many genera. Musser and Clarke [83], character 687.
2. Pedal digit IV, phalanges 3 and 4, relative lengths: phalanx 4 longer (1); both phalanges of equal length (2); phalanx 4 shorter (3). Bertelli et al. [139], character 76. Musser and Clarke [83],

character 688.

1. Pedal I:1, length as compared to III:1: not as follows (1); I:1 about half the length of III:1

(2). Musser and Clarke [83], character 691; Mayr and Clarke [81], character 143; Livezey [13],

character 96.

1. Proximal end of pedal II:2 extending little beyond proximal end of III:2: no (1); yes (2). Musser and Clarke [83], character 693.
2. Feet, webbed or lobed: no (1); yes, fully webbed and palmate (2). Worthy et al. [27], character 285; Mayr and Clarke [81], characters 141 and 142.
3. Musculus femorotibialis externus, distal head: absent (0); present (1). Mayr and Clarke [81], character 147; McKitrick [143], character 8.
4. Musculus flexor cruris lateralis, pars accessoria (‘‘Y’’ muscle in the formula of George and Berger, 1966, Tab. IX.1): absent (0); present (1). Mayr and Clarke [81], character 148;

McKitrick [143], character 8.

1. Syrinx, presence of ossified pessulus: absent (0); present (1). Worthy et al. [27], character 288; also based on descriptions and CT scans presented in Clarke et al. [40].
2. Syrinx, asymmetry at the tracheobronchial juncture: no (1); yes (2). Worthy et al. [27], character 289; also based on descriptions and CT scans presented in Clarke et al. [40].
3. Habitat: terrestrial (1); aquatic (2); semiaquatic (3). Based on ecomorphological attribute characters of Livezey [13].
4. Swimming mode: non-swimmer (1); foot propelled (2); wing propelled (3); surface swimmer (4); plunger (5); foot and wing propelled (6). Based on swimming mode categories of Hinic-Frlog and Motani [102].
5. Diet: primarily herbivorous (1); omnivorous (2). Based on ecomorphological attribute characters of Livezey [13].
6. Lamellae for filter feeding: absent (0); present (1); present but vestigial (2).
7. Lamellae for filter feeding: absent (0); present (1).
8. Feeding mode: mixed feeders (1); diving graspers (2); filter-feeding (3); grazer (4). Based on feeding modes interpreted from thorough literature compilation presented in Li and Clarke [17].
9. Feet, webbed: none (1); present (2).
10. Pedal III, subequal to or longer than tarsometatarsus: yes (1); no (2).

**References**

1. Clarke JA, Norell MA. The Morphology and Phylogenetic Position of *Apsaravis ukhaana* from the Late Cretaceous of Mongolia. Am Mus Novit. 2002; 2002: 1-46.
2. Livezey BC. A phylogenetic analysis of the Gruiformes (Aves) based on morphological characters, with an emphasis on the rails (Rallidae). Philos Trans R Soc Lond B Biol Sci. 1998; 353: 2077–2151. DOI: 10.1098/rstb.1998.0353
3. Cracraft J. The lacrimal-ectethmoid bone complex in birds: a single character analysis. The Am Midl Nat. 1968; 80: 31-359.
4. Bertelli S, Chiappe LM, Mayr G. A new Messel rail from the early Eocene fur Formation of Denmark (Aves, Messelornithidae). J Syst Paleontol. 2011; 9: 551–562.
5. Bertelli S, Chiappe LM. Earliest tinamous (Aves: Palaeognathae) from the Miocene of Argentina and their phylogenetic position. Contributions in Science. 2005; 502: 1-20.
6. Bertelli S, Giannini NP, Goloboff PA. A phylogeny of the Tinamous (Aves: Palaeognathiformes) based on integumentary characters. Syst Biol. 2002; 51: 959-979.
7. Musser G, Ksepka DT, Field DJ. New Material of Paleocene-Eocene *Pellornis* (Aves: Gruiformes) Clarifies the Pattern and Timing of the Extant Gruiform Radiation. Diversity. 2019; 11: 102. DOI: 10.3390/d11070102.
8. McKitrick M. Phylogenetic analysis of avian hindlimb musculature. Miscellaneous Publications, The Museum of Zoology, University of Michigan*.* 1991; 179: 1-85.
