# Supplementary material for: A new Paleogene fossil and a new dataset for waterfowl (Aves: Anseriformes) clarify phylogeny, ecological evolution, and avian evolution at the K-Pg Boundary
Source: PLoS One. 2024 Jul 30;19(7):e0278737. doi: 10.1371/journal.pone.0278737 (PMC11288464; doi:10.1371/journal.pone.0278737)
Supplement: Attachment — Submitted filename: Response_to_Reviewers.pdf [file pone.0278737.s011.pdf]

Response to Reviewers-Our responses have been recorded in red text.

Reviewer #1: You correctly note that I confused your phylogenetic placement in my previous comments for which I apologize. However, this confusion stems from your statement in the abstract (line 56) that says "We recover the new Eocene taxon as a stem anseranatid across all analyses". In line 696 you also note that seven characters "support placement of P. grandei as a stem anseranatid". In your response to the reviewers you also note that the figures of the "resulting trees clearly show that we recover Paakniwatavis grandei as a stem anseranatid across all analyses". I find this very confusing. In the tree, the new taxon is the sister group of the clade (Anseranatidae + Anatidae) - do you use the term Anseranatidae for this clade (which would be very strange and conflict with your use of Anseranatidae in the figures), or is this a lapsus? Based on the phylogenetic trees, the new fossil is a stem group representative of the clade (Anseranatidae + Anatidae), not a stem anseranatid.

We apologize and thank the Reviewer for the clarification—we have now changed all instances of "stem anseranatid" to reflect its placement as a sister taxon to the clade (Anseranatidae + Anatidae).

My comments on the "duck-like" beak were based on my erroneous assumption that you found the new taxon to be within the clade (Anseranatidae + Anatidae), as a "stem anseranatid" (as repeatedly note in the paper). In this case, the presence of a galliform-like beak would conflict with the duck or goose-like beaks of anseranatids and anatids. Of course, my comments is not valid if the new taxon is outside the clade (Anseranatidae + Anatidae).

We thank the Reviewer for this clarification.

Your statement that "Seven unambiguous and three ambiguous optimized synapomorphies of the quadrate, mandible, furcula, scapula, carpometacarpus, tarsometatarsus and pedal phalanges support placement of P. grandei as a stem anseranatid" is not a standard one used in phylogenetic analyses. Apart from the stem anseranatid vs. stem (anseranatid + anatid) issue, your characters can only support an assignment of the taxon to a certain clade. In order to show that it belongs to the stem group of this clade, you would need to list characters that diagnose the crown group of this clade and that are absent in the fossil.

We thank the Reviewer for this clarification and have now changed the wording to reflect the placement of this taxon.

In my first review, I have also commented on screamer-like fossil from North America and Europe, which you omitted from your manuscript. These have meanwhile been described as Anachronornis and Danielsavis: Houde, P., Dickson, M., & Camarena, D. (2023). Basal Anseriformes from the Early Paleogene of North America and Europe. Diversity, 15(2), 233. In this latter paper, comparisons with the Green River Fossil (Paakniwatavis) were made, and I think you should at least briefly address this study in your paper. On the one hand, the study addresses the fossil you describe, on the other it describes fossils that are possibly related to your new species. Your new taxon also needs to be diagnosed from Anachronornis and Danielsavis.

We agree that this study should be addressed. We have since included these new anseriform taxa within the morphological matrix and rerun the analyses; however, these taxa did not

change the position of any included fossils. Due to this we have added discussion regarding the new fossils and these new results throughout the manuscript but have only included these trees (and the matrix that includes these taxa) within the supplement on Morphobank.

In your response to the authors you note that Gastornis is a junior synonym of Diatryma. This is certainly not true: Gastornis was described by Hebert 1855 whereas Diatryma is authored by Cope 1876. Gastornis therefore clearly precedes Diatryma by more than two decades.

We apologize for the error and have now changed all instances of *Diatryma* to *Gastornis*.

Reviewer #2: Please refer to attachment for my full review. I paste the first paragraph only here to fulfill the minimum 100 character requirement.
Every novel early Paleogene bird fossil is important in its own right, and this manuscript represents a significant investment of solid work to describe one. That being said, it is unfortunate that the revised manuscript still leaves a great deal to be desired in its current form. The most fundamental among its conceptual flaws are overstated conclusions drawn from ancestral state reconstruction that itself is dependent on a suspect phylogeny. The many origins of these flaws are addressed below.
Continued in attachment.

The authors are justified in presenting the results of their phylogenetic analysis whatever its outcome. The recovery of *Paakniwatavis* as sister to *Anseranas* is not entirely implausible, but I am skeptical of it. Conflicting positions of the included fossil taxa (excluding *Paakniwatavis*) have been obtained within and among analyses by other authors, and there is no bootstrap support for their placement in the current analysis (648). The authors write, "The morphological data matrix is built on that of [71][77]", matrices that were developed for Gruiformes and Charadriiformes, not Anseriformes. The authors subsequently state that eight additional anseriform-specific data matrices "were evaluated for use in this iteration of the dataset" but it is unclear to me what is meant by this statement, and particularly by "iteration".

Paakniwatavis was placed as the sister taxon to Anseranas+Anatidae with 85% bootstrap support in the combined results (Figure 5). Bootstrap support within morphological results is less than 50% which is typical for extinct taxa included in analyses that are only based on morphological data. Our matrix and justifications for the taxa and characters included is thoroughly explained within the manuscript.

First, I will address problems with the phylogenetic analysis.
Foremost, I re-ran the morphological data through parsimony analysis and did not recover the same tree described in this paper or depicted in Fig 5. Neognathae was not monophyletic, and Pelagornithidae and *Gastornis* were sister to Neornithes, not Anseriformes. I did not have the time to re-run the Bayesian analysis, but I note that there are no time calibrations in the Bayes commands, contrary to the stated methods.

I reran the matrix in PAUP again and the results are the same as presented in the manuscript. I am happy to include screen recordings and logs to show this if necessary. Only Ichthyornis and extant Tinamous were designated as outgroups. The results that the reviewer is talking about only occur when *Wilaru* is included, in which case a small molecular backbone constraint for

Galloanseres needs to be used due to the extremely fragmentary nature of this fossil. This was explained in the manuscript in the "Phylogenetic Analyses" section. We did not state that time calibrations were present in the methods.

To state in the response to my review (#2) that "this specimen is one of the best-preserved Paleogene avian specimens in the world" is beyond the pale. *Paakniwatavis* is not particularly well-preserved, even in comparison to other slab-preserved Green River birds cited by the authors themselves on lines 196-201, let alone an abundance of others from the Fur, London Clay, Willwood, Quercy, Geiseltal, etc. Apparently, much of the anatomical character description and coding was performed based on CT scans of structures that are so flattened or overlain that they are not visible from the surface.

Given that only a handful of Paleogene anseriform/anseriform-like specimens are present in the world, we feel that this statement is justified. This response was given to the referee to justify the importance of the material.

For whatever reason, I did not previously have access to CT images in supposedly-linked repositories. I did not, and still do not, have access to the cover letter in which the authors claim to have provided the links. I have the active links now only because the authors included them in their response to reviewers. Now that I have been given access to them, I discount all character descriptions and scoring said to be derived from the CT imaging. The Morphobank accession includes image files of only the quadrate and tarsometatarsus. These and the CT images of the skull, phalanges, and furcula in Fig. 3 show nothing more than what can be discerned from the surface photos. (See my comments on the Dryad accession below.) This has implications for my acceptance of the character description and scoring, and the phylogenetic analysis and the downstream ancestral state reconstruction that is dependent upon it.
Still, I doubt I have access to all the images that the authors believe I should have. The entire list of files available to me on Morphobank are:
725ScanSheet.pdf
Anser_Log_Nov2_2022.txt
BayesAnser_Log_Nov2_2022.txt
done.txt
FINAL_Jun30_2022_FinalAnserMorph_withAncestralTraits.nex
FinalQuadrate.smooth.obj
FULLJuly22_withEB_allAnser.nex
fullwWilaru.te.ckp
fullwWilaru.te.ckp%7E
fullwWilaru.te.con.tre
fullwWilaru.te.lstat
fullwWilaru.te.mcmc
fullwWilaru.te.pstat
fullwWilaru.te.run1.p
Hinic-Frog-2009data.xlsx
noWilaruJuly22_withEB_allAnser.nex
numeric_Hinic_Frog_2009data.xlsx
onlynumeric_Hinic_Frog_2009data_copy.xlsx
PCA_Grace.Rmd
supplemental_figures-01.jpg
supplemental_figures-02.jpg
supplemental_figures-03.jpg

tmt.stl
M822138_Screen Shot 2021-08-04 at 2.24.14 PM.png
The first non-presbyornithid anseriform from the Eocene Green River Formation elucidates avian origins._sdd.xml
(Note: It is not so much of interest how many generations were run in the Bayesian analysis as is how many chains were run and how convergence was determined. Although the Bayes input and output files named above provide the necessary information to figure this out, this information should be presented in the Materials and Methods section.)

It certainly is frustrating that these links were not supplied by the reviewers when I first included them in the submission. I did everything in my power to make sure that the reviewers had access to the data. During the first round of revisions, I again made sure to include the links to the data in each response letter.

Inclusion of the CT data in description and scorings is necessary to understand and phylogenetically place this extinct taxon as several elements were only able to be examined and scored both by looking at the CT slices and through segmentation of skeletal elements, many of which were not visible on the surface. For example, it is not possible to see the quadrate on the surface because a carpometacarpus has fallen on top of the posterior portion of the skull and both quadrates. The CT data has been thoroughly illustrated in the slices and stl files, and we made sure to denote within the manuscript whether an anatomical feature and/or character scoring was only visible under the surface of the slab (within the CT data).

The Dryad link provided along with the revision includes only a README and supposedly the CT stacks, although there are far too few files included to be the actual CT stacks. The images are not labeled or identified in the README, but the latter seems to suggest that the authors left it to readers to segment the images (README: "This data comprises a tiff stack of the 2 part multiscan of PA 725 … We recommend use of imageJ for visualizing slices and Aviso for segmentation."). I do not doubt that the authors segmented the images because they reproduce an image of the mandibular articular region in their response to Reviewer 1. However, every image file in the Dryad download is blank. I have tried in vain to view these using a variety of image software (ImageJ, ORS Dragonfly, MeshLab, Photoshop, GIMP, MS Office), both with and without first converting from tiff to jpeg format. Though I can't be certain the problem is not on my end, I don't have any problem viewing segmentation files of my own or from elsewhere. Whatever the cause, I cannot verify the character descriptions and scoring because I can't see the structures. This applies pervasively to the authors' responses to my review. The authors might consider exporting their images as .stl files.

All CT tiffs were previously uploaded to DRYAD before the last revision deadline. The segmentation files (stl files) have been available on Morphobank (not DRYAD) since submission of the manuscript, as has been previously stated.

My reference to Livezey and Zusi's (2007) data and phylogeny in my original review was not misdirected; it was relevant to first make the point that the magnitude of the dataset might not be representative of what can be scored for any given taxon, in this case 73% of characters unscorable in *Paakniwatavis*, and second that the magnitude of a dataset does not ensure that it will produce a more accurate phylogeny. Livezey and Zusi's (2007) magnum opus is the quintessential exemplar of these points. The likelihood that morphological characters are functionally or developmentally non-independent (e.g., Tapadia et al 2005; Jheon & Schneider 2009; Bailleul et al 2017; Zelenkov & Stidham 2018 for examples in the jaw apparatus of

Anseriformes) increases with character set size. It is not credible that this crushed specimen can be scored for more than 700 functionally independent characters.

We agree with the reviewer's point that the efficacy and pitfalls of large morphological datasets is something that remains to be explored and is necessary to better understand avian evolution.

The fossil specimen was only able to be scored for approximately 30% of the characters within the matrix, and this has been shown within the matrix itself. Uncertainties regarding certain areas of the fossil due to missing data and taphonomy including crushing) have been discussed within the manuscript. We did not score any features into the matrix that we were unsure of. Extinct taxa must be placed within a matrix that contains other extinct taxa and living taxa, and this is where the larger number of characters comes in.

My original comment that the authors avoided citing relevant literature was predicated, not on what is known only from locality H, but what is known from the Green River Formation and rediagnosis of fossils described by Martin and Feduccia (1976), whom they do cite. What is the point of listing records that are now known to be incorrect? This much is not worth pursuing further. The revised manuscript is notably missing additional references that have become available in the long interim since the original submission was reviewed and the revision submitted, and that could change the outcome of the phylogenetic analysis. These include Benito et al (2022), with reference to Pelagornithidae, Gastornithidae, and Dromornithidae, as well as Mayr (2022) and Houde et al (2023) with reference to these and additional relevant early Paleogene Anseriformes with narrow bills. The authors are dismissive of Reviewer 1's attention to fossils that have been informally referred to by some as screamer-like because of their narrow bills. Musser and Clarke's excuse is that they "have already included *Chaunoides*" (a Neogene stem-screamer with 85% of characters unscored in data matrix) and that the reviewer's suggestion is "biased as to the phylogenetic position of the fossil." Hardly so. If Musser and Clarke profess to characterize macroevolution of Anseriformes sensu lato, then all early Paleogene Anseriformes are relevant - especially those that are among the most well-preserved and similar in gestalt to the clade (i.e., Anhimidae) that has long been considered primitive among Anseriformes based on outgroup comparison, and in fact most similar in gestalt to *Paakniwatavis*. Thus, it is ironic that on the one hand Musser and Clarke refer to Pelagornithidae as narrow-billed Anseriformes (their once-suggested anseriform affinities haven't been corroborated by recent analyses, and their maxilla is expansive even if long) while on the other hand rationalize their omission of unquestionable Anseriformes with narrow bills.

We thank the referee for the additional references and have thoroughly reviewed them.

We agree that *Janavis* (Benito et al. 2022) should be included in future datasets and reevaluated in the context of the Pelagornithidae, Gastornithidae, and Dromornithidae, and have now noted this in the manuscript; however, reevaluating these groups is outside the scope of this current dataset. This dataset is focused on placing *Paakniwatavis*, and due to this we only included one member of the Gastornithidae and two members of Pelagornithidae in the matrix. In future studies we would be keen to include more members of Pelagornithidae, Gastornithidae, and Dromornithidae as well as additional members of Palaeognathae to reassess these groups in the context of *Janavis*. Additional Ornithurines would also need to be added to the matrix, as it is a Cretaceous Ornithurine that was found to be outside of crown Aves in Benito et al. (2022).

We also agree that discussion of *Perplexicervix*, *Danielsavis* and *Anachronornis* within the manuscript is also important. We have since included these new anseriform taxa within the morphological matrix and rerun the analyses; however, these taxa did not change the position of any included fossils. Due to this we have added discussion regarding the new fossils and these new results throughout the manuscript but have only included trees and the matrix that includes these taxa within the supplement on Morphobank.

I pointed out that *Conflicto* and *Anatalavis* (i.e., *A. rex*) are Paleocene, not Cretaceous, and that the morphology of the bill of *Anatalavis* could not be inferred because the genus is known only from limb bones. The authors responded that Olson (1999) showed that "*Anatalavis*" had a spatulate bill, and to this they appended fig. 1 from Olson (1999) as documentation. The problem is, the Eocene *Nettapterornis* ("*Anatalavis*" sensu Olson) *oxfordi* is not referrable to the genus *Anatalavis* (type, *A. rex*). Even Olson treated the limb bones of *A. rex* as belonging to Charadriiformes. This introduced an error in the time-calibrated phylogenetic analysis. "Known Paleogene Anseriformes" do not all "have a wide, duck-like beak". Mayr (2022) and Houde et al (2023), neither of whom are cited, illustrate specimens with narrow screamer-like bills. The bill of *Conflicto* is sufficiently well-preserved to be assured that it is narrow, contrary to Musser and Clarke's description of it in their response to Reviewer 1. Furthermore, among extant Anatidae, *Mergus* has a considerably longer and narrower bill than even *Paakniwatavis*, which I only mention to make the point that we shouldn't make too much of this trait in a single fossil anseriform of otherwise uncertain affinity. The response that "[t]here are additionally no extant or extinct examples of a bird that has a narrow premaxilla and maxilla with a bill that was wider than the bilateral width of the paroccipital processes" is a tautology that makes no sense. The bill is composed of the premaxilla and maxilla. How could a bill be both wide and narrow at the same time?

We apologize for the confusion we have caused by using the name *Anatalavis*. We have now changed all instances of *Anatalavis* to *Nettapterornis*, as we only examined the *Nettapterornis* specimen for this study and did not include the *Anatalavis rex* material. We have now also included comparisons of the new possible anseriform taxa with narrow beaks from Houde et al. (2023) and rewritten our descriptions of the bill of the new taxon.

The most egregious deficiency is that there is no artist's 3-dimensional reconstruction of the skull except for an undocumented fanciful rendition in Fig 5 (below). It is ironic that the authors make detailed inferences about the dimensions of the utterly flattened skull of *Paakniwatavis* without producing a cranial reconstruction. They even say, "Due to the flattened nature of the specimen and the inability of a micro-CT scan (performed by the Field Museum via the University of Chicago) to provide additional detail beyond what it already provided regarding subsurface structures, it is impossible to reconstruct the skull in three dimensions." If so, then how can they make claims about the breadth of the supraorbital (315; vs prefrontal, my comment) or of the maxilla vs paroccipital (221, 793, 797)? On line 208 the retroarticular is described as thick, on 329 it is slender, and 701 it is exceptionally tapered throughout. I can't make it out at all in Fig. 3 or in the images the authors submitted in response to Reviewer 1. There is no evidence for the preservation of the long zygomatic, orbital process of the quadrate, unfused lacrimal, and greatly exaggerated retroarticular process as they are depicted in Fig 5. The authors have not responded satisfactorily to my original comments about Fig. 5. They illustrated the costae and sternum like those of a flightless 'ratite'. This is not "what this specimen would have looked like in life."

Due to crushing it is unfortunately impossible to reconstruct the skeleton in three dimensions. The reconstruction graphic was the closest that we could create and was just meant to give a general idea of what the bird would look like in life using the proportions and characters that we were able to from the holotype fossil. The reconstruction is not something that data was collected from and is not meant to most accurately represent the data.

We were only able to locate the width of the paraoccipital processes relative to the width of the maxilla due to the direction of crushing and the aid of CT visualization. We state that a well projected orbital crest is present but do not make assumptions regarding its breadth.

We apologize for the confusion about the retroarticular process and have removed the erroneous statement of it being “thick.”

593-594: “Within the combined results, addition of *Wilaru* further defines the base of included Galliformes recovered in the morphological analyses” Isn’t the combined analysis that of the morphological and DNA data (“combined”)? That’s what it says on line 520. If so, then what is meant by “within combined results…recovered in the morphological analyses”?
I have a few comments on specific characters.

The “morphological results” denote the results that were acquired from parsimony analysis of the morphological data, whereas the “combined results” comprise results of Bayesian analysis of the combined morphological and DNA data. We have now added this clause to the beginning of the results section.

The statements that the length of pedal digit I was determined by ancestral state reconstruction and “confirmed through scoring” (line 716) is simply confusing.

We have now rewritten this line as follows: Ancestral state reconstruction for behavioral characters using the combined results phylogeny suggests that *P. grandei* likely preferred an aquatic or semi-aquatic environment, was either not specialized for aquatic feeding modes (hereafter referred to as a “non-swimmer”) or was a surface swimmer, was primarily herbivorous, was a grazer, had some form of rhamphothecal lamellae, and that the length of itshad a pedal digit III that was subequal to or longer than that of the tarsometatarsus (character state confirmed through scoring), had an ossified pessulus of the syrinx, and did not have asymmetry at the tracheobronchial juncture of the syrinx.

My question in reference to line 229 (of original manuscript, line 260 of revision) regarded the “mandibular process”, not the “mandibular ventral angle” (line 227 of original manuscript). The authors have not addressed this.

We have now deleted this statement as it was a redundant statement that was originally meant to describe the retroarticular process.

I stand by my original comment about the lack of documentation of a ‘hooked’ hypotarsus. On the other hand, I misread “The overall shape and size of the cranium [of *Paakniwatavis*] is most similar to those of Anhimidae or Anseranatidae” as implying similarity of anhimids and *Anseranas*. I retract my comment.

We thank the Reviewer for their thorough comments; however, we believe that the line drawings, CT scan slices and segmented stl files of the tarsometatarsus provide clear documentation of a hooked hypotarsus.

Contrary to the authors' response, there are indeed two references to the deltopectoral crest, which I identified by line number. The former claims that the crest of *Paakniwatavis* differs from that of *Presbyornis* and the latter claims that it is like Anseriformes, which presumably includes *Presbyornis*. Hence, these are "a little contradictory."

Here are the two sentences regarding the deltopectoral crest—we have now changed the wording of the second sentence to include "most" Anseriformes.

"*Paakniwatavis* has a narrower crista deltopectoralis of the humerus than *Presbyornis*.

The crista deltopectoralis is prominent, rounded and flares cranio-laterally like those of most Anseriformes."

302-306-onward: The authors stress similarity to *Anseranas* and downplay similarity to anhimids (noted by Reviewer 1, with whom I agree). For example, they state that the bill is not strongly decurved as in anhimids, while acknowledging that the rostral tip of the premaxilla is also decurved in *Anseranas*, although not as strongly. Perhaps so, but this is but one feature among many. In *Anseranas*, the dorsoventral height of the maxilla and its caudal angle (i.e., the extension of the tomium ventral and caudal to the jugal process) is expansive like that of typical anatids, particularly the Anserini. Neither of these bear even the most remote resemblance to *Paakniwatavis*, which instead resembles screamers. The authors accuse Reviewer 1 of "bias about the affinities of the fossil specimen" but this can be as much an issue in the description and scoring of characters used in any phylogenetic analysis, too.

Further comparison between the new taxon and Anhimidae was added during the first round of review. Reviewer 1 has since retracted their comments as they were not relevant to the phylogenetic placement of the specimen.

If there is truly any asymmetry at all in the syrinx of *Vegavis*, then it would be necessary to make a comparison of right and left sides of some other bone(s) to exclude diagenesis as its cause. Incidentally, tracheal rings are skeletal tissue, whether ossified or not. They are ossified in some birds, including Anseriformes, and not in others.

The asymmetry we are referencing is that of ring fusion in III and cannot be mistaken to be diagenesis. Please see the 3D reconstruction of the *Vegavis* syrinx below from Clarke et al (2016) Supplemental Data.

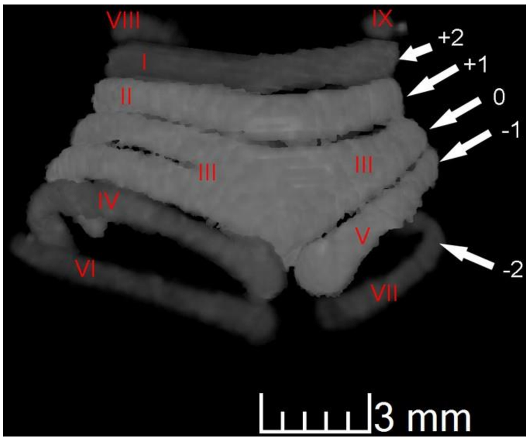

Next, I address the ancestral state reconstruction.
The unfortunate muddling of the description of the new fossil, *Paakniwatavis*, and of a new dataset used both for phylogenetic analysis of the new fossil and for ancestral state reconstruction of PanAnseriformes results in confusion as to their distinction. This clearly disturbed Reviewer 1 when s/he complained that the data matrix included even non-osteological characters that were unscorable in *Paakniwatavis*, and for which it could contribute no relevant data. This is an issue for me, too. I understand that ancestral state reconstructions apply to every internode on a tree regardless of whether data exists for it. Technically speaking, one could infer anything whatsoever about unknown attributes of extinct taxa, while knowing nothing other than the distribution of those attributes in living forms. Still, the presentation seems a bit disingenuous in that it seems to imply that the new fossil is somehow contributing to the knowledge of the distribution of characters such as rhampothecal lamellae, palmation, foraging behavior, etc., when in fact it does not at all. Ideally, this should be made clearer.

We have made this clear throughout the manuscript, especially in the Discussion section. We agree that additional fossils added to the dataset in the future will aid in providing a better understanding of ancestral state reconstructions for this group.

506: "Ten additional characters were added to the morphological matrix for ancestral state reconstruction of relevant traits … using parsimony methods." In seeming contradiction, it is stated (lines 48, 117) that "We additionally combine the new morphological matrix with molecular data for Bayesian analysis and perform ancestral state reconstruction…" If the authors do not intend to say that Bayesian analysis was used for ancestral state reconstruction, then they should rewrite this sentence. The characters targeted for ancestral state reconstruction may be "relevant" to anseriform evolution in general, but this fossil has no bearing on them. I question their transitions as "definitive" in the caption to figure 5. As noted by the authors, ancestral state reconstruction is dependent on correct placement of fossil taxa in a phylogeny. Thus, in light of the significant disagreements on the positions of most Paleogene or older Anseriformes expressed in the literature (e.g., see review of *Vegavis* in Acosta Hospitaleche & Worthy 2021), little confidence can be placed in the ancestral states advocated here. To echo Reviewer 1, "Given the unstable position of the fossil (and other anseriforms) in your analyses, ancestral state reconstructions are of rather limited value."

We have already made this clear in the manuscript as follows: "Ten additional characters were added to the morphological matrix for ancestral state reconstruction of relevant traits that detail habitat preference, swimming mode, diet, status of rhamphothecal lamellae, status of pedal webbing, feeding mode, syrinx anatomy, and the relative length of pedal phalanx III compared

to that of the tarsometatarsus. Ancestral state reconstructions were performed in Mesquite [78] using parsimony methods."

The position of the newly described fossil did not change across any analyses. Although the ancestral states do hold value, especially as this is a large foundational dataset that will be critical for use by future workers; however, we agree with the Reviewer that the states are likely to change once new taxa are added and have already noted this in the conclusion of the manuscript:
"Further analysis of these Paleogene anseriform fossils in the context of broader extinct taxon sampling, especially in the context of extinct anseriform-like taxa, is necessary to further evidence placement of *P. grandei* and other Paleogene anseriform-like taxa and to gain more robust insight into ancestral states; however, *P. grandei* represents a key taxon, a unique ecology within known Anseriformes and the Green River Formation, and a potential calibration point for anseranatids. Other included extinct taxa also represent potential calibration points that would be valuable for stem Anseriformes, Anhimidae and stem Anatidae, although recovery of additional fossils and further phylogenetic analyses (especially of taxa such as *Chaunoides* and *Wilaru*) are preferable to confirm these relationships, further reveal the ecological and behavioral evolution and biogeography of Anseriformes, and better elucidate our understanding of avian evolution."

I strained my eyes at length to read the legend of ancestral states in Fig 5 but the image quality is so poor that I could not even guess several. I had to refer to the data matrix to decipher the taxa in the phylogeny of Fig 5. Regardless, in disagreement with the results shown, it is clear from the ladderized phylogeny of outgroups, anhimids, *Paakniwatavis*, *Anseranas*, and Anatidae, resp., that the narrow screamer- and galliform-like narrow bill is primitive among Anseriformes, that the palatal ridges of screamers are not vestigial rhampothecal lamellae but preadaptations at most (even some parrots have well-developed ridges), and that none other than Anseres evolved even semipalmation. I can't read the remaining states.

We respect the Referee's opinion on these characters; however, this is the optimization that was recovered with our analyses, and the referee disagrees with results recovered. If the Referee has their own hypothesis considering this trait we welcome their perspective, but these are the results we garnered with the data at hand. We uploaded full sized figures during the submission process, and do not have control over whether these were downsampled by the Journal.

Problems with other analyses follow.

We would like clarify that in some cases the Referee is taking issue with the results of the analyses, not the analyses themselves.

I acknowledge that the authors rounded their body mass estimates to the nearest whole integer in their revision, but confidence intervals need to be reported. This is particularly relevant in as much as fossils will present greater estimation uncertainty than modern bones on which the published regressions were based, and because *Presbyornis* exhibits significant size polymorphism.

We have reached out to the author of the regression equations in order to obtain the necessary data to calculate the confidence intervals. If confidence intervals cannot be calculated, we are happy to remove the body mass estimates from the manuscript as they are not necessary.

I do not doubt that femoral length measurements provided in Table 2 are faithfully precise, but the 8-10% differences between right and left measurements of coracoids and ulnae underscore the uncertainty of measurement accuracy of this thoroughly flattened specimen. (Incidentally, Table 2: right and left measurements are distinguished by a forward slash for *Paakniwatavis*, but not the other taxa. What is the significance of the comma for other taxa?)

The commas have been removed and replaced with hyphens as they were meant to denote a range. We agree that crushing creates a small amount of uncertainty regarding measurement accuracy; we have done the best that we can with the state of the specimen that we have.

Moreover, a strong case can be made that the regression for Anseriformes proposed by Field et al (2013) is not equally applicable to Anhimidae, typical Anatidae, and the stilt-like *Presbyornis*. Navalón et al (2022) should be consulted for a treatment of limb morphospace across avian clades.
We understand the Referee's opinion regarding the utility of this regression; however, there is no other regression available for specific subclades.

The PCA and LDA analyses should be discarded altogether since they produced demonstrably spurious results. Neither *Chauna* nor "*Anatalavis*" could be wing-propelled divers since they do not exhibit dorsoventral diaphyseal compression of the humerus that is present in all extant wing-propelled divers. More convincingly, as living species, *Chauna* spp. are known not to be. "*Anatalavis*" exhibits no cranial adaptation for either high or low altitude plunge diving, and to the best of my knowledge this behavior does not exist in any Anseriformes. The authors concede that "the rest of the results are largely inconsistent with other features or the known behaviors." So why include this?

We agree with the Referee and have always noted so in the Methods section of the main paper. This is why we did not include these analyses in the main body of the paper. We include them in the Supplementary Data in case they can have future utility since they are published datasets.

My penultimate comments refer to numerous unrelated, unsatisfactorily resolved issues. *Diatryma* Cope, 1876 is a junior synonym of *Gastornis* Hébert, 1855, with which it has been repeatedly synonymized (Andors 1992; Mlíkovský 2002; Angst 2014). It is unfathomable how the authors could assert otherwise. Use of the name *Diatryma* is unheard of in recent literature. Also, "*Anatalavis*" *oxfordi* Olson, 1999 was reassigned as *Nettapterornis oxfordi* Mlíkovský, 2002 (Mlíkovský 2002) and the latter name should be used to avoid attributing duck-like attributes to older fragmentary fossils of uncertain affinities.

We apologize for both errors and have now changed all instances of *Diatryma* to *Gastornis* and all instances of *Anatalavis* to *Nettapterornis*.

Olson and Feduccia (1980) did not "propose" that pedal webbing "is present in extant Anhimidae". On the contrary, the passage to which Musser and Clarke refer is in reference to *Anseranas*. Olson and Feduccia inferred that the ancestors of screamers "must have passed [through the semipalmate state] in becoming increasingly terrestrial" based on their unfounded assumption that screamers evolved from typical palmate ducks. In other words, this was an inference about ancestral character states that was neither informed by direct observation nor by formal analysis. It is relevant to note that Olson and Feduccia's agenda was to discredit a sister relationship of Galliformes and Anseriformes in favor of the derivation of Anseriformes from

shorebirds. The monophyly of Galloanseres is now as well-proven as any phylogeny can be, and Charadriiformes are members of their sister clade Neoaves.

In the first paragraph of the Introduction in Olson and Feduccia (1980), they write:

"The three species and two genera of Anhimidae are large birds with short, somewhat fowl-like bills and rather long legs, with long toes having vestigial webs; considerable morphological differences exist between the two genera, Anhima and Chauna (Beddard and Mitchell, 1894)."

A conjecture scribbled on a specimen tag ("?Heliornithidae") is neither a referral nor is it worthy of note to the exclusion of any reference to actual publications in which this very specimen was referred to as a probable or actual anseriform with similarity to screamers (Grande 2013; Mayr 2022; Houde et al 2023). The authors are certainly aware of Grande (2013) among these at the very least since it is cited (without reference to Anseriformes) in the same paragraph. I am left to wonder why this is obscured.

One of the authors aided in providing ID's for Grande 2013. We are happy to cut this from the manuscript and have done so.

If the authors claim that *Wilaru* "is not well supported as a member of Presbyornithidae" and that "there are many conflicting results for its placement across previous publications" then they should cite those studies. In a reversal of her own 2013 paper, De Pietri (De Pietri et al 2016), whom Musser and Clarke do cite, in fact specifically advocate its position in Presbyornithidae. Description of Wilaru specimens as highly fragmentary (603) is a gross mis-characterization as I stated in my original review, unworthy of debate. Unlike *Paakniwatavis*, its entire appendicular skeleton is known from numerous beautifully undistorted 3-dimensionally preserved specimens. More to the point, every Cretaceous or early Paleogene fossil that has been referred to Anseriformes, without exception and including all those in the present study, has been described as a member of one or more other orders. So, this is no justification to discount *Wilaru*.

We agree with the Reviewer and have now rewritten the sentences in question as follows: "These drastically different placements across the combined and morphological data analyses are likely due to the lack of a skull known for *Wilaru*, although it does suggest that *Wilaru* may not be a presbyornithid as previously hypothesized in De Pietri et al. 2016. Due to this, we will largely focus on analyses that exclude *Wilaru*.

A trivial point, but I recommend restructuring the sentences including "un/ambiguous optimized" so that these terms do not appear to be adverbs describing optimization.

We appreciate this point but are unsure of how to restructure the sentences in a more clear manner.

There are minor editorial/punctuation/formatting/typo errors scattered throughout e.g., but not exclusively, lines 150, 166, 168, 169, 561, 567, 610, 720, 809, 903.

We thank the Referee for pointing these out. These have now been fixed.

I think the section "Nomenclatural Acts" will be confusing to readers and should be removed as long as the editors of PLoS ONE, Zoobank, and Commissioners of the ICZN are satisfied that the Rules of Zoological Nomenclature have been followed. I am unapologetic for consulting Zoobank about nomenclatural availability. As stated in my original review, well before the current manuscript was submitted anywhere, I had consulted the Commissioners of ICZN as to whether I should or should not identify a new taxon of my own by name in a bioRxiv preprint. I was advised against doing so. Thus, I had reason to believe there might be a problem here. I queried Zoobank about the LSID that appeared in bioRxiv before Musser and Clarke's manuscript was even submitted to PloS ONE. The response I received and forwarded in my review indicated that this was an interesting case that deserved to be addressed by the Commission (and that the only other single case of an LSID in bioRxiv was published). I would not have done due diligence as a reviewer if I did not report this in my review. Except for the LSID, I did not disclose any part or whole of Musser and Clarke's manuscript to anyone, but the manuscript could hardly be considered confidential since it was already universally distributed in its entirety via internet on bioRxiv and discussed in online blogs.

We will defer to the editor as to whether the Nomenclatural Acts section should be removed.

In summary, the Discussion is excessively verbose and rambling, particularly with regard to ancestral states. The reconstruction in Figure 5 is grossly incorrect and misleading and needs to be remade or discarded. The meshes of the quadrate and tarsometatarsus in the Supplemental files are unworthy of publication except to raise doubts about other inferences made solely on the basis of the CT scans. There is no artist's 3-dimensional reconstruction of the skull. Recently published relevant works are not incorporated or even cited. The results are overinterpreted and their significance to the understanding of anseriform evolution is grossly overstated. Minor editorial adjustments aside, the authors appear to be unwilling to consider the merits of issues of substance suggested by reviewers.

Please see above responses. Again, the reconstruction was made with faithful proportions to give an idea of what *P. grandei* would have looked like and is not meant to be an anatomical figure that should be used for scoring. This dataset and our results, including the ancestral states, provide an important foundation for the phylogeny of Anseriformes that will be added to and changed by future workers. Exploration of the base ancestral states is necessary to provide a foundational dataset from which future work will build off of, including the ancestral states themselves.

My last point is just a bit of advice: accusing reviewers of being biased, unprofessional, unethical and addressing them in all caps is probably not the most effective way to sway their opinion.

We did not accuse the Reviewers of being unprofessional or unethical. We only used all caps when calling attention to the repositories where the data could be viewed.

We thank the Referee for the suggested citations below and have noted which ones were added to the manuscript.

Acosta Hospitaleche & Worthy 2021. New data on the *Vegavis iaai* holotype from the Maastrichtian of Antarctica. Cret. Res. 124, 104818—This citation has now been added.

Andors 1992. Reappraisal of the Eocene groundbird *Diatryma* (Aves: Anserimorphae). Nat. His. Mus. Los Angeles Sci. Ser. 36, 109–125—This was already cited in the manuscript.

Angst et al 2014. Isotopic and anatomical evidence of an herbivorous diet in the early Tertiary giant bird *Gastornis*. Implications for the structure of Paleocene terrestrial ecosystems. Naturwissenschaften 101, 313–322—This citation has now been added.

Bailleul et al 2017. Cranial joint histology in the mallard duck (*Anas platyrhynchos*): new insights on avian cranial kinesis. J. Anat. 230, 444-460

Benito et al 2022. Cretaceous ornithurine supports a neognathous crown bird ancestor. Nature 612, doi.org/10.1038/s41586-022-05445-y—This citation has now been added.

Field et al 2013. Skeletal correlates for body mass estimation in modern and fossil flying birds. PLoS ONE 8(11), e82000 —This was already cited in the manuscript.

Jheon & Schneider 2009. The cells that fill the bill: neural crest and the evolution of craniofacial development. J. Dent. Res. 88, 12-21

Livezey & Zusi 2007. Higher-order phylogeny of modern birds. Zool. J. of the Linn. Soc. 149, 1-95 —The 2006 portion of this was already cited in the manuscript; the 2007 portion is not discussed in this study.

Mayr 2022. Paleogene Fossil Birds, 2nd Ed. Springer, ISBN 978-3-030-87644-9—This citation has now been added.

Mlíkovský 2002. Cenozoic Birds of the World, Part 1: Europe. Ninox Press—This citation has now been added.

Navalón et al 2022. Environmental signal in the evolutionary diversification of bird skeletons. Nature 611, doi.org/10.1038/s41586-022-05372-y

Olson 1999. The anseriform relationships of *Anatalavis* Olson and Parris (Anseranatidae), with a new species from the lower Eocene London Clay. Smithson. Contrib. Zool. 89, 231–243—This was already cited in the manuscript.

Olson and Feduccia 1980. *Presbyornis* and the origin of the Anseriformes (Aves: Charadriomorphae). Smithson. Contrib. Zool. 323, 1–24—This was already cited in the manuscript.

Tapadia et al 2005. It's all in your head: new insights into craniofacial development and deformation. J. Anat. 207, 461-477

Worthy et al 2017. The evolution of giant flightless birds and novel phylogenetic relationships for extinct fowl (Aves, Galloanseres). R. Soc. Open Sci. 4, 170975—This was already cited in the manuscript.

Zelenkov & Stidham 2018. Possible filter-feeding in the exinct *Presbyornis* and the evolution of Anseriformes (Aves). Zool. Z. 97, 943-956
