# Supplementary material for: A new Paleogene fossil and a new dataset for waterfowl (Aves: Anseriformes) clarify phylogeny, ecological evolution, and avian evolution at the K-Pg Boundary
Source: PLoS One. 2024 Jul 30;19(7):e0278737. doi: 10.1371/journal.pone.0278737 (PMC11288464; doi:10.1371/journal.pone.0278737)
Supplement: Attachment — Submitted filename: 2_20_2024_Response_to_Reviewers.pdf [file pone.0278737.s012.pdf]

Reviewer #1: (1) I strongly suggest to restructure the Differential Diagnosis and to better delimit the new taxon from Danielsavis and Anachronornis.
You start by saying "The rostrum shape exhibited by this taxon is unlike most other previously recovered
Paleogene Anseriformes". However, then you rightly proceed by saying that "When compared to extinct taxa, the narrow bill of Paakniwatavis is most like those of Anachronornis anhimops and Danielsavis nazensis". Most of the comments pertaining to Danielsavis and Anachronornis in your Diff Diagnosis refer to the fact that comparisons of certain elements are not possible, and what would be needed is the inclusion of two paragraphs, in which the new taxon is clearly differentiated from Danielsavis and Anachronornis (note that the wider coracoid shaft may be the result of flattening of the GRF specimen). Actually, a differentiation from Danielsavis and Anachronornis is key to your manuscript, since these two taxa certainly are most similar to your new fossil.

We have now included these paragraphs and a revised differential diagnosis as requested by the Reviewer. Even with diagenesis considered, we still believe that the coracoid is wider and more robust in *Paakniwatavis* compared to *Danielsavis*.

In a differential diagnosis you should actually list similar taxa and differentiate the fossil from the by saying ABC differs from XYZ in .... A diagnosis, by contrast list characters that are considered distinctive (ideally autapomorphic) for a new taxon. Your diagnosis is confusing, because you mix both approaches and make comparisons by skeletal elements, not by taxa. Instead of writing feature XYZ is like in ABC but different from DEF, it would be much clearer to structure the Differential Diagnosis by differentiating Paakniwatavis from each taxon separately (mainly Anachronornis, Danielsavis, and Nettapterornis)

We have now revised the differential diagnosis to match this suggested format.

You use the bill width of the new taxon in the differential diagnosis and say that "Paakniwatavis grandei exhibits a mediolaterally narrow bill that is narrower than the width of the skull at the parocciptal processes". However, this statement is not quite clear and needs to be specified further. On the one hand, the skull is exposed in lateral view, so I do not quite understand how you can infer bill width. On the other hand, from what is preserved, the beak appears to have been tapering towards its tip, so that that its width was not constant (the base certainly was wider than the tip).

Thank you for this clarification—we agree with the Reviewer's assessment of the bill but our wording was not as clear as it could have been, and we have now revised the wording to make this more clear. We are asserting here that 1) the base was wider than the tip, and 2) that the base of the bill is narrower than the width of the skull when measured from the left to right paroccipital process. We can only tell this via the CT scan-even with crushing, the width between paroccipital processes in wider. In most extant Anseriformes we have found the opposite to be true-that the base of the bill is equal in width or wider than the width between the paroccipital processes.

As a side note: I do not think that the Anseranas-like skull of your reconstruction matches the preserved outline of the skull of the fossil, in which the beak appears to be more anhimid-like. In line 102, you note that "the new fossil presents a narrow bill that is most similar to the Anhimidae" - why, then, has the beak be reconstructed as Anseranas-like (dorsoventrally narrow, hooked tip etc.)?
It is also a misleading that your reconstruction shows a crest or feather tuft on top of the skull - I see no evidence fort this in the fossil, which does not exhibit any soft tissue preservation. This crest/tuft serves to make the reconstruction more Anseranas-like than what is supported by the actual fossil, which likewise shows no evidence for the length of the tail feathers.

The artist's rendition has now been removed.

(2) Please also note that the Danielsavis material has meanwhile been redescribed and additional features (such as the palate and the pedal phalanges) having been described. Danielsavis was found to be very different from Anachronornis and may actually be a stem group galliform not an anseriform; it is now assigned to a new taxon Danielsavidae.
Mayr, G., Carrió, V., & Kitchener, A.C. 2023: On the "screamer-like" birds from the British London Clay: An archaic anseriform-galliform mosaic and a non-galloanserine "barb-necked" species of Perplexicervix. Palaeontologia Electronica 26(2):a3328; doi: 10.26879/1301.

We thank the Referee for this note and have now included this new paper in the revised description—this allowed for much better differentiation between the other new taxa and our fossil.

I agree with the comments of the other reviewer that it would be appropriate to note that Grande (2013) compared the fossil with the Anhimidae. Even if this identification may go back to an initial identification by one of the authors of the present manuscript, it is a published statement upon which future readers may stumble.

This has now been added to the manuscript.

- line 302: I agree with reviewer 2 that a distally "hooked" hypotarsus is not visible on the published figures

We respect the Reviewers' opinions but we have been working on this fossil, observing it, segmenting it, studying the CT scans since 2019 and do not agree with them. If the Reviewers are still not convinced by the figure they may refer to the CT slices in the supplement—these were not published in the figure as it is not possible to publish a series of CT slices in a movie format for a figure.

- line 596: sp. nov. (instead of "sp. Nov.")

This has now been fixed.

- Line 827: "Anachronornis anhimops and Danielsavis nazensis were largely placed outside of Paleognathae" - I do not quite understand why this statement is here, since none ever suggested that these birds are palaeognathous. Note also that it is "Palaeognathae".

The spelling has been changed. These statements are in the manuscript to show the outcome of including these taxa in our dataset and why we did not include them in the main analyses/main figures.

Reviewer #2: Review of PONE-D-22-32093R2 A new Paleogene fossil and a new dataset for waterfowl (Aves: Anseriformes) clarify phylogeny, ecological evolution, and avian evolution at the K-Pg Boundary.

59-60: citation 11 recovers Pelagornithidae as neoavian, not anseriform, as does more recent analysis by Benito et al, cited elsewhere in the manuscript but not cited here. The disagreement among analyses is discussed much later (678-692) but is misrepresented here that there is no disagreement. It should be.

Citation 11 has now been removed from this instance.

62: Anhimidae are aquatic foragers, not terrestrial except maybe in captivity

This sentence has been reworded to avoid confusion.

73: the term vestigial explicitly refers to an organ that is derived from a functional one but that is no longer functional. This is not the same as the term rudimentary, which does not imply character state polarity. The use of term vestigial is appropriate in the context used here (because this had been suggested long ago) but inappropriate elsewhere in the manuscript.

We do not have any other instances of the word “vestigial” in the manuscript.

73-74: anhimids are not palmate, semipalmate at best. This is in fact conceded elsewhere in the manuscript.

Pedal webbing has been corrected to “semipedal webbing.”

81: either misplaced period or capitalize “and”

This has now been corrected.

103, 227: Mayr et al (2023; https://doi.org/10.26879/1301; not cited) vitiated the suggested anseriform relationship of Perplexicervix

This citation has now been added.

206 Diagnosis, 241: Unfortunately, Anachronornis is insufficiently preserved (lacks complete tibiotarsus and tarsometatarsus) to distinguish from Paakniwatavis based on these diagnostic criteria, but it is obviously a different animal based on other characters. Perhaps one or more some should be mentioned in the Diagnosis. I am not fully convinced that the crushed furcula is thick as described based on figure 3N and 3O. It could be, but it doesn't look different than that of Presbyornis in fig. 3M.

The diagnosis has now been completely revised. The furcula of *Paakniwatavis* is thicker than those of all extinct taxa that were included in the differential diagnosis, even when accounting for diagenesis.

224: add space "and3"

This has now been corrected.

230: Explain what is meant by "the facial margin" of the bill or use accepted terminology. It isn't clear to me.

This has now been changed to "tomial margin."

233: relative lengths of external nares and rostrum of Anachronornis and Danielsavis are published (Houde et al 2023 figs. 1D and 7; Mayr et al 2023 fig 1B) so this statement is incorrect

This has now been revised and corrected.

249: Houde et al specifically state the humerus of Anachronornis is apneumatic (Houde et al p 19 "Humerus: Nonpneumatic.") so this statement is incorrect

This has been revised.

254: misplaced "I"

This has been corrected.

258: do the authors mean metacarpals instead of digits? There are no synostoses in the digits (i.e., phalanges). If the carpometacarpus is intended here, then they should also indicate that this is in reference to the distal synostosis.

This has now been corrected.

281-282: “pedal phalanx IV: digit IV is longer than IV: III” makes no obvious sense because the fourth phalanx in digit III is a claw and in digit IV it is not. Perhaps the words digit and phalanx are transposed.

This has now been corrected.

283-288: the description goes from carpometacarpus to humerus back to carpometacarpus. This disorganization was commented on in a previous review.

The description has now been thoroughly revised.

318-319: no aspect of the bill even remotely resembles Anseranas except that the caudal portion (only) of the tomium is straight. This was commented on in a previous review. The authors are unwilling to concede the obvious.

The bill resembles that of Anseranas as the bill is narrow and the nares are similar length and shape.

330-331: I have been provided with no image to verify that the postorbital process was long as claimed. It would be surprising if it was long, since it is not in either anhimids or Presbyornis. This was commented on in a previous review.

The fossil is not closely related to Anhimidae or *Presbyornis*. The postorbital process can be seen in the CT slices in the supplement.

379: It is ambiguous whether the authors are stating that the vertebrae of Presbyornis, Nettapterornis and Telmabates are or are not heterocoelous.

This sentence has been revised for clarity.

381: As above. It is less ambiguous to say “The notarium is present in all Anseriformes…”

This sentence has been revised.

431: forms

This has now been corrected.

454: nix “appears”

This has now been corrected.

455: which of the two cnemial crests, cranial or lateral?

Cranial, this has now been corrected.

517: segmented files are not available on morphobank as claimed. There is one obj file of the quadrate and one stl file of a tarsometatarsus, nothing more. None of the descriptions and character scoring based on segmented CT scans other than these two are verifiable by readers. They need to be.

These are the only segmented files that we created. When we tried to segment the rest of the skeleton it was too crushed to be useful. The rest of the characters are visible in the slices that are on DRYAD—unfortunately in crushed specimens like this, segmenting does not usually offer clarity into character states as much as the CT slices do. An OBJ file is a different version of an STL file but can still be viewed as a mesh—it is still the segmented portion of the skeleton.

521-526: There are only 41 taxa included in the nex files in morphobank. In other words, the matrix provided does not include Anachronornis, Danielsavis and Perplexicervix as claimed. Just as importantly, the matrix includes no unambiguous Neoaves. The importance of this is that there are no data included to challenge the recovery of Pelagornithidae within Galloanseres, which is a viable and the most current hypothesis of their relationships.

We have now included a version of the dataset on Morphobank that includes Anachronornis, Danielsavis and Perplexicervix. We did not include Neoaves as there is still debate as to which Neoaves are the most basal and it is out of the scope of this paper—although we have plans in the future to combine this dataset with Musser’s large neoavian dataset.

571: In a previous review, objection was raised about the inclusion of the PCA and LDA analyses on the grounds that they recovered results known to be incorrect even for neotaxa. The authors dismissed this concern. They stated that they agreed and therefore moved these analyses to the Supplementary Data. Maybe so fig S4, but in fact they retained these in the Methods and in the Results section 778-794 and the results are alluded to in the Discussion in the form of interpretations. It should be deleted altogether.

The analyses were included to show that we attempted to reconstruct results for our added taxa but that it was not possible, and is very thorough in describing this and letting readers know where they can further scrutinize these in the supplement. We think that it is important to include so readers have all available information on performed analyses.

575: Nettapterornis is labeled Anatalavis in the PCA figure S4.

This has now been corrected.

582-584: the authors state in their response to reviewers that Paakniwatavis is recovered as sister to Anseranatidae plus Anatidae, but here it clearly states "Paakniwatavis … is recovered across all analyses as the sister taxon of Anseranatidae." What's more, if sister to Anseranatidae plus Anatidae, then in fig. 5 Anatidae includes Vegavis, Presbyornis, Conflicto, Telmabates, and Nettapterornis. That is going to raise some eyebrows.

This sentence has now been corrected. We understand the Referee's point about the fossil clade but no matter what configuration of the dataset was used, these are the rendered results and we have spent years double checking the characters. Another paper by Torres et al. in press has also recovered the same fossil clade using an independent dataset, thus it is not likely to be an error caused by the distribution of characters and/or taxa in our matrix.

536: delete ".;"

This has now been corrected.

537: What "iteration"? Do the authors simply mean they adopted characters from other datasets? If so, then just say so.

It is an iteration of the Musser and Cracraft (2019) dataset for all Neoaves which is still growing. This has now been further explained in the Methods section.

552 and 556 are redundant.

This is standard methodology description used in many similar phylogenetic papers, including Musser et al. 2019 [144] for example.

585: both?

Yes, there were two different phylogenetic analyses performed as described in the Methods and Results sections.

597: see comments about fig. 5

See comments below.

603-604: is somewhat redundant with 673-674 with regard to Dendrocygna and Thalassornis

No, because we are discussing two different phylogenetic analyses here.

611: fhigh

This has now been corrected.

643-645: Rewrite sentence. Did Sun et al use mitochondrial genomes or two genes? Does “which contains almost all extant anseriform taxa used in our analysis” mean that there was sequence data for all the extant species used in Musser and Clarke’s analysis? This is confusing.

This has now been partially rewritten. The Sun et al. study contain several different analyses of different datasets. One is an analysis of mitochondrial genomes, and one is an analysis of just several genes. We discuss both here.

646: delete “an”

This has now been deleted.

652, 673, 674: Thalassornis is misspelled

This has now been corrected.

678-683: run-on sentence would be better broken into two, first reporting the results and the second the explanation for the low support.

This has now been revised.

688-709: the Results section should report the results of this study, but it includes considerable text reviewing the results of other peoples’ analyses. Those ought to go into the Discussion.

We thank the Referee for the suggestion; however, since several phylogenetic analyses were conducted within this paper we felt it more appropriate to include comparisons of all results within the Results section and discuss our primary results within the Discussion Section.

696 and elsewhere: anatid should be capitalized only when expressed as the formal name Anatidae

This has now been corrected.

730: to say the crest is prominent obviates the need to say it is present

744-777: I like the two paragraphs on ancestral state reconstruction. It is candid and appropriately circumspect. Unfortunately, the same cannot be said for how these traits are mapped onto the phylogeny in figure 5 and how they might be misinterpreted by readers who do not pay sufficient attention to these two paragraphs. Please see my comments on fig 5 below.

Please see our comments below.

752-754: both some jacanids and anhimids have third digits that are longer than the tarsometatarsus (specimens directly available to me, but consult Ridgeway for individual variation). Both are renowned for walking on floating vegetation, not swimming (although screamer chicks notably swim), for which the long toes are specialized. Ref 106 is irrelevant to the authors' point since it concerns claw curvature, not digital length.

Jacanids are neoavian Charadriiformes which are highly specialized and not within the scope of this dataset. It is still not confirmed whether the long toes in Anhimidae are useful expressly for walking on floating vegetation. Here we are discussing pedal proportions including the claw and that is why we included Ref 106.

778-794: this paragraph really needs to be deleted, as does figure S3, as articulated in previous review. Even the authors concede "the results are largely inconsistent with other features or the known behaviors [of neotaxa] (i.e., Chauna)". It is nonsense, so don't include it.

We feel that it is critical to include so that other workers do not waste time replicating these analyses for future studies, as it is one of the only datasets available for PCA and LDA studies of these taxa.

795: At the risk of sounding gratuitously critical, the writing could be much improved. Much of the discussion is rambling, verbose, rhetorical, speculative, redundant, tangential or irrelevant, and conversational. It reads like a first draft. It would benefit from being shortened with an eye

to the elements of style. There are excellent books on the subject that should be read by all whose careers depend on effective communication.

We have been working on this manuscript since 2019. It is closer to a hundredth draft than a first draft. It was also approved as a thesis chapter by a committee of renowned scientists including Drs. Joel Cracraft, Julia Clarke, Christopher Bell and Melissa Kemp with minimal changes.

799-800: The relationships of Pelagornithidae and Gastornis are not tested in this study because their purported neoavian sisters were not included in the dataset. It is meaningless to report where they are recovered among taxa that do not represent the diversity of current alternate hypotheses of their relatives. There are three ways to deal with this. First, add representative Neoaves to the dataset and rerun the analyses. Most of these data are already scored in the datasets of the studies from which the current matrix was derived. This would be a valuable exercise even if some data were missing. Moreover, they can be part of a backbone constraint tree. Second, Pelagornithidae and Gastornis could be deleted from this dataset and report. Third and by far the least desirable, the authors could include a caveat that states that Pelagornithidae and Gastornis are recovered as … but their proposed neoavian sisters (include refs) were not included in this study. I realize the third option is the easiest for the authors because it requires virtually no effort, but it is the poorest remedy if a true remedy at all.

They are tested to the fullest extent possible within the scope of this paper, because Pelagornithidae are not neoavian and Gastornis has been hypothesized to be a close relative of them and/or Anseriformes. Adding neoavian taxa is outside of the scope of this paper, especially since there is still not a consensus for what the most basal group within Neoaves is. We have added the third option as suggested by the Reviewer. Even if 3-4 neoavian taxa were added, this would not be enough to test the relationships of all included taxa fully since a comprehensive dataset for Neoaves is needed. This dataset is being created by Dr. Musser and published iteratively but is out of the scope of this paper and will years of continuing to build the dataset. Redoing the dataset would mean that the analyses, data and manuscript would need to be redone 1.5 years after submission of the paper, and the Editor has agreed that this is not necessary.

826-827: the phrase "especially combined with a reduced form of rhamphothecal lamellae present in extant Anhimidae [22]" should deleted because it implies character state polarity of rhamphothecal grooves in Anseriformes that is not supported by the current analysis. In fact, that the grooves are "reduced" is contradicted by the ancestral state reconstruction. "Incipient" might be a more appropriate descriptor than reduced, but that too implies character state polarity that truly is beyond the scope of this study.

We thank the Reviewer for the suggestion, but here we are discussing a trait that is present in extant Anhimidae and our resulting ancestral state optimizations based on the scorings in our matrix. We are not discussing state polarity, only referring to the results that can be seen in the Mesquite file on Morphobank.

838-840: No, Conflicto, Anachronornis, and Danielsavis all have narrow bills and only Presbyornis and Nettapterornis are known to have wide ones. Brief reference to Anachronornis and Danielsavis is appended to the end of this paragraph (857-859) in what seems to be a minimal effort for resubmission without actual revision that brings this manuscript up to date. The bill of Conflicto is narrow and utterly unlike that of Nettapterornis. The bill of pelagornithids is so manifestly different from that of Anseriformes (instead, like those of neoavian Pelecaniformes and Procellariiformes, e.g., in the presence of rhamphothecal segmentation as well as dimensions) that pelagornithids don't warrant mention here.

The state we explicitly discuss in this paragraph that *Conflicto* has is that its beak is "mediolaterally wider than the width across the paroccipital processes," and this is consistent with the states in Anseranatidae and extant Anatidae. Also the beak of *Conflicto* is broken along the sides and front, indicating that if unbroken the bill would have been wider than those of *P. grandei*, *Anachronornis* or *Danielsavis*. We have now added *Anachronornis* and *Danielsavis* to the "narrow beak" discussion portion as suggested by the reviewer. Pelagornithidae have been shown to be closely related to Anseriformes, and thus it is our opinion that it is important to compare their beak shape to that of other Paleogene Anseriformes.

860-888: this entire paragraph is speculative story-telling and should be deleted. The argument as stated in 863-865 is flawed because Anhimidae is not nested within Anseranatidae-Anatidae (it is sister to them instead; fig 5) and because screamers are aquatic foragers, not terrestrial. The presence of the diminutive ridges in the rhamphotheca of screamers is not evidence of developed rhamphothecal grooves as an ancestral state in crown Anseriformes and that they are vestigial in screamers. Lines 875-878 are undoubtedly correct, but without requiring a character state reversal in screamers. I don't think 865-866 is correct. As stated in previous review, ridges exist in the rhamphotheca of at least some Psittaciformes (lacking pedal webbing) for what it is worth. The excessive digression on non-avian dinosaurs and turtles is tangential and a distraction. The fact is that rhamphothecal grooves have been specialized for a variety of diverse foraging behaviors in Anseriformes and others, including filter-feeding in Anatini (and flamingos), piscivory in Mergini, and grazing in Anserini. Last, 887: it is not news that "feeding ecology has acted as the primary selective force in waterfowl beak shape diversification [19]." Feeding ecology is the primary selective force in beak shape of all birds, without exception. Thermoregulation, display, and phylogenetic inertia play secondary roles in some.

We know that Amhimidae are aquatic foragers, but this is only in the sense that they forage from the bank and are largely terrestrial birds. We are not claiming that Anhimidae is nested within

Anseranatidae-Anatidae. This paragraph is a highly thought-out discussion of the ancestral state optimization results and includes what science is available.

916-918: This sentence adds nothing

We respect that the Reviewer has differing opinions but we do not agree with them.

920: Dromornithids were not included in this study, so why are they included in “this clade”?

Dromornithidae are included in the discussion here as they have amphicoelous thoracic vertebrae and are thus relevant to the discussion of thoracic vertebrae type here.

919-927: this paragraph should be reorganized with a topic sentence that the thoracic vertebrae of Paakniwatavis are non-heterocoelous, but distorted. Then, go on to explain why in the context of others.

This has now been revised.

938: K-Pg, Tertiary is obsolete and the term Paleogene is used elsewhere in this manuscript

This has now been corrected.

953: pseudodontorns

This has now been corrected.

975-979: run-on sentence about calibration points morphs into a string of allusions to preservation, phylogeny, ecology, behavior and biogeography.

This sentence has now been broken up.

Figure 5 needs to be deleted and redone from scratch. Taxa are omitted from the phylogeny. The artist’s reconstruction of Paakniwatavis is patently misleading with regard to the skull, sternum, costae, as detailed in previous review. The ancestral state reconstruction does not discriminate between faint ridges on the palate of screamers (and other birds unmentioned) and the pronounced and highly specialized rhamphothecal lamellae of extant anatids, nor between the semipalmate digits of Anseranas and the fully palmate digits of anatids. It also invokes the evolution of said ‘webbing’ after the divergence of anhimids and Paakniwatavis, contrary to the text.

The artist's rendition has now been removed. No taxa are omitted from the phylogeny. Some taxa had to be matched by genus for this analysis (this is the combined Bayesian analysis, not the morphological analysis) as explained in the Methods section. *Wilaru* was not included in either analysis and the reasoning for this is thoroughly explained in the manuscript. The ancestral state reconstructions are a paired down version of the optimizations produced by Mesquite, and these can bee seen in the matrix folders on Morphobank. We only included the earliest and most important optimizations. Readers that require further details of the optimizations may refer to these files. There was not enough data to tell how developed ancestral rhamphothecal lamellae was or how webbed the feet were in ancestral states. We only included optimizations that were confident in whether full webbing was present due to the uncertainty of the ancestral state optimizations and discuss the nuance further in the text.

The provided dryad link returns the message "the page you are looking for does not exist." I had to request an alternate working link by an Editor of PLoS One. Therein exist raw unsegmented CT slices, and far fewer than the thousands one would expect. These files might be useful for the rare individual who might want to expend the significant time and effort to personally segment the images from scratch, and as such they appropriately need to be deposited and made available. However, they will be of no interest to nearly all readers who will want to verify character descriptions and scoring of segmented structures in the form of stl files. The stl files are not available and they must be.

These are all of the slices we have. The specimen was scanned by the University of Chicago and it is extremely difficult to scan these fossils due to the nature of the matrix, their fragility, and the small size of the scanner. This is the best scan we could acquire of the specimen as allowed by the Field Museum. The Editor has let us know that all data access issues were resolved. The slices can be worked with in imageJ, Aviso or any other CT imaging software and reveal much about the character states.

Fig S4: Anatalavis is labeled. Nettapterornis?

This has been corrected.

All one need do is scan the tracked changes in manuscript R1 to see that the only substantive changes in R2 are the addition of paragraphs about Anachronornis, Danielsavis, and Perplexicervix, as though this was the only thing that was requested by reviewers. The authors dispute nearly every comment by reviewer 2, rather than conceding that any might be well founded. Furthermore, the updated matrix including Anachronornis, Danielsavis, and Perplexicervix that is alleged (523-525) to be deposited in morphobank is not in fact present in the morphobank folder that is linked to this submission.

The matrix version that includes the new taxa has now been uploaded to Morphobank. We have also thoroughly redone the description.

I can understand the frustration expressed by the authors in the response to reviewers when they say that they believe they uploaded all the relevant supplementary materials but the reviewer says otherwise. But for whatever reason, the referenced files (i.e., updated matrix and stl files of

the specimen) do not exist in morphobank or dryad or the supplementary document. The files that do exist were certainly created by the authors, so it seems possible that if these were updated then the updates were not saved or the proper links to them were not passed along to the reviewers.

The Editor has let us know that all data access issues were resolved. We have provided links here and to the Editor and thus there is nothing else we can do to mitigate this issue.

Paakniwatavis is a very important fossil that needs to be published. "A tenet of scientific inquiry is reproducibility"(Steenwyk et al 2023 https://doi.org/10.1038/s41576-023-00620-x). In its present form, this manuscript does not permit reproducibility. I cannot independently verify the results reported in this manuscript without necessary image files and the complete data matrix.

Please see comments above.
