# Supplementary material for: A new Paleogene fossil and a new dataset for waterfowl (Aves: Anseriformes) clarify phylogeny, ecological evolution, and avian evolution at the K-Pg Boundary
Source: PLoS One. 2024 Jul 30;19(7):e0278737. doi: 10.1371/journal.pone.0278737 (PMC11288464; doi:10.1371/journal.pone.0278737)
Supplement: Attachment — Submitted filename: Response_to_Reviewers.docx [file pone.0278737.s009.docx]

**Response to the concerns of the Editor:**

- Revaluate or reinforce the systematic attribution based on reviewers’ comments.

Our response: The figures that show resulting trees clearly show that we recover *Paakniwatavis grandei* as a stem anseranatid across all analyses, and we provide character evidence and anatomical description that supports this result. We did not feel that it should be officially assigned to stem-Anseranatidae within the Systematic Paleontology section of the paper, as its position should be confirmed more robustly in time with the addition of new fossils by future workers.

- Complete and extend the comparisons, especially with the Anhimidae.

Our response: We agree that further comparison between the new specimen and Anhimidae is useful for future workers and have now included this in the description. The rest of the description is extensive in its comparison between the new species and extant and extinct anseriform taxa.

- Make the synonymy of Diatryma with Gastornis (or explain why you disagree), which are not terror birds. This term characterizes another family of giant birds, the Phorusrhacidae.

Our response: We have removed the word “terror bird” from the manuscript. The authors claim that we synonymize *Diatryma* and *Gastornis*, which is untrue. *Gastornis* is actually a junior synonym of *Diatryma*; however, Due to the extremely scarce and fragmentary nature of the fossil record of *Gastornis* versus the nearly complete skeletons of *Diatryma* that have been recovered, we believe that the relationships of these taxa must be reassessed critically if/when further specimens of *Gastornis* are recovered. Thus we chose to be cautious and carefully treat *Diatryma* and *Gastornis* as separate taxa throughout the manuscript.

- Several important papers dealing with the topic are missing. This is stated by the two reviewers. Please consider them in your revision.

Our response: Reviewer 1 calls for the addition of anhimid-like fossils based on their assertion that we find *P. grandei* to be the sister taxon of Anhimidae. This is incorrect, as we find *P. grandei* to be the sister taxon of Anseranatidae. We have already included the most complete anhimid fossil known, *Chaunoides*, in the dataset, and it would be out of the scope of this work to include further anhimid fossils, especially as they are too recent in age to provide phylogenetic significance.

Reviewer 2 claims that we need to add publications within the following sentence, where we are simply listing which taxa were found in Locality H within the Green River Formation. We only cited Feduccia and Martin (1976) as the original publication that listed these taxa since we were simply listing which taxa were found at the Green River Formation in this area. We never stated that we were providing a complete literature review of all of the publications for each taxon of the Green River Formation, and to do so would be out of the scope of this study. Our literature review for the manuscript as a whole is robust and as unbiased as possible, and it is unprofessional malignment to use this as an excuse to call our literature “selectively incomplete” and “biased” as reviewer 2 did. Here is the original sentence of the manuscript in which Feduccia and Martin (1976) is cited: “Locality H has thus far yielded the highest number of avian fossils comprising lithornithids Palaeognathae; Nesbitt and Clarke, 2016), *Gallinuloides wyomingensis* Eastman 1900 (see also Weidig, 2010; galliform), two frogmouth-like specimens (Nesbitt et al., 2011), a possible oilbird (Olson, 1987), four frigatebirds(Olson, 1977; Olson and Matsuoka, 2005; Stidham, 2015), an ibis-like taxon (Smith et al., 2013), a turaco (Field and Hsiang, 2018), two *Messelornis nearctica* specimens (Hesse, 1992; Weidig, 2010), a jacamar-like bird (Weidig, 2010), a hoopoe-like bird (Grande, 2013), stem rollers (Coraciiformes; Ksepka and Clarke, 2010), stem parrots (Ksepka and Clarke, 2011), additional taxa within Telluraves (Feduccia and Martin, 1976; Ksepka et al., 2019), and several unknown birds (Grande, 2013).”

- Please consider adding pictures of important anatomical details that are not visible in the present figures.

Our response: We will consider this; however, we included as many visualizations and as much of the CT data in the manuscript as possible and even included CT segmentations of the elements referenced on Morphobank, which is above and beyond what is often provided. In addition to this we have provided many line drawings, photographs, comparative drawings and photographs, and CT visualizations in the figures, again much more than is usually included in extinct avian publications.

**Addressing of Journal requirements:**

1. Please ensure that your manuscript meets PLOS ONE's style requirements, including those for file naming. The PLOS ONE style templates can be found at 
https://journals.plos.org/plosone/s/file?id=wjVg/PLOSOne_formatting_sample_main_body.pdf and 
https://journals.plos.org/plosone/s/file?id=ba62/PLOSOne_formatting_sample_title_authors_affiliations.pdf

The manuscript has been revised to meet PLOS ONE’s style requirements, with the exception being that I am still finishing the numbering for the References. I deeply apologize; due to my ongoing health issues I was unable to complete them in time but will be sure to complete them promptly.

2. Please take this opportunity to be sure you have met all of our guidelines for new species. For proper registration of a new zoological taxon, we require two specific statements to be included in your manuscript.

(1) In the Results section, the globally unique identifier (GUID), currently in the form of a Life Science Identifier (LSID), should be listed under the new species name, for example:
 
Anochetus boltoni Fisher sp. nov. urn:lsid:zoobank.org:act:B6C072CF-1CA6-40C7-8396-534E91EF7FBB
Another LSID for the manuscript itself should also appear within the Nomenclature statement. You will need to contact Zoobank (zoobank.org/About) to obtain a GUID (LSID). You should receive one LSID for your manuscript and a separate, unique LSID for the new species.

(2) Please also insert the following text into the Methods section, in a sub-section to be called ""Nomenclatural Acts"":
 
The electronic edition of this article conforms to the requirements of the amended International Code of Zoological Nomenclature, and hence the new names contained herein are available under that Code from the electronic edition of this article. This published work and the nomenclatural acts it contains have been registered in ZooBank, the online registration system for the ICZN. The ZooBank LSIDs (Life Science Identifiers) can be resolved and the associated information viewed through any standard web browser by appending the LSID to the prefix ""http://zoobank.org/"". The LSID for this publication is: urn:lsid:zoobank.org:pub: XXXXXXX. The electronic edition of this work was published in a journal with an ISSN, and has been archived and is available from the following digital repositories: PubMed Central, LOCKSS [author to insert any additional repositories].
 
All PLOS ONE articles are deposited in PubMed Central and LOCKSS. If your institute, or those of your co-authors, has its own repository, we recommend that you also deposit the published online article there and include the name in your article.
Following a recent ruling by the International Commission on Zoological Nomenclature, electronic journals are now a valid format for publication of new zoological taxa.

In order to ensure the valid publication of your new species, please be sure to include the updated version of Nomenclatural Acts (above). A complete explanation of our guidelines for publishing new species can be found on our website: <http://www.plosone.org/static/guidelines#zoological>.

This has now been corrected within the manuscript.

3. We note that the grant information you provided in the ‘Funding Information’ and ‘Financial Disclosure’ sections do not match.

When you resubmit, please ensure that you provide the correct grant numbers for the awards you received for your study in the ‘Funding Information’ section.

This will be corrected upon submission of the revisions into the submission system.

4. Thank you for stating the following in the Acknowledgments Section of your manuscript:

"..We thank Joel Cracraft, Paul Sweet, Mark Norell, Ruth O’Leary and Carl Mehling of AMNH for access to the Ornithology and Vertebrate Paleontology Collections and thank the Ornithology Department of AMNH for the Ornithology Collections Study Grant that aided in funding this work.."

We note that you have provided funding information that is not currently declared in your Funding Statement. However, funding information should not appear in the Acknowledgments section or other areas of your manuscript. We will only publish funding information present in the Funding Statement section of the online submission form.

Please remove any funding-related text from the manuscript and let us know how you would like to update your Funding Statement. Currently, your Funding Statement reads as follows:

"This project was supported by a National Science Foundation GRFP (https://www.nsfgrfp.org/) award (to G.M., grant number DGE-16-4486), an Ornithology Collections Study Grant from the American Museum of Natural History (https://www.amnh.org/research/vertebrate-zoology/ornithology ;to G.M., 2019) and the Jackson School of Geosciences (https://www.jsg.utexas.edu/ ;G.M. and J.A.C). The funders had no role in study design, data collection and analysis, decision to publish, or preparation of the manuscript."

Please include your amended statements within your cover letter; we will change the online submission form on your behalf.

The Funding Statement does not need to be amended, I had accidentally included one of the funding sources that I already cited in the Funding Statement in the Acknowledgments. All funding related statements from the manuscript have been removed with the exception of those included in the Funding Statement.

5. In your Data Availability statement, you have not specified where the minimal data set underlying the results described in your manuscript can be found. PLOS defines a study's minimal data set as the underlying data used to reach the conclusions drawn in the manuscript and any additional data required to replicate the reported study findings in their entirety. All PLOS journals require that the minimal data set be made fully available. For more information about our data policy, please see http://journals.plos.org/plosone/s/data-availability.

Upon re-submitting your revised manuscript, please upload your study’s minimal underlying data set as either Supporting Information files or to a stable, public repository and include the relevant URLs, DOIs, or accession numbers within your revised cover letter. For a list of acceptable repositories, please see http://journals.plos.org/plosone/s/data-availability#loc-recommended-repositories. Any potentially identifying patient information must be fully anonymized.

Important: If there are ethical or legal restrictions to sharing your data publicly, please explain these restrictions in detail. Please see our guidelines for more information on what we consider unacceptable restrictions to publicly sharing data: http://journals.plos.org/plosone/s/data-availability#loc-unacceptable-data-access-restrictions. Note that it is not acceptable for the authors to be the sole named individuals responsible for ensuring data access.

We will update your Data Availability statement to reflect the information you provide in your cover letter.

This statement is incorrect. The original Data Availability statement in the manuscript specified that the minimal dataset is available via Morphobank. All data (with the exception of CT tiffs, which are now available on DRYAD using the temporary link provided in the cover letter) was made available via Morphobank using the reviewer log in that was included both in the submission and in the cover letter.

I have pasted the Morphobank log in information below for your convenience:

Log in URL: <https://morphobank.org/index.php/LoginReg/form>

Project number (enter into email address field): 4001

Password: GreenRiver725

6. We note that you have stated that you will provide repository information for your data at acceptance. Should your manuscript be accepted for publication, we will hold it until you provide the relevant accession numbers or DOIs necessary to access your data. If you wish to make changes to your Data Availability statement, please describe these changes in your cover letter and we will update your Data Availability statement to reflect the information you provide.

These changes have now been made in both the manuscript and the new Data Availability statement has been provided in the cover letter.

7. Please include captions for your Supporting Information files at the end of your manuscript, and update any in-text citations to match accordingly. Please see our Supporting Information guidelines for more information: http://journals.plos.org/plosone/s/supporting-information.

These have now been added.

**Response to Reviewers:**

Reviewer #1: You make extensive comparisons between the new taxon and extant and fossil duck-billed anseriforms (Presbyornis, Anatalavis, Anseranatidae, and Anatidae). However, just because of the absence of a duck-like beak, it is fairly obvious that the fossil is not very closely related to any of these taxa. What would instead be needed are more detailed comparisons with the Anhimidae, which is particularly true for the differential diagnosis, where Anhimidae are only mentioned once (and not differentiated from the new taxon). The description also mainly focuses on comparisons with non-anhimid taxa, which is difficult to understand given the fact that the fossil is much more similar to the Anhimidae than to the Anseranatidae and Anatidae.
Also, at the beginning of the results part you write that "Paakniwatavis grandei is recovered across all analyses as the sister taxon of Anseranatidae". However, this is not true, as is obvious from the tree you show, where it is the sister taxon of Anseranatidae + Anhimidae.

This reviewer has misread both the figures and results, which have been thoroughly checked and rechecked many times by both Dr. Musser and Dr. Clarke, as this manuscript comprised the final chapter of Dr. Musser’s dissertation. The combined data tree (Figure 5) and both morphological trees clearly show that we recover *P. grandei* (FMNH PA725) as the sister taxon to *Anseranas semipalmata*+Anatidae. If anything, it is likely that the reviewer is confusing the new species (*P. grandei*) with a previously published fossil, *Chaunoides*, which we included in the dataset and recover as the sister taxon to Anhimidae across all three trees. It is unfortunate that this reviewer did not read carefully enough to give an insightful review and/or is allowing bias about the affinities of the fossil specimen to cloud their judgement without discussing the results garnered from analysis of our data, and that this has caused our manuscript poor reception during the submission process.

Furthermore, this reviewer’s statement makes it clear that they did not read our careful anatomical description and characterization of anseriform beak type that is emphasized throughout the manuscript. This detailed examination of anseriform beak type is also reflected in the character scoring of the matrix and has important implications for anseriform morphology, ecology, behavior and evolution as detailed in the discussion. We created these descriptions and definitions based on both our own careful anatomical observation as well as the observations of other workers (most notably Olsen). Unlike the statement provided by the reviewer, we do not define *Anseranas semipalmata* as having a wide “duck-like” beak; in fact, we consistently state that *Anseranas* has a more narrow “goose-like” beak like that of the new species. We consistently state throughout the manuscript that although many Anseriformes possessed wide, “duck-like” beaks (eg. Those of *Presbyornis* or extant ducks such as *Anas*), many Anseriformes have a more narrow “goose-like” beak (eg. Those of *Anseranas* and geese such as *Branta*).

Due to *P. grandei* being recovered as the sister taxon of *Anseranas semipalmata*+Anatidae across all trees (and across both combined Bayesian and morphology-only parsimony analyses), we did not feel it necessary to compare the new species in further detail to Anhimidae; however, we agree that it may be important for comparison of future specimens, and thus have added further comparison of Anhimidae and the new species throughout the Description in the manuscript.

The omission, of the Anhimidae from most of your comparisons and discussion is difficult to understand and results in awkward statements like ” We recover Paakniwatavis grandei as the sister taxon to an Anseranas+Anatidae” (beginning of discussion). Actually, you recover the fossil as the sister taxon of an “Anhimidae + (Anseranatidae + Anatidae)) clade, and given the great similarity to screamers in some features (e.g., the massive furcula and the shape of the coracoid), I consider it highly likely that your fossil is a stem group anhimid.

I also do not quite understand, why any comparison with the Anhimidae are omitted from the discussion, as these certainly are the extant anseriform taxon that is most similar to the fossil with regard to skull morphology. You emphasize a potential significance of the new fossil for an understanding of anseriform evolution, but without any reference to the Anhimidae, this significance remains elusive. If this fossil is indeed a stem group anseriform, it would show that many features of the Anhimidae are plesiomorphic for anseriforms, as has been assumed by earlier authors. If it is a stem group anhimid, than the leg morphology may suggest that screamers were more aquatic in the past. In any case, a more balanced discussion is needed that includes more comparisons with the Anhimidae.

Again, the reviewer has incorrectly reported the results here. Please see above comments and revisions that already address these issues.

What is needed is a more thorough discussion of the affinities pf the fossil. You note that “Seven unambiguous and three ambiguous optimized synapomorphies of the quadrate, mandible, furcula, scapula, carpometacarpus, tarsometatarsus and pedal phalanges support placement of P. grandei as a stem anseranatid.” However, what exactly does this mean? Are these characters present in crown group anseriforms but absent in the fossil? In the following text it rather reads as if these features were also present in the fossil, in which case they would not support a stem group position.

This sentence is a standard one used in phylogenetic manuscripts that provides the resulting synapomorphies that unite *P. grandei* with Anseranatidae (*Anseranas*+Anatidae). This means that these character states are present in both *P. grandei*, *Anseranas* and many extinct and extant included Anatidae. These character states additionally are not found in Anhimidae or *Chaunoides*; thus, they comprise the strongest evidence provided by the PAUP* and/or Bayesian algorithms that *P. grandei* is the sister taxon of *Anseranas*+Anatidae.

In my opinion, the comments on the evolution of syrinx morphology are out of place in the discussion, because the new taxon does not contribute to these issues. Given the unstable position of the fossil (and other anseriforms) in your analyses, ancestral state reconstructions are of rather limited value.

As clearly stated throughout the manuscript, the new species is not the only significant contribution of this work. The second (and perhaps larger and more important contribution) is a new morphological dataset for Anseriformes that includes more extant and extinct taxa than any previous anseriform-focused dataset. This dataset is especially critical for the study of future workers as it includes reassessment of and scorings from many specimens of *Presbyornis* and *Telmabates antiquus* that have not been included in previous studies. This included re-evaluation of all *Presbyornis* material housed in the Vertebrate Paleontology Collection of the Smithsonian National Museum of Natural History and all *Telmabates* material housed in the Vertebrate Paleontology Collection of the American Museum of Natural History. This dataset comprised years of careful examination of specimens and several trips to examine specimens housed in both national and international natural history museums. Additionally, this new dataset is cutting-edge as it is one of the first to include a wide range of total evidence characters comprising soft tissue characters, behavioral and ecological characters, and characters related to the syrinx. These characters and their ancestral state reconstructions have never been examined in this comprehensive a manner within Anseriformes, and provide important insight into the evolution of this group. It will be important for future workers to be able to compare how both the relationships and ancestral state reconstructions within this dataset change based on the addition of new extinct and extant taxa.

The fact that you recover a “Diatryma =Gastornis]+Pelagornithidae group” challenges the appropriateness your data set and it would be interesting to know what the character evidence for this grouping might be. Given the highly uncertain affinities of these fossils, I would also refrain from calling them “stem anseriform”, which is at least unlikely for pelagornithids.

We never stated that *Diatryma* = *Gastornis*, and in fact carefully pointed out how our results differ than those presented in previous publications that do include *Gastornis* (eg. Worthy et al. 2017). We did not include *Gastornis* in our dataset. The character evidence for the *Diatryma*+Pelagornithidae group placement is freely available through analysis of the nexus file, which WAS MADE AVAILBLE TO REVIEWERS VIA MORPHOBANK USING A REVIEWER ONLY LOG-IN. The data will also be made publicly available upon acceptance for publication. Both authors have carefully reviewed the synapomorphies supporting each group across all analyses. The phylogenetic positions of both *Diatryma* and *Gastornis* are largely unknown due to a lack of robust evidence, although our placement of *Diatryma* as the sister taxon of Pelagornithidae is consistent with the results of Bourdon (2005). Similarly, our placement of Pelagornithidae within a group that is sister to Anseriformes is similar to the placements of other workers. Field et al. (2020) recovered the Pelagornithidae either as the sister taxon to an Anseriformes(Conflicto+Anatalavis) group or as the sister taxon of Charadriiformes, and Mayr et al. (2021) recovered a polytomy comprising Pelagornithidae, Galloanseres and Neoaves. Thus our phylogenetic placements of both *Diatryma* and Pelagornithidae are reasonable ones and not without precedent.

I also believe that the title of the manuscript is not quite appropriate for this paper ("A new Paleogene fossil and a new dataset for waterfowl (Aves: Anseriformes) clarify phylogeny, ecological evolution, and avian evolution at the K-Pg Boundary"). Not only does your phylogenetic data set yield conflicting results, but I also do not see why this early Eocene fossil elucidates avian evolution at the K/Pg boundary. Actually, what you describe is a "screamer-like anseriform from the Green River Formation", in line with previous proposals that such birds were present in the early Eocene of North America (see comments above).

Contrary to the reviewer’s statement, the phylogenetic dataset yields no conflicting results for the new species and minimally differing results for some taxa across analysis type (Bayesian analysis of the combined dataset versus parsimony analysis of the morphological dataset). The discussion lays out a detailed argument as to how this dataset and the placement of this new species is relevant to better understanding the evolution of Anseriformes since the K/Pg boundary, especially since many of the included anseriform fossils are of Cretaceous age. The reviewer’s fixation on reducing this manuscript to the description of an anhimid-like anseriform (which, as noted above, is not the correct phylogenetic placement of this fossil) reveals their lack of understanding of the manuscript and their bias as to the phylogenetic relationships of this fossil.

- throughout the manuscript: Why has the North American gastornithiform been assigned to the taxon Diatryma rather than to Gastornis? Most current authors synonymize both taxa.

As noted above, we have not claimed that Diatryma is a gastornithiform. We removed the word “terror bird,” as this may have been what was confusing the reviewer, but other than this incidence we carefully treat *Gastornis* and *Diatryma* as separate taxa.

p. 4, line 95: why do you consider the beak shape of the Paleocene Conflicto to be “largely unknown”? The holotype includes a nearly complete skull with nicely preserved beak.

We state that the beak shape is unknown because although the center of the beak is preserved on the left side, the right side of the beak and the left edge and premaxilla of the beak are not preserved. In addition, the nasofrontal hinge is broken (this is even noted by the authors of the paper describing *Conflicto*, Tambussi et al. 2019). Due to this breakage, it is not possible to tell whether *Conflicto* possessed a more narrow “goose-like” beak or a wider “duck-like” beak as defined throughout our manuscript. Please see Figures 4 and 3 below from Tambussi et al. 2019.

- you should perhaps indicate that screamer-like Eocene anseriforms have already be identified by Peter Houde. These were mentioned by, e.g., Feduccia 1999 and Mayr 2009, 2017, 2022. It is likely that these fossils are closely related to your specimen. It would also be good to cite at least some of the aforementioned studies that indicate the presence of anhimid-like anseriforms in the early Eocene of North America.

We have already included Chaunoides, which is one of the most complete screamer-like fossils, and have provided thorough comparisons between this taxon and the new species. The reviewers recommendations are inappropriate and based on bias as to the phylogenetic position of this fossil (see above comments).

- line 128: “partial skeleton and tracheal rings” – I would consider the tracheal rings to be part of the skeleton (usually one also do not separately mention, for example, scleral rings).

Tracheal rings are not considered part of the skeleton as they are made up of cartilage, and not bone. Therefore it is important to distinguish them as a different structure. Scleral rings, in contrast, are made up of bone.

- line 130: you hypothesize that part of the skeleton has been” eroded”, by I think that this is an unfortunate wording. To me it rather appears as if the central part of the skeleton was dissolved by an acidic environment (possibly owing to the putrefaction of the trunk). Similar taphonomies are known from bog bodies and some birds from Messel.

We have thoroughly cited and describe other similar instances of this phenomenon in the manuscript. We think that “eroded” versus “dissolved” does not significantly change the meaning of the taphonomy described within the manuscript.

- line 178: “dorsoventrally thick retroarticular process” – wouldn’t “dorsoventrally deep” be better (also in other places of the manuscript)?

We thank the Reviewer for this suggestion; however, we chose to use the word “thick” as we are describing the breadth of a structure and did not want the wording to become confusing. “Deep” brings to mind the depth of a sulcus, for example. Due to this, we did not make the suggested changes in the manuscript.

- line 318: “cranio-medial aspect ” – this should be “cranio-ventral”

This has now been changed.

- line 320: The humeral head – what is meant here – the proximal end of the bone or the caput humeri? To avoid ambiguity, I suggest to use the proper anatomical terms throughout the manuscript.

"Humeral head” has now been changed to “caput humeri” throughout the manuscript.

- line 322: “tuberculum supracondylare ventrale” (add “e”)

This has now been added.

- line 334: ” and has broken onto the skull.” – is this correct English? Sounds awkward to me.

This has now been changed to “has broken on top of the skull.”

- line 368: “supratendineus” (add “e”)

This has now been changed throughout the manuscript.

- line 371 “two sulci are present” – because you identify these sulci in the figure, some comments in the description would be appropriate. Also you may cite Mayr (201t5) for the identification of the hypotarsal sulci.

We have added a more robust description of the sulci of *P. grandei* and compare it to relevant Anseriformes. We have also added the citations for Mayr (2016) as suggested.

- line 373: “metatarsal trochlea III” – why not use the correct anatomical term (trochlea metatarsi III)?

This has now been corrected.

- line 526: Presbyornis” (add “s”)

This has now been corrected.

- line 566: “terror birds such as Gastornis” – gastornithiforms are no “terror birds”. This name is used for the Phorusrhacidae. It is also confusing that you use Gastornis here, whereas you classify the North American species as “Diatryma” (see above).

This has now been corrected.

- line 586: “supratendineus” (substitute “o” with “e”)

This has now been corrected.

- line 768 ff.: “all analyses recover the Cretaceous-Paleogene taxa Vegavis, Anatalavis, Conflicto, 769 Presbyornis and Telmabates within a clade that is the sister taxon to Anatidae”. Here, it would be good to note that some evidence against this classification exists, such as the absence of long retroarticular processes in Vegavis (see Mayr et al. 2018: On the taxonomic composition and phylogenetic affinities of the recently proposed clade Vegaviidae Agnolín et al., 2017 ‒ neornithine birds from the Upper Cretaceous of the Southern Hemisphere).

The retroarticular process within Vegavis is not absent; it is present but broken, so we do not know how long the retroarticular process would have been (see a portion of Extended Data Figure 2 below from Clarke et al. 2005). We have cited all instances of conflicting phylogenetic studies throughout the manuscript. We do not feel that the presence of a character in itself is evidence “against” a relationship, as these phylogenetic relationships are ultimately determined by the phylogenetic algorithms that optimize synapomorphies and not by our opinion of single character states. Therefore we have decided to not include the Reviewer’s suggestion.

- line 784: “amphiplatyan“ – is this a correct word?

Yes, it is “flat at both ends. used of vertebrae having both anterior and posterior surfaces of the centrum flat” according to the Mirriam-Webster Dictionary (<https://www.merriam-webster.com/dictionary/amphiplatyan#:~:text=%3A%20flat%20at%20both%20ends,surfaces%20of%20the%20centrum%20flat>).

- line 1984: “Ericson, Per G. P.” – abbreviate the surname Per.

This has now been corrected.

**Reviewer #2: This paper represents an impressive study of a new and important fossil that bears on the early evolution of waterfowl. Overall, I have the impression that the authors have over-interpreted what is a preservationally compromised specimen. I am unable to fully assess whether the analyses have been undertaken rigorously as I do not have access to the author's data to independently verify it.**

First, the reviewer did have access to all of the data with the exception of the CT scan data via Morphobank (see log in information below). A log in was provided to reviewers within the submission and was supplied via the cover letter. I also have now uploaded the CT tiffs to DRYAD, which can be viewed prior to publication using this temporary link: https://datadryad.org/stash/share/z_tvwBWTt5tXk9pQmCWclxGWV-7bKlR0SuTpztro_tc . The dataset has been assigned the following unique DOI, which will become live once the paper and data are published: 10.5061/dryad.v15dv4208.

Second, this specimen is one of the best preserved Paleogene avian specimens in the world. Avian Paleogene specimens often comprise crushed, fragmentary skeletons or feathers, whereas this is a nearly complete specimen preserved largely in three dimensions, and we were even able to acquire much additional information about the morphology of the specimen via CT scanning. The CT scan of the specimen was a two part multiscan conducted by the University of Chicago via the Field Museum of Natural History Vertebrate Paleontology Division.

I have pasted the Morphobank log in information below for your convenience:

Log in URL: <https://morphobank.org/index.php/LoginReg/form>

Project number (enter into email address field): 4001

Password: GreenRiver725

I hope my detailed comments below will prove helpful in necessary moderate revision.

23 - It is incorrect to say that Anseriformes have a comparatively rich, Cretaceous fossil
record

We provide evidence in the manuscript as to how Anseriformes have a comparatively rich Cretaceous-Paleogene fossil record, and state that this is RELATIVE to other avian taxa. Most avian taxa currently have no Cretaceous-Paleogene fossil record.

36-37 - Abstract – it is stated that the new Eocene taxon as a stem anseranatid across all analyses, yet it is not assigned or diagnosed as such. Instead it is simply Anseriformes

The figures that show resulting trees clearly show that we recover *Paakniwatavis grandei* as a stem anseranatid across all analyses, and we provide character evidence and anatomical description that supports this result. We did not feel that it should be officially assigned to stem-Anseranatidae within the Systematic Paleontology section of the paper, as its position should be confirmed more robustly in time with the addition of new fossils by future workers.

45 - Diatryma is a junior synonym of Gastornis

This statement is incorrect—*Gastornis* is actually a junior synonym of *Diatryma*. Due to the extremely scarce and fragmentary nature of the fossil record of *Gastornis* versus the nearly complete skeletons of *Diatryma* that have been recovered, we believe that the relationships of these taxa must be reassessed critically if/when further specimens of *Gastornis* are recovered, and thus chose to be cautious and treat *Diatryma* and *Gastornis* as separate taxa.

65 - I don’t recall that Olson and Feduccia 1980 claimed that Anhimidae possessed pedal webbing

From Olson and Feduccia 1980: “Although differing from the Anhimidae in many respects, Anseranas certainly represents a morphological stage similar to that through which the Anhimidae must have passed in becoming increasingly terrestrial (as shown by the reduction of webbing in the feet) and in relinquishing filtering as a means of feeding (as shown by the strong nail and reduced lamellae of the bill). We have discovered in the Anhimidae an overlooked character that strongly supports their anseriform derivation, namely the presence of vestigial lamellae. Although several authors (Parker, 1863; Garrod, 1876; Mitchell, 1895) have briefly noted the large fleshy tongue in Chauna, none seems to have detected these lamellae. Indeed, Pycraft (1910:49) specifically states that the Anhimidae lack ‘the lamellae along the edges of the beak, so characteristic of the Anseres.’ This is not the case, and in reality the screamers do retain, in a highly modified form, these most diagnostic of anseriform structures.”

89-90 - early Eocene Fossil Butte Member (FBM; 51.97 ± 0.16 Ma; Smith et al., 2010) of the Green River Formation

It is unclear what the reviewer is requesting be changed here as this line matches that within the manuscript.

90-91 - The taxon was originally illustrated with a prospective referral by Storrs Olson to Heliornithidae (finfoots; Grande 2013); On the contrary, Grande included this fossil in his section “Waterfowl (Anseriformes…” He referred to this as an “undescribed possible waterfowl” and “an anseriform-like bird” (p225). There is no mention of Storrs Olson or heliornithids.

-“?Heliornithidae” is written on the specimen tag and on the specimen scan sheet (which was created by April Neander) under “Specimen Number.” Lance Grande personally communicated to us that Storrs Olson had tentatively referred this specimen to Heliornithidae. We have now rewritten this sentence in the manuscript to clarify this and have cited the personal communication.

165 – “palaeognathid” is neither a family nor a word

“Palaeognathid” has now been changed to “Palaeognathae.”

167 – frigatebird is one word

All instances of “frigate bird(s)” have been changed to “frigatebird(s).”

168 – previously this is described as an unidentified ibis-like taxon

We have now changed this “ibis” to be referred to as “an ibis-like taxon.”

171 – the citation of Feduccia and Martin 1976 is odd since the birds they originally described have all been rediagnosed, without exception, yet the authors of this manuscript have avoided citing relevant original literature of other taxa. The literature review is selectively incomplete, and perhaps biased.

We cite this publication within the following sentence, where we are simply listing which taxa were found in Locality H within the Green River Formation. We only cited Feduccia and Martin (1976) as the original publication that listed these taxa since we were simply listing which taxa were found at the Green River Formation in this area. We never stated that we were providing a complete literature review of all of the publications for each taxon of the Green River Formation, and to do so would be out of the scope of this study. Our literature review for the manuscript as a whole is robust and as unbiased as possible. Here is the original sentence of the manuscript in which Feduccia and Martin (1976) is cited: “Locality H has thus far yielded the highest number of avian fossils comprising lithornithids Palaeognathae; Nesbitt and Clarke, 2016), *Gallinuloides wyomingensis* Eastman 1900 (see also Weidig, 2010; galliform), two frogmouth-like specimens (Nesbitt et al., 2011), a possible oilbird (Olson, 1987), four frigatebirds(Olson, 1977; Olson and Matsuoka, 2005; Stidham, 2015), an ibis-like taxon (Smith et al., 2013), a turaco (Field and Hsiang, 2018), two *Messelornis nearctica* specimens (Hesse, 1992; Weidig, 2010), a jacamar-like bird (Weidig, 2010), a hoopoe-like bird (Grande, 2013), stem rollers (Coraciiformes; Ksepka and Clarke, 2010), stem parrots (Ksepka and Clarke, 2011), additional taxa within Telluraves (Feduccia and Martin, 1976; Ksepka et al., 2019), and several unknown birds (Grande, 2013).”

187-244 – numerous characters described in the Differential Diagnosis and Description are insufficiently illustrated for the reader to independently verify or even understand the features described. I cannot understand how details of the scapula or sternum can be inferred from Figure 1. I have not been given access to supplemental CT images. Without better documentation, I am left with the impression that the interpretations far outstrip the evidence.

We included as much of the CT data in the manuscript as possible and even included CT segmentations of the elements referenced on Morphobank, which is above and beyond what is often provided. In addition to this we have provided many CT visualizations in the figures, again many more than is usually included in extinct avian publications, along with countless photos and line drawings.

Again, contrary to the statements of this reviewer, all matrices and supplemental data (including CT segmentation files) except for the CT tiffs were made available to the reviewers through the appendix and via Morphobank using a reviewer-only log in. We would have made CT data available as well and are happy to; however, the issue is that Morphobank is having issues with being able to host that high an amount of CT data. We have now uploaded the CT tiffs to Dryad to mitigate this issue. They are now available under the following temporary link: <https://datadryad.org/stash/share/z_tvwBWTt5tXk9pQmCWclxGWV-7bKlR0SuTpztro_tc> (DOI 10.5061/dryad.v15dv4208, which will become active upon publication).

191-192 - I do not believe that it can be shown that the bill was narrower than the bilateral width of the paroccipital processes on the basis of the crushed specimen.

CT tiff data illustrates that even when taphonomy and slight crushing is accounted for, the bill would have been narrower than the bilateral width of the paroccipital processes (see DRYAD). This can be measured digitally. There are additionally no extant or extinct examples of a bird that has a narrow premaxilla and maxilla with a bill that was wider than the bilateral width of the paroccipital processes.

198 – “blind pneumatic foramen” is an oxymoron

The word “blind” has been removed.

207 – Is there a pneumatic foramen to show that the fossa pneumotricipitalis of the humerus is pneumatic in Paakniwatavis?

Yes, a large pneumatic foramen is present as scored in the matrix (459:1), which again was made available to reviewers via Morphobank. This is also visible on the specimen and in the CT data.

213-214 – it is unclear what is meant by “The craniocaudal lengths of manual digits II and III at the synostosis are subequal in Paakniwatavis”

This sentence has been revised as follows: The craniocaudal lengths of manual digits II and III at the synostosis are subequal in *Paakniwatavis*, whereas II is extends further distally greater than III in *Anatalavis*.

229 – medial mandibular process? retroarticular process?

The ventral angle is neither the medial mandibular process nor the retroarticular process and is clearly illustrated in Livezey and Zusi (2006) and referenced in many extinct avian and avian anatomical publications. It is the ventral protrusion of the mandibular rami between the caudal articulation for the quadrate and the angulus mandibulae (eg. Baumel and Witmer 1993).

233 - “is of”

This has now been corrected.

233-234 - distal aperture of the sulcus extensorius, not the pons

This has now been corrected.

240-241, 325, 548 – what is meant by “domed” crista

A domed crista of the fossa pneumotricipitalis in Anseriformes has been noted by other workers and is further detailed in the Appendix and character matrix. We have revised the first occurrence of the word “domed” to convey further clarity: “*Paakniwatavis* can be differentiated further from *Telmabates* based on features of the humerus and carpometacarpus*. Paakniwatavis* has a dome-liked crista that overhangs along the proximal margin of the fossa pneumotricipitalis of the humerus, whereas this crista is typical in *Telmabates*.

245 – Chaunoides is not italicized

This has now been corrected.

248 – it is unclear what is meant by “the sternal facet curves cranially”

The following sentences have been revised to say: “Within the coracoid, the primary axis of the scapular cotyla is skewed laterally in Paakniwatavis, Presbyornis, Telmabates and Anatalavis but is centralized in Chaunoides (see Figure 4). The sternal facet curves is skewed cranially in Chaunoides but is flat in Paakniwatavis.”

249 – the major metatarsal ridge does not appear to be hooked distally in Figure 4F1, F2

The metatarsal ridges do appear to be hooked distally in the Figures, the segmentation files included in Morphobank that were made available to all reviewers, and in the CT data which is now available to the reviewers as well.

264 – In what way(s) are the crania of anhimids and Anseranas similar? This surprising statement needs elaboration.

We do not claim that the crania of anhimids and Anseranas similar. We simply state that the general shape and size of the crania of the fossil is overall similar to the crania of both taxa before proceeding to compare the taxa in detail.

265 – how can either jugal be evident if they are covered by the carpometacarpus and how could the left be evident if it is the right side of the skull that is exposed?

CT data allows us to view all of the bones under the surface of the slab, which is why we opted to CT scan and carefully describe the specimen. CT data not visible on the surface of the slab was made available in the figures and in the CT segmentation files available on Morphobank. All CT tiffs are now available on DRYAD as well.

265-267 – it is not clear to me from the illustrations that the “lateroventrally projecting supraorbital crest is present”. Instead, it appears that the left prefrontal is broader than the supraorbital.

It is not possible to visually document all anatomical features in a published manuscript. All scored and described features were done so based on careful observation of the specimen, the raw CT data, and segmentation of the CT data (which has been made available to reviewers).

267 – that the postorbital process is (revealed by CT scans to be) elongate like that of most Anseriformes is particularly surprising since it is not in screamers, to which the bill of the new fossils resembles. The postorbital serves as an origin of jaw musculature that, among other characters, is associated with advanced filter feeding behavior of Anseriformes.

We cannot and do not pretend to be able to control what the morphologies of the fossil specimen are, no matter how surprising they may be.

306-307 – stout relative to what directions?

This has been changed to “A craniocaudally stout pygostyle…”

309 – are there such things as ventral and dorsal clavicles?

This sentence has been changed to: “The symphysis and ventral portion of the clavicles of the furcula are preserved in caudal aspect.”

313 – sternal (=proximal) or omal (=distal) extremities, not distal bases

This has now been changed.

314-315 - if CT images show the procoracoid to be present, then it should be included in Figure 4F

We clearly show the presence of a procoracoid process in Figure 4F but show that it is broken. We do not know what the shape of the procoracoid process was due to preservation and did not want to interpret its shape incorrectly in a figure. We simply state that a procoracoid process was present and do not describe its shape.

319 vs 230 – the descriptions of the deltopectoral crest seem a little contradictory

There is only one sentence describing the deltopectoral crest and so it is unclear how its description is contradictory: “The crista deltopectoralis is prominent, rounded and flares cranio-laterally like those of Anseriformes.”

327 – ulnar body; than that of the humerus

This has now been corrected.

329 – impressio brachialis

This has now been corrected.

344 – check grammar

This has now been corrected.

365 – is appears

This grammatical error is not present in the manuscript.

377-383 – it may be true that femur length is the best (or only) correlate of body mass among those that that could be measured, but the scaling functions are sensitive to taxonomic group. The proportions of this fossil (e.g., pre vs postacetabular pelvis) are more like that of screamers than typical anatoids, on which the slope for Anseriformes is mostly if not entirely based. In my experience, the femora of even fossils preserved in the round produce mass estimates with error ranges as high as 100%. Thus, to report this estimate to the first decimal with no error range is unacceptable.

This has now been corrected throughout the manuscript.

394 comprised of or include

“Comprise” is grammatically correct here: Extinct taxa scored from photographs when necessary comprise Ichthyornis dispar Marsh 1872 (Clarke 2004), Diatryma gigantea and Diatryma steini (Matthew and Granger 1917; Andors 1992) Pelagornis chilensis (Mayr and Rubilar-Rogers 2010), Protodontopteryx ruthae (Mayr et al. 2021), Vegavis iaai (Clarke et al. 2005, Clarke et al. 2016), Conflicto antarcticus (Tambussi et al. 2019), Anatalavis oxfordi (Olson 1999), Wilaru tedfordi Boles 2013 and Wilaru prideauxi (De Pietri 2016), Presbyornis (Olson and Feduccia 1980, Elzanowski and Stidham 2010), Chaunoides antiquus (Alvarenga 1999), Lithornis promiscuus (Houde 1988), Calciavis grandei (Nesbitt and Clarke 2016), Asteriornis maastrichtensis (Field et al. 2020), and Gallinuloides wyomingensis (Mayr and Weidig 2004).

467-504 – It is very interesting that the inclusion of Wilaru resulted in wide ranging discombobulation of the phylogeny, especially since it is so strongly supported as a member of Presbyornithidae! This, and the different mitochondrial results among anatoids that are a tad tangential to the subject here, might well be worthy of elaboration in a separate paper. What would be relevant to the subject of this manuscript, but is not reported, is whether similar eyebrow-raising results of the phylogenies reported here would accrue if Paakniwatavis was excluded. To what extent are these the effect of the fossil or of the character set? At any rate, these results cannot be dismissed as the result of Wilaru being know from only “highly fragmentary” remains, as its two species are in fact known from an abundance (embarrassingly large compared with, say, Vegavis, about which so much is written) of well preserved specimens.

First, *Wilaru* is not well supported as a member of Presbyornithidae, and as we discuss in detail in the manuscript, there are many conflicting results for its placement across previous publications. In addition, it is inappropriate to compare the preservation of fossils that are vastly different in age such as *Wilaru* and *Vegavis*. *Wilaru* is Oligocene-Miocene in age, whereas *Vegavis* is much older and is a Cretaceous fossil. Despite its age, *Vegavis* has a preserved trunk, limb bones and syrinx; whereas only limb bones and the pectoral girdle are preserved for *Wilaru*. Limb bones and many parts of the pectoral girdle (eg. The furcula and scapulae) are known to have less phylogenetic signal and thus cannot usually be robustly placed in a phylogeny. Despite our best efforts, this is what occurred with *Wilaru* in our results, and thus we present results both including and excluding this taxon. Exclusion of *P. grandei* does not change the phylogenetic relationships, regardless of whether *Wilaru* is included.

530 congruent, not confluent

This has now been corrected.

559-578 – in light of the different results reviewed in this paragraph, it would be a good idea to define what is meant by Anseriformes as it is used on line 578.

The definition of Anseriformes does not change throughout the manuscript and so it would cause further confusion to redefine the term in the results section.

576, 592 – ambiguously optimized

“Ambiguous” and “unambiguous” are adjectives here, not adverbs. This is standard terminology used in phylogenetic publications to describe the type of synapomorphy optimized by the PAUP* algorithm.

594-595 - the crista

This has now been corrected.

595 awkward sentence

The following sentence has now been revised as follows: Within the quadrate, the crista tympanica is a present and exhibits an extremely prominent crista (171:3, unambiguous), and the caudal face of the otic process is deeply concave (176:2, unambiguous).

613-614 – I am confused whether the length of the hallux was measured or inferred by ancestral state reconstruction. Regardless, if it was unusually long, then this would be similar to the condition in screamers and jacanas, which are noted for foraging from on top of floating vegetation.

Hallux length was scored into the matrix (character 705, copied below) but is not a trait that was inferred by ancestral state reconstruction. This character could not be scored for most taxa due to specimen collection limitations (eg. loss of toes in many extant specimens). The new species shares state 1 with *Presbyornis* and Anhimidae out of the small number of Anseriformes that were able to be scored. The foraging idea put forth by the reviewer is an interesting one that we will keep in mind for future study.

From the appendix: “705. Pedal I:1, length as compared to III:1: not as follows (1); I:1 about half the length of III:1 (2). Musser and Clarke (2020), character 691; Mayr and Clarke (2003), character 110; Mayr (2002), character 32; Livezey (1997), character 96.”

625 – identical to what?

This has now been corrected.

459, 643 – the authors used only 7 of the ~33 used by Hinić Frlog [misspelled throughout] and Montani in their study of skeletal correlates of swimming modes in their fossil. That Chauna was ambiguously recovered as a wing-propelled diver and that Degrange found similar morphospace among such ecologically dissimilar birds as cursorial/terrestrial paleognaths and diving loons and grebes using similar analysis make the authors appropriately circumspect of their results.

Only 7 of the 33 skeletal measurements of Hinić Frlog and Motani (2010) could be used as these are the only measurements that could be located within the fossils, and it is not possible to include additional measurements with no data for the fossils when conducting the PCA and LDA analyses. The untenable results are due to this limitation of current PCA and LDA analysis programs and have nothing to do with our phylogenetic analyses of the new species or with its assessment and description. We simply did not include the accent within Hinić’s name originally but have now added this throughout the manuscript. It is uncertain to what else the reviewer is referring here.

699 – Presbyornis, Anatalavis and to some extent Conflicto can all be characterized as having a spatulate beak, but they are each very dissimilar from one another and each more alike Stictonetta, Spatula, and Mergus, resp.

*Presbyornis* and *Anatalavis* have similarly spatulate beaks, whereas the beak shape of *Conflicto* is unknown (see above comments). We do not attempt to lump together *Stictonetta*, *Spatula*, and *Mergus* in this paragraph; we are simply stating how often a wider “duck-like beak” evolved based on our results. We are not attempting to describe beak differences between Anseriformes in greater detail.

768, 811 – Conflicto is reported by Tambussi et al to be Paleocene. No more recent publication is cited to justify citing it as Cretaceous. It is unjustifiable to assume that “Cretaceous” (also Paleocene) Anatalavis had a spatulate bill. Olson, himself, said it would have been impossible to have known that Presbyornis did until its skull was discovered.

Figure 1 below from Olson (1999) shows that *Anatalavis* had a spatulate, wide “duck-like” bill. Again, see comments above-we simply attempt to define beaks within Anseriformes as being more wide and “duck-like” or more narrow and “goose-like,” and point out the width of the bill compared to the width of the paroccipital processes. Dr. Clarke additionally has taken several photographs of the *Anatalavis* skull which we were able to reference during scoring.

814 – gastornithids is an adjective and not capitalized

This has now been corrected.

Figure 2 – The figure would benefit from additional labeling. Given the detailed description of cranial characters that cannot be discerned from the figure, there is an argument to be made to have an artist’s three-dimensional reconstruction of the skull. For a long time, I puzzled over two parallel bars of bone oriented rostrocaudally in the crushed upper bill. One of these must be the right tomium, I concluded that the other must be the left side of the mandible. But if so, then why is there apparently no elevation of the left coronoid as it so clearly is on the right side? There are various seemingly extraneous lines in panel A that appear to be artifacts. If they aren’t, then they should be labeled.

Due to the flattened nature of the specimen and the inability of a micro-CT scan (performed by the Field Museum via the University of Chicago) to provide additional detail beyond what it already provided regarding subsurface structures, it is impossible to reconstruct the skull in three dimensions. Regardless of this, the CT scan is of high quality (especially as it was a two part multiscan, which was done to provide the highest resolution possible) and allows us to identify much more additional morphology under the surface of the slab than would be possible with the naked eye or even an x-ray. The skull morphology is visible in detail both in the photos and in the CT data, and was thoroughly described in the description of the new fossil. There are no extraneous lines within the figure; however, we have added additional labels as requested by the reviewer and have added these in the figure caption as well.

Figure 3 – the furcula of Paakniwatavis (fig. 3 N, O) appears no thicker than that of Presbyornis (fig. 3 M) as claimed

The furcula of *P. grandei* is thicker than that of *Presbyornis* as both authors have examined both specimens in person; it appears more similar here due to our increasing the size of the furcula of *Presbyornis* (these images are not to relative scale). Even so, it is clear that the symphysis and ventral portion of the furcula in *P. grandei* is thicker than that of *Presbyornis*.

Figure 4 - the labeling F1-F4 is strangely inconsistent with the rest of the figure. More importantly, the caption does not identify whether the images of F1,F2 are of one tarsometatarsus from opposite sides or both right and left. F3,4 are said to be of “the same tarsometatarsus”

F1-F4 were used as we are showing the CT segmentations and CT scan slices of the tarsometatarsi of *P. grandei* within this figure so that the anatomy of the hypotarsal crests may be more easily visible to readers. The caption has now been changed according to the suggestion of the reviewer.

Figure 5 – the reconstruction is aesthetic, but somewhat misrepresentative, i.e., unfused lacrimals (not described, if so), sternum tiny, furcula narrow, the angle of dorsal and sternal costae oriented as in flightless ‘ratites’, intramembral proportions of hindlimb elements not quite as reported on lines 235, 362. Missing cervical vertebrae are indicated by dark gray. Other missing parts of the skeleton (e.g., of the sternum, proximal left tibiotarsus, ) should be, too, for consistency although much of that can be gleaned from the line drawing background of the figure.

Unfortunately, the reconstruction cannot be completely accurate due to the taphonomy of this specimen discussed in the manuscript. The reconstruction was created to represent the fossil as closely as possible to give readers an idea of what this specimen would have looked like in life. The sternum is not missing, it is present and visible in the CT data as indicated in the manuscript. The same is true of the other “missing” portions of the skeleton that the Reviewer is referring to.

There are five blank pages following character description #719 in the Appendix. Were there supposed to be illustrations or citations here?

No, this is an error caused by Microsoft Word that will be corrected once the file can be converted to a PDF.

The number of characters scored for this study are impressive, but relatively few can be scored for the new fossil. The resulting phylogeny might therefore be robust for neotaxa (however, the much larger dataset of Livezey and Zusi failed to reproduce what are known relationships even of living birds), but have little to say about the position of this fossil within it. Despite the scoring of ten “relevant traits that detail habitat preference” it is a stretch of credulity to suggest that ancestral state reconstruction could meaningfully predict the status of characters such as rhamphothecal lamellae in what is a marginally fossil, even by Green River standards.

The validity of another study focused on morphology is not relevant to this study. Morphology based phylogenies are still relevant and critical to gaining a better understanding of avian phylogeny, especially since molecular-based studies are unable to include extinct taxa other than to use them as divergence dates. Morphological datasets are the only method that will allow us to understand how extinct dinosaurs evolved into 10,000+ species of living dinosaurs. The ancestral states within this study were carefully created and rigorously scored, and we note how they could be improved by future workers. Throughout the manuscript we state that this is a new dataset and that although it took years of work, many museum visits and reassessment of large amounts of extinct taxa via large amounts of specimens, it should be improved upon by future workers and is above all a necessary foundational dataset for better understanding dinosaur evolution.

Unfortunately, I could not verify many of the most important character descriptions because the data were not made available to me. The authors state that the data will become available on Morphobank and Dryad following acceptance and supply of a manuscript number. This is inconsistent with the registration of LSIDs in which the paper was reported to be “in press” in PloS One (notably before I received a request to review it).

ALL MATRIX DATA WAS MADE AVAILABLE TO REVIEWERS VIA MORPHOBANK USING A REVIEWER ONLY LOG-IN. The data will also be made publicly available upon acceptance for publication. The Zoobank Identifier was added prior to review as is requested by most publishers, and “in press” was the only option available on the website other than “published.” We chose to use the “in press” option to indicate that it was not yet published in a peer reviewed journal. The journal can be changed if needed.

One of my biggest concerns is whether the name Paakniwatavis is available or preoccupied. The authors used the name, together with a full description of the fossil in a preprint that appears on BioRxiv. Unrelated to the present manuscript, in May 2022 I inquired with the Secretary of the ICZN whether this could be done. I was advised not to do so. In December 2022 I became aware of a preprint of the present manuscript on bioRxiv in which the name was erected and assigned an LSID. There are only two other LSIDs associated with BioRxiv registered on Zoobank. Both are published works. I queried the Administrator of ZooBank whether Paakniwatavis was preoccupied or a nomen nudum. His answer was that it is unclear. This is his response:
“As for bioRxiv being pre-publication by definition, this is something the Commission has discussed and has not yet resolved. Art. 9.9 says that works cannot be published as “preliminary versions of works accessible electronically in advance of publication”. However, this Article is very problematic because it simply says that a work cannot be published until it is published. And technically, fulfilling all the requirements of the Code for a published work means it’s published. If an electronic work fulfills all the criteria for publication (e.g., prior registration in ZooBank, evidence of registration within the work itself, online archive and ISSN indicated in ZooBank, etc.), then it is published in the sense of the Code. The main reason why bioRxiv would fail the criteria for publication would be Art. 8.1.1: “it must be issued for the purpose of providing a public and permanent scientific record”. So, if bioRxiv is not explicitly indicated as issuing PDFs for the purpose of providing a public and permanent scientific record”, then it would fail to serve as a venue for Code-compliant published works. However, there is no guidance in the Code on how one assesses whether a given PDF is issued for the purpose of providing a public and permanent scientific record. Unless bioRxiv explicitly states that this is not it’s purpose, one could argue that it is intended for the purpose of providing a public and permanent scientific record. … In summary, nothing has yet been published in the sense of the ICZN Code within bioRxiv. And there is some debate whether anything *could* be published therein (based on interpretations of Art. 8.1.1, and Art. 9.9). … as there is no clear answer for how to interpret Art 9.9 (the rule that prevents publication as “preliminary versions of works”). Thus, it’s somewhat ambiguous whether names created within a “pre-publication” can be Code-compliant – depending on how one interprets Art. 9.9 and Art. 8.1.1). … This is a really interesting case, because technically, by a very narrow interpretation of the Code (which I doubt most Commissioners would agree with), it fulfills the requirements of the Code, unless the PDF version available on bioRxiv (https://www.biorxiv.org/content/10.1101/2022.11.23.517648v1.full.pdf) fails Art. 8.1.1. I will need to share this example with the Commission to see if there is any way it could be considered published in the sense of the Code as a PDF available through bioRxiv. I will need to get back to you on this.” [He has not, but in a subsequent email wrote:] “I’m still drafting the email to the Commission to examine this case. I also just noticed your email to me from two days ago – VERY sorry I missed that! I would have replied sooner had I not missed it! Indeed, this is a very interesting case.”

Reviewer 2 stated that they contacted the Administrator of ZooBank whether *Paakniwatavis* was preoccupied or a nomen nudum since the name was already used in our preprint publication on the new species, which was processed by PLOS ONE and bioRxiv. I am concerned that by reaching out to ZooBank to discuss my paper, Reviewer 2 has violated the nondisclosure agreement of PLOS ONE, and that this further maligns our work outside the context of this review. Reviewer 2 could have instead focused their efforts on reviewing the data available, but instead they went outside of the scope of this paper review to make an issue of something that is standard practice.

Furthermore, many other workers have published preprints that include new species names as it encourages transparency and open access in science. This reviewer is suggesting that all new taxonomic names that are published in a preprint are not valid, which would have broad and untenable implications for all scientific publications that include a new taxonomic name.
