# Supplementary material for: A new Paleogene fossil and a new dataset for waterfowl (Aves: Anseriformes) clarify phylogeny, ecological evolution, and avian evolution at the K-Pg Boundary
Source: PLoS One. 2024 Jul 30;19(7):e0278737. doi: 10.1371/journal.pone.0278737 (PMC11288464; doi:10.1371/journal.pone.0278737)
Supplement: Attachment — Submitted filename: April PLOS 1 Anseriform response to reviewers.docx [file pone.0278737.s014.docx]

**Response to Reviewers**

**In response to DRYAD query from the Editor:** We apologize for this mistake. We have now uploaded all 511 files. For some reason some of the files had not uploaded.

Also clarification added to methods section: “We trimmed the number of resultant files from a two-part CT scan (n=2000) to those that contained the fossil (n= 511), and resliced the scan in imageJ so that the fossil is visible in lateral view.”

Originally 2000 projections were created for the scan, but due this fossil requiring a two-part multiscan because of its size, over half of the projections contained nothing but air. We trimmed the number of projections to the number that contains the fossil, which is 511, and resliced the scan in imageJ so that the fossil is visible in lateral view.

Link to slices: <https://datadryad.org/stash/share/z_tvwBWTt5tXk9pQmCWclxGWV-7bKlR0SuTpztro_tc>

Reviewer #1: I am satisfied to see that the authors finally addressed some of the key issues raised in previous review and only have a few final points that should easily be addressed:

Thank you, we appreciate your thoughtful comments.

- You have noted that you included Mayr et al. 2023 in your revised description, but neither has the paper been cited nor do I find any reference to it. This is unfortunate, because Mayr et al. (2023) figured and described various elements that were not figured by Houde et al. (2023) and that could be used to differentiate your new taxon (e.g., there are differences in the lengths of the pedal phalanges).
In the comparisons in line 362 ff. you seem to refer to the coracoids figured by Mayr et al. (2023) and this should be mentioned, because in none of the cited publication the coracoid of the taxon has been figured.

Thank you for noticing this oversight. The citation has now been added in the description and in the References Section, and further comparisons have been added.

- In the systematic heading: please provide the author/publication year for Anseriformes and format the heading according to the other headings

This has now been revised.

- Throughout the manuscript: it is "impressio musculi"/"impressio m." (not "impression musculi")

This has now been corrected.

- lines 239 and 361 (and perhaps elsewhere): write Paakniwatavis in italics

This has now been corrected.

- line 364: Perplexicervix (not Perplexicervis)

This has now been corrected.

Reviewer #2: I congratulate the authors for making numerous significant improvements in the current revision, particularly with regard to Figure 5. I hope that it is evident from the effort I have invested to make even minor editorial corrections, which themselves could not possibly influence a final decision, that my criticisms have always been intended to be constructive. Paakniwatavis is an important fossil, deserving of the best possible description. I hope the authors and Editor(s) take this to heart as they read my comments below. I apologize in advance for the length and some redundancy. This is a long and complicated paper that warrants it.

Thank you for your very detailed comments. There are some points on which we disagree and do not appear to be able to reach consensus. As you note, these do not influence your decision on the merit of the paper and we hope that the manuscript can now move forward.

Please refer to attachment for remainder of review.

—-------

REVIEWER 2 ADDITIONAL RESPONSE:

Reviewer #2: Review of PONE-D-22-32093R3 A new Paleogene fossil and a new dataset for

waterfowl (Aves: Anseriformes) clarify phylogeny, ecological evolution, and avian evolution at

the K-Pg Boundary.

I congratulate the authors for making numerous significant improvements in the current revision, particularly with regard to Figure 5. I hope that it is evident from the effort I have invested to make even minor editorial corrections, which themselves could not possibly influence a final decision, that my criticisms have always been intended to be constructive. *Paakniwatavis* is an important fossil, deserving of the best possible description. I hope the authors and Editor(s) take this to heart as they read my comments below. I apologize in advance for the length and some redundancy. This is a long and complicated paper that warrants it.

I will address the easiest and least important things first.

line 82: replace period with comma or preferably begin new sentence

This has now been corrected.

line 201: most ibises and rails are semiaquatic, so why aren’t the “ibis-like” bird and Messelornis mentioned along with the aquatic *Limnofregata* and *Presbyornis* (line 94)? *Presbyornis* with its ibis-like long legs was certainly a semiaquatic wader, rather than strictly aquatic.

We now mention them and list them as being semiaquatic as requested.

line 203: No study since the 2012 one cited, of which there are many, has recovered any Early Eocene fossil whatsoever as a stem parrot. They have all been recovered as stem Psittacopasseres, i.e., sister to Psittaciformes+Zygodactylidae+Passeriformes.

We have changed this statement to “stem Psittacopasseres” as requested

line 282: add space to processof

This has now been corrected.

line 285: The projection dorsal to the medial angle of the coracoid is part of the origin of the sternocoracoclavicular ligament

This has been reworded this as requested.

lines 239, 300: italicize Paakniwatavis

This has now been corrected.

line 304 uncapitalize It

This has now been corrected.

lines 305, 838, 851: add space after period

This has now been corrected.

line 325 “skewed cranially” is not in opposition to “flat”, so “but” should be replaced with “and”

This has now been reworded for clarity.

line 353: that, not those

This has now been corrected.

lines 358, 399-401, 594 and elsewhere: I think the dependent clause (“as in…”) is supposed to be separated by a comma, as done elsewhere, e.g., lines 353, 494, and 878

This has now been corrected.

line 380: the sentence is redundant since the retroarticular could only be present if preceding sentence states it is recurved

This has now been reworded.

line 642 please clarify which “instances” produced which results

These paragraphs have now been revised and new Supplementary Figures have been cited and uploaded to Morphobank.

line 1014 it appears that something is missing between stem Recovery

This has now been fixed.

I was compelled to see the influence of deleting a specific character (the length of the postorbital process) since I could not verify its existence from the images provided. In so doing, I discovered errors in the scoring of taxa in the data matrix and in the numbering of character descriptions in the Appendix based on comparison to the characters listed in the data matrix.

For example, character 424 in the Appendix is mislabeled 427, which itself is listed twice, and number 423 is duplicated with no description.

Neither the scoring of character 424 nor 427 in the data matrix appears to match their description, either with regard to their distribution among taxa or the numbering of the states.

Character 271 is misnumbered as 277, and 277 is misnumbered as 283.

Characters 268-288 in the Appendix are numbered in this order: 268, 269, 270, 277, 272, 273, 274, 275, 276, 283, 278, 285, 2138, 281, 288, 284, 285, 286, 273, 287, 288.

We thank the Reviewer for their attention to detail here. The Appendix has now been renumbered. Only the numbering on the Appendix was incorrect; the characters were also present in the same, correct order with correct numbering within the Mesquite file.

**The body of the furcula of *Paakniwatavis* is described as being thick (character 424), and scored the same as Anhimidae and *Anseranas*. This is untenable; consult Figure 3.** On the other hand, *Anachronornis* and Danielsavidae are scored as unlike all other taxa, which is again untenable based on figures in Houde et al 2023 and Mayr et al 2023. The available states for furcula thickness are intermediate and thick but there is no option for thin, which would clearly apply to many if not most Galloanseres.

A “thin” state is not present here as this character was taken from the Neoaves focused dataset of Musser and Cracraft 2019 that is continuing to be iteratively expanded (eg. in Musser and Clarke 2020). Our thin state is only reserved for exceedingly sender furculae. When compared to some Neoaves, all taxa within the current matrix for this manuscript have either intermediate/typical furcula width or thick furcula width, therefore the character states were condensed for the purpose of this dataset. We also included 2 additional characters within this dataset (listed below) that further detail the width of the furcula along the scapus clavicle (435 and 436) that add further nuance but are not mentioned by the referee.

424. Scapus clavicle, mediolateral width: intermediate (1); extremely thick (2). Musser and Clarke [83], character 422; Ericson [14], character 44.

435. Scapus clavicle, width compared to that of acromion and acrocoracoideus region: more narrow (1); subequal (2); larger (3).

436. Scapus clavicle, width of lateral margin compared to that just lateral to symphysis: more narrow (1); subequal (2); larger (3).

The postorbital process of crown Anseres is unique among all know fossil and extant birds with regard to its length and orientation (characters 51, 52, resp.), serving as an attachment for the suborbital ligament that is peculiarly hypertrophied in crown Anseres (or ossified in parrots and some shorebirds). Most of the taxa in the matrix, e.g., *Presbyornis*, are mis-scored at least for length (character 51). Although superior published images can be found elsewhere, (see below), this can be easily appreciated simply by comparing the two crown Anseres, *Anseranas* and *Chloephaga* (Figures 3D, E, resp.), to the screamer and *Presbyornis* (Figures 3C, F, resp.). These are examples that I just happened to find while investigating just three characters of interest. That is not to say that there are either more or no others. I have not proofread the Appendix or verified other character scores.

Character 51, is scored by comparing the length of the postorbital process to the length of the quadrate. I (Musser) have listed both characters below. I double checked the scorings of both characters and have found them to be correct. I have included photographs of *Presbyornis*, *Anseranas*, *Chloephaga*, and *Anhima* below, which are all scored as having an elongate retroarticular process in our matrix. The postorbital process is elongate in both Anhimids and *Presbyornis.* Please note that the angle of the postorbital process relative to the skull makes the *Presbyornis* postorbital process appear shorter in the photo than it is. It is best viewed in the scan, but I am not permitted to share NMNH scans to non-NMNH employees without proper paperwork.

In regards to the study of *Presbyornis*, I have accessed detailed CT scans of the skull of *Presbyornis* from NMNH and have carefully studied the skull in person along with all other *Presbyornis* material available at that Museum over the length of 3 months during an NSF GRIP internship. I am unaware of superior images *Presbyornis* that may exist; in my experience this fossil is not very well figured or described, and that wone of the primary reasons that I became an intern at NMNH and was able to access these scans.

51. Postorbital process, length: truncate (1); extremely elongate, subequal in length to height of quadrate (2). Musser and Clarke [83], character 54; Musser and Clarke [77], character 26; Mayr and Weidig [52], character 2; Ericson [14], character 2.

52. Postorbital process, orientation of apex: facing rostroventrally (1); facing ventrally (2). Musser and Clarke [83], character 55; Mayr and Weidig [52], character 2.

*Chauna*:

*Chloephaga*:

*Presbyornis*:

*Anseranas*:

I executed parsimony searches on the dataset with and without *Wilaru*, *Anachronornis*, *Danielsavis*, NMS.Z.2021.40.2, and merged_ *Danielsavis*, individually and in combinations. The various permutations produced a variety of results unworthy of describing in detail, but some important generalizations can be made. Foremost, their complete exclusion resulted in a single tree that did not differ from constraint trees. In other words, the data fit the topology with near perfection (I am not implying that CI was 1.0) for those taxa that the authors scored in the original matrix. As the authors themselves note, the inclusion of *Wilaru* wreaked havoc. They attributed this to missing data, so they deleted it from further analyses. Similarly, *Anachronornis* wreaked havoc on the tree, regardless of which of three constraint trees or none were enforced. Typically among these, either Palaeognathae, Galloanseres, or Anseriformes, or some combination of them were recovered as nonmonophyletic. *Anachronornis*, *Gastornis*, and pelagornithids were recovered at a variety of depths of the tree. In the Galloanserine_constraint++ constraint tree, *Paakniwatavis*, *Chaunoides*, and *Nettapterornis* formed a clade within an inclusive clade of fossil taxa excluding *Gallinuloides* and fossil paleognaths. Curiously, merged_*Danielavis* alone joined Anseriformes whereas *Anachronornis* alone did not. This result is difficult to explain because it is Danielsavidae, not Anachronornithidae, whose anseriform relationships have been questioned. To me, the take home message is that the process of choosing characters to score is a subjective one. This is evident in the difference between the relatively low consistency of randomly sampled molecular data compared to the very high consistency of many morphological data sets. It is also evident in the extraordinarily high consistency of different morphological data sets that produce conflicting trees with very high confidence. Published differences in the recovery of *Vegavis*, Pelagornithidae, presbyornithids, and others pertinent to this study are all excellent examples. A researcher defines characters based on what they see in front of them, and whether consciously or not, this may be guided by preconceptions of relationships.

It is very difficult for me to accept that analyses omitting *Wilaru*, *Anachronornis*, *Danielsavis*, NMS.Z.2021.40.2, and merged_ *Danielsavis* produce trees that are completely consistent with constraint trees based on extant taxa, when others (e.g., Field et al 2020) have been unable to achieve similar conformity in their results without enforcing constraints (in fact, resulting in trees with significantly different topologies for some of the same taxa using in this study, e.g., monophyly of *Presbyornis* and *Wilaru* as proposed by previous authors).

The papers referred to here used constraint trees based on the molecular data. We employed a Galloanserine backbone constraint based on molecular data within the morphology only analyses, and within the combined analyses included the molecular data on which these constraints were based. Thus this is not surprising but indeed expected.

As detailed above and further below, I have concerns about the data set that has formed the basis of the phylogeny and all subsequent inferences herein. Researchers may always disagree about the scoring of some characters, but there are clear errors in the data matrix.

As noted above, formatting errors within the Appendix resulted in character numbers not aligning with the Mesquite file numbers but the actual characters are the same and are correctly scored. The numbering in the Appendix has now been fixed but does not affect the analyses. We double checked scorings of all characters questioned by the Referee and have provided photographs.

Also, an appreciable number of characters are scored as missing for *Anachronornis* and danielsavids that I believe could be scored from published illustrations. At the very least, the data set needs to be carefully verified.

We were conservative in scoring these new taxa published in 2023 you asked us to add because we have not seen the specimens in person yet, and one of the specimens is still being actively prepared by Peter Houde and has not been made available for examination.

The Appendix, on which the reader depends for the definition of character states, must to be proofread and corrected, as needed.

We have now corrected the Appendix numbering and have proofread it. All numbering within the Mesquite file remains correct.

All comments below begin as quoted from my review of the previous manuscript version identified by line number of that version, followed by the authors’ response in red, and last my current review, respectively.

59-60: citation 11 recovers Pelagornithidae as neoavian, not anseriform, as does more recent

analysis by Benito et al, cited elsewhere in the manuscript but not cited here. The disagreement

among analyses is discussed much later (678-692) but is misrepresented here that there is no

disagreement. It should be.

Citation 11 has now been removed from this instance.

Deleting this reference does not address my assertion that the uncertainty about the relationships of pelagornithids should be stated here in the Introduction, where they are first included in “this clade” (line 58), “Anseriformes” (line 55). At best, they were recovered by Bourdon 2005 as sister to Anseriformes (in a non-monophyletic Galloanseres, which of course is a non-starter), and in the parsimony but not Bayesian analyses of Field et al 2020 Nature 579. My reference above to Benito et al was a mistake. I meant Field et al.

Thank you for the clarification, as Benito et al. do not include Pelagornithidae within their analyses.

We agree that the relationships of Pelagornithidae remain uncertain; however, they have most frequently and most robustly been recovered as stem Anseriformes.

This is even true within Field et al. 2020–please see Extended Data Fig. 9 below. Pelagornithidae are recovered as stem Anseriformes in the results of the parsimony analysis.

They are recovered within a polytomy containing Galloanseres, Neoaves and Vegavis in the results of the tip-dated Bayesian analysis.

In addition, Field et al. 2020 only included *Cariama*, a shorebird and two rails to represent all of Neoaves, which makes up 90% of birds–therefore, this is not a good sample to test the phylogenetic relationships of Pelagornithidae with.

Similarly, Mayr et al. 2021 recovered Pelagornithidae in a polytomy containing Neoaves and Galloanseres (see Figure 8 below). We agree with the Referee that the relationships of Pelagornithidae require further phylogenetic testing.

From Field et al. 2020:

Caption for above Figure: a, Results of the parsimony analysis. *Asteriornis* (pink) resolves as the sister taxon to crown Galloanserae. b, Results of the tip-dated Bayesian analysis with a soft-maximum neornithine root age of 86.5 million years. An estimated timescale is shown on the *x* axis, although see caveats relating to divergence times in the [Supplementary Information](https://www.nature.com/articles/s41586-020-2096-0#MOESM1). *Asteriornis* (pink) resolves as the stemward-most member of Pangalliformes. Colours match those in Fig. [3](https://www.nature.com/articles/s41586-020-2096-0#Fig3) and extinct taxa are denoted with daggers. See [Supplementary Information](https://www.nature.com/articles/s41586-020-2096-0#MOESM1) for full details of phylogenetic analyses, character information, synapomorphies of key clades, support values and tree files.

From Mayr et al. 2021:

62: Anhimidae are aquatic foragers, not terrestrial except maybe in captivity

Reworded, but still incorrect. Southern and Horned Screamers are seasonally terrestrial (they are pests to crops), semiaquatic otherwise, while Northern Screamers are semiaquatic or arboreal year-round. To the best of my knowledge, they all nest on water and their chicks swim. Consult del Hoyo or regional field guides. Perhaps it would be best to refer to them as “semi-terrestrial/semi-aquatic”.

We have changed the wording to this: “Extant Anseriformes include the largely terrestrial Anhimidae that are primarily herbivorous, semiaquatic foragers…” More study by other workers of Anhimidae is necessary to better classify them.

Here is what we found from del Hoyo (HBW online):

## “Diet and Foraging

Almost entirely herbivorous, screamers forage mostly by grazing on leaves, flowers, seeds, and other aquatic vegetation. They occasionally dig in the mud for food as well, although it is not yet clear what they seek.

103, 227: Mayr et al (2023; https://doi.org/10.26879/1301; not cited) vitiated the suggested

anseriform relationship of *Perplexicervix*

The wrong citation is added, i.e., Mayr 2010 vs. Mayr et al 2023. Mayr 2010 remarks on similarity to Anseriformes. Mayr et al 2023 specifically debunks that potential relationship, suggesting instead a relationship to Otidiformes, and that point is still not made. In fact, why *Perplexicervix* is compared to *Paakniwatavis* at all is not mentioned anywhere.

The reviewers initially requested that *Perplexicervix* be compared to *Paakniwatavis* as it was originally thought to be anseriform, as stated in Mayr 2010.

Mayr et al. 2023 remarked on a few similarities of the skeleton of *Perplexicervix* to those of members of Otididae but did not perform phylogenetic analysis that included this taxon, and the neoavian-like traits that the authors referred to are not pictured. Furthermore, like Galloanserines, *Perplexicervix* exhibits basipterygoid processes–these are absent in most of Neoaves.

Within the Mayr et al. 2023 paper it is even stated that “unambiguous character evidence for a phylogenetic placement of *Perplexicervix* has yet to be identified.” Therefore, we feel it best to include comparisons between *Paakniwatavis* and *Perplexicervix* within the manuscript, and only include it within analyses presented in the Supplementary data.

We recover *Perplexicervix* as a stem anseriform using morphology only data and parsimony analysis without molecular backbone constraints enforced. When molecular backbone constraints are enforced, we recover *Perplexicervix* with a polytomy containing *Ichthyornis* and the rest of Aves. This can be seen in the newly added Supplementary Data Figures S4 and S6 in Morphobank under the Supplemental Figures Folder. We have also included a PAUP log for all analyses including the taxa requested by the Referees.

283-288: the description goes from carpometacarpus to humerus back to carpometacarpus. This

disorganization was commented on in a previous review.

Revised here, but not elsewhere. In comparison with *Presbyornis*, the Differential Diagnosis goes from the skull (beginning line 239) to postcrania (beginning line 245), then back to the skull (beginning line 259), then back to the postcrania again (beginning line 268, including the humerus a second time). In the paragraph beginning on line 290, the furcula and coracoid are named in the first sentences that refer to parts of them, but it is not until the second and third sentences referring to parts of the humerus that it is named. Other parts of the manuscript are similarly disorganized. For example, *Telmabates* was referred to Presbyornithidae (but see de Mendoza et al 2024 Journal of South American Earth Sciences). Why do comparisons begin with *Presbyornis* (beginning line 239), but comparisons to *Telmabates* (beginning line 304) follow comparisons to *Anachronornis*, *Danielsavis*, and *Perplexicervix* (beginning line 277), which are phylogenetically far removed from *Presbyornis*?

Thank you for catching this. We have now revised the order as requested. Telmabates does not have a skull available and only has a few elements (largely limbs and a few vertebrae) available for comparison. We compare the new taxon to the taxa that have the most complete skeletons first.

330-331: I have been provided with no image to verify that the postorbital process was long as

claimed. It would be surprising if it was long, since it is not in either anhimids or *Presbyornis*.

This was commented on in a previous review.

The postorbital process can be seen in Fig 3 (B1 and B2, shown below) and in the CT slices in the supplement.

The editor can see pictures included in this response.

I cannot discern any bones at all in any and all of the tiff CT slices, only the rock slab and the cracks within it, despite attempted adjustments using imageJ. So, I cannot accept the description of the postorbital without being able to see it in them or in Figure 3B. (I also cannot verify that the medial side of the mandible is concave, line 265, or that the sternocoracoid fossa is deeper than “the shallow impression in *Danielsavis*”, line 284. Note that Mayr et al 2023 treated NMS.Z.2021.2 as probably conspecific with *Danielsavis* nazensis, and it has a deep sternocoracoid fossa.) My point about the absence of a long postorbital process in anhimids and *Presbyornis* is based on the mechanics of cranial kinesis and rapid jaw and tongue motion (see Zweers and Vanden Berge Netherlands J Zoology 47; Zelekof and Stidham 2018 Zoologicheskiĭ Zhurnal 97). Lacking a wide bill or other evidence of filter feeding, it would be hard to explain the presence of an elongate postorbital process in *Paakniwatavis* without function. It would also be surprising based on phylogeny since this character is unique to crown Anseres, as nicely illustrated in Figure 3D, E vs 3C, F. In contrast, the postorbital is short in the only stem Anatidae/Anseriformes (i.e., *Conflicto*, *Presbyornis*, and *Anachronornis*) in which it is preserved. *Anachronornis* is scored as ?, even though its short postorbital is clearly evident in Houde et al 2023. *Presbyornis* is incorrectly scored as possessing a long postorbital process. It doesn’t. I base this on both direct observation and published images (e.g., Olson and Feduccia 1980; Houde et al 2023; Field et al 2020), not to mention Figure 3F in the present manuscript, and Musser and Clarke’s definition of truncate vs long character states relative to quadrate height. The fact that *Presbyornis* is incorrectly scored herein as possessing a long postorbital process calls into question the scoring of this character in *Paakniwatavis*, as well. If truly long, this character could be influential in attracting *Paakniwatavis* to crown Anseres (i.e., *Anseranas* and crown Anatidae) to the exclusion of Anhimidae in phylogenetic analysis. Re-running the parsimony analysis without this character does not recover a significantly different tree (without Wilaru, *Anachronornis*, Danielsavidae, and *Perplexicervix*; the postorbital of the latter two are unknown), but see above.

We have now provided three cut through videos of the CT slices on Morphobank in the CT Data folder within the Supplementary Data so that the Referee may more easily view them. We have also included additional screenshots of the slices in question below with heightened contrast. Please see above comments and photographs regarding the scoring of the postorbital process.

Retroarticular process:

Postorbital process:

517: segmented files are not available on MorphoBank as claimed. There is one obj file of the

quadrate and one stl file of a tarsometatarsus, nothing more. None of the descriptions and

character scoring based on segmented CT scans other than these two are verifiable by readers.

They need to be.

These are the only segmented files that we created. When we tried to segment the rest of the

skeleton it was too crushed to be useful. The rest of the characters are visible in the slices that are

on DRYAD—unfortunately in crushed specimens like this, segmenting does not usually offer

clarity into character states as much as the CT slices do. An OBJ file is a different version of an

STL file but can still be viewed as a mesh—it is still the segmented portion of the skeleton.

In light of crushing and the inability to generate 3-dimensional images, I do not understand how it can be inferred that “The mandibular ramus caudal to the rostral fenestra mandibulae is deep and concave along the medial face in *Paakniwatavis*” (lines 264-265).

It is not necessary and is not always possible (as in this case) to segment elements from a CT scan, but anatomy can be discerned from careful study of the CT slices.

521-526: There are only 41 taxa included in the nex files in MorphoBank. In other words, the

matrix provided does not include *Anachronornis*, *Danielsavis* and *Perplexicervix* as claimed. Just

as importantly, the matrix includes no unambiguous Neoaves. The importance of this is that there

are no data included to challenge the recovery of Pelagornithidae within Galloanseres, which is a

viable and the most current hypothesis of their relationships.

We have included a version of the dataset on MorphoBank that includes *Anachronornis*,

*Danielsavis* and *Perplexicervix* .

A “version of the dataset on Morphobank that includes *Anachronornis*, *Danielsavis* and *Perplexicervix* ”? Why does what I downloaded not include these? Is it even possible that I am somehow linked to an obsolete but persistent Morphobank submission with the same identifier? I look forward to your future analyses. In the meantime, my comment about the absence of any representative Neoaves in the present study stands with regard to the recovery of pelagornithids and *Perplexicervix*, the latter to which Mayr et al most recently liken to Otidiformes. If these are relevant to *Paakniwatavis*, then so are their potential relationships within Neoaves.

We included this matrix in the Data Matrices folder in Morphobank (see screenshot below). We did not include Neoaves as there is still debate as to which Neoaves are the most basal and as discussed above, it is out of the scope of this paper. Please see our above discussions regarding the possible positions of *Perplexicervix* and Pelagornithidae.

571: In a previous review, objection was raised about the inclusion of the PCA and LDA

analyses on the grounds that they recovered results known to be incorrect even for neotaxa. The

authors dismissed this concern. They stated that they agreed and therefore moved these analyses

to the Supplementary Data. Maybe so fig S4, but in fact they retained these in the Methods and

in the Results section 778-794 and the results are alluded to in the Discussion in the form of

interpretations. It should be deleted altogether.

We are not going to delete this section.

The analyses were included to show that we attempted to reconstruct results for our added taxa

but that it was not possible, and is very thorough in describing this and letting readers know

where they can further scrutinize these in the supplement. We think that it is important to include

so readers have all available information on performed analyses.

…and…

778-794: this paragraph really needs to be deleted, as does figure S3, as articulated in previous

review. Even the authors concede “the results are largely inconsistent with other features or the

known behaviors [of neotaxa] (i.e., *Chauna*)”. It is nonsense, so don’t include it.

We feel that it is important to include so that other workers do not waste time replicating these

analyses for future studies, as it is one of the only datasets available for PCA and LDA studies of

these taxa.

There may be many analyses that would be appropriate to explore (especially in a graduate thesis) that are, nevertheless, not worthy of inclusion in peer-reviewed publication. In my opinion, this is one.

The figures and mentions of analysis have now been deleted.

If my memory serves me correctly, I stated in my review of the original submission that the ancestral state reconstruction is over-interpreted.

The fact that the paragraph on line 779 begins “Ancestral state reconstruction for behavioral characters” but then goes on to include morphological ones in the same sentence is misleading. Whereas it is understood that behavioral characters must be inferred, it can be misconstrued that morphological characters are observed in the fossil.

The wording has now been revised for clarity.

In fact, the statement “character state confirmed through scoring” adds to that confusion.

This was unclear and has now been removed.

Elsewhere in the manuscript (lines 978-980), some of these inferences are stated as matter of fact. Once published, they are destined to be propagated as such.

Reworded.

The statement that pelagornithids were non-swimmers based on this ancestral state reconstruction (line 790) is contradicted by their morphology. The vanishingly few pelagic piscivores that do not typically swim, i.e., tropicbirds and frigatebirds, are immediately recognizable by their uniquely diminutive feet.

Here, we are simply reporting the results of the ancestral state reconstruction in Mesquite. These are simply the results that the data provided; as stated in the manuscript further study of these taxa is required (and preferably more fossils need to be found) to create more robust hypotheses. However, this dataset and its ancestral state reconstruction is a foundation for that future work and thus should be described.

Inferred behavior and characters based on ancestral state reconstruction are elsewhere stated as matter of fact. For example, beginning line 779 “Ancestral state reconstruction for behavioral characters using the combined results phylogeny suggests that *P. grandei* likely preferred an aquatic or semi-aquatic environment, was either not specialized for aquatic feeding modes (hereafter referred to as a “non-swimmer”) or was a surface swimmer …” But later on line 979 the text states “*P. grandei* represents an early Eocene lacustrine, aquatic taxon that swam”.

This has now been corrected.

I strongly suggest that you state “Ancestral state reconstruction for behavioral characters using the combined data” rather than “combined results phylogeny” (line 779), and “Ancestral state reconstruction for behavioral characters using the morphological data” rather than “Ancestral state reconstruction for behavioral characters using the morphological results” (line 803). I had to read the latter several times before I could make sense of it.

This has now been reworded as requested.

575: *Nettapterornis* is labeled *Anatalavis* in the PCA figure S4.It still needs to be corrected on line 334 (Figure 4 caption)

This has now been corrected and the PCA figure has been deleted.

582-584: the authors state in their response to reviewers that *Paakniwatavis* is recovered as sister to Anseranatidae plus Anatidae, but here it clearly states “*Paakniwatavis* … is recovered across all analyses as the sister taxon of Anseranatidae.” What’s more, if sister to Anseranatidae plus Anatidae, then in fig. 5 Anatidae includes Vegavis, *Presbyornis*, *Conflicto*, *Telmabates* , and *Nettapterornis*. That is going to raise some eyebrows.

This sentence, and others have now been corrected to mention its recovery as an outgroup of Anatidae (Figure 5). We do not recover these taxa in Anatidae that is a referee error of reading the figure- we make this clearer by labeling Anatidae in this figure.

The text still says “Both the combined and morphological analyses recover a *Gastornis* (*Pelagornis*+*Protodontopteryx*) stem anseriform clade (<50% bootstrap support, 99% clade credibility) and a clade as sister taxon to Anatidae containing *Telmabates*, *Presbyornis*, *Vegavis*, *Conflicto*, and *Nettapterornis*” (lines 713-716). In other words, Anatidae includes the fossil stem taxa. I think the authors should refer to this as a Pan-Anatidae that includes stem anatids.

Thank you for the suggestion, we have now reworded this for clarity. We agree that Anatidae does not include these taxa. They are recovered as sister to crown anatidae (Anatidae now labeled in figure 5).

688-709: the Results section should report the results of this study, but it includes considerable

text reviewing the results of other peoples’ analyses. Those ought to go into the Discussion.

We thank the Referee for the suggestion. Changed.

That sounds backwards. At the very least, your own results should be presented first before the reader is lost in complicated review of other studies, only to return to your primary results later (e.g., line 745).

We have now moved the discussion of other studies to the Discussion section as requested.

752-754: both some jacanids and anhimids have third digits that are longer than the

tarsometatarsus (specimens directly available to me, but consult Ridgeway for individual

variation). Both are renowned for walking on floating vegetation, not swimming (although

screamer chicks notably swim), for which the long toes are specialized. Ref 106 is irrelevant to

the authors’ point since it concerns claw curvature, not digital length.

Jacanids are Charadriiformes which are highly specialized and not within the scope of

this dataset. It is still not confirmed whether the long toes in Anhimidae are useful expressly for

walking on floating vegetation. Here we are discussing pedal proportions including the claw and

that is why we included Ref 106.

Anatomical and behavioral comparisons are made to a variety of taxa that are further beyond the scope of the dataset, e.g., non-avian dinosaurs and turtles (lines 904-906) and dromornithids (line 958), so the statement about jacanids seems a bit insincere. Regardless, comparison to them is absolutely pertinent because they and anhimids are specifically documented as relying on their long toes to support the birds while foraging on floating vegetation (e.g., del Hoyo). Reference 106 (line 789) concerns nothing but claw curvature, as is clear even from its title. It does not consider digit length at all. Its citation here is disingenuous.

Thank you, Ok, I see your point now–I was confused on what was meant by this comment in the previous review. I have now removed citation 106. I did not add the comparisons even though I agree that they are interesting points as this bird was likely a swimmer and not a semi-aquatic forager, and because I do not want to add too much speculation on the life history of this taxon to the paper.

778-794: this paragraph really needs to be deleted, as does figure S3, as articulated in previous

review. Even the authors concede “the results are largely inconsistent with other features or the

known behaviors [of neotaxa] (i.e., *Chauna*)”. It is nonsense, so don’t include it.

We have now deleted this and the PCA results as requested.

799-800: The relationships of Pelagornithidae and *Gastornis* are not tested in this study because

their purported neoavian sisters were not included in the dataset. It is meaningless to report

where they are recovered among taxa that do not represent the diversity of current alternate

hypotheses of their relatives. There are three ways to deal with this. First, add representative

Neoaves to the dataset and rerun the analyses. Most of these data are already scored in the

datasets of the studies from which the current matrix was derived. This would be a valuable

exercise even if some data were missing. Moreover, they can be part of a backbone constraint

tree. Second, Pelagornithidae and *Gastornis* could be deleted from this dataset and report. Third

and by far the least desirable, the authors could include a caveat that states that Pelagornithidae

and *Gastornis* are recovered as … but their proposed neoavian sisters (include refs) were not

included in this study. I realize the third option is the easiest for the authors because it requires

virtually no effort, but it is the poorest remedy if a true remedy at all.

They are tested to the fullest extent possible within the scope of this paper, because

Pelagornithidae are not neoavian and *Gastornis* has been hypothesized to be a close relative of

them and/or Anseriformes. Adding neoavian taxa is outside of the scope of this paper, especially

since there is still not a consensus for what the most basal group within Neoaves is. We have

added the third option as suggested by the Reviewer. Even if 3-4 neoavian taxa were added, this

would not be enough to test the relationships of all included taxa fully since a comprehensive

dataset for Neoaves is needed. Redoing the dataset would mean that the analyses, data and manuscript would need to be redone 1.5 years after submission of the paper.

I am satisfied with the caveats that the authors included (“the third option”) in the first paragraph of the revised Discussion. Nevertheless, I am compelled to comment on the authors’ response, above. The first sentence of this response stating that Pelagornithidae are not neoavian is an overstatement according to recent analyses that tested their relationships (e.g., Mayr et al 2019 Papers in Palaeontology 2019, Field et al Nature 579). The lack of any Neoaves is a serious flaw in this experimental design. Pelagornithids have been recovered as sister to neoavian Procellariiformes, and a single representative of that order is all it would take to test that in this analysis. Inclusion of a ‘most-basal’ neoavian is unnecessary, but flamingos are among those that have been suggested to be, and flamingos possess a hooked retroarticular that has been used in part to define crown Galloanseres.

Even the authors state on lines 380-381, “A retroarticular process is present as in all Galloanserines and included fossils”, but in fact it is lacking in Pelagornithidae (Mayr and Rubilar-Rogers 2010). Moreover, some birds referred to as stem Anseriformes had originally been likened to flamingos.

Whatever datasets Dr. Musser may be building for future analysis do not constitute evidence for statements made herein about the recovery of Pelagornithidae (plus Gastornithidae) as sister to Anseriformes, which will undoubtedly be cited as definitive statements by future authors.

Please see our above discussion regarding previously published results not recovering an more of relationship between Pelagornithidae and Neoaves than galloanseres and Aves. We have corrected the statement on lines 380-381 but the referee is wrong about some of these statements (see above)..

838-840: No, *Conflicto*, *Anachronornis*, and *Danielsavis* all have narrow bills and only

*Presbyornis* and *Nettapterornis* are known to have wide ones. Brief reference to *Anachronornis*

and *Danielsavis* is appended to the end of this paragraph (857-859) in what seems to be a

minimal effort for resubmission without actual revision that brings this manuscript up to date.

The bill of *Conflicto* is narrow and utterly unlike that of *Nettapterornis*. The bill of

pelagornithids is so manifestly different from that of Anseriformes (instead, like those of

neoavian Pelecaniformes and Procellariiformes, e.g., in the presence of rhamphothecal

segmentation as well as dimensions) that pelagornithids don’t warrant mention here.

The statement we explicitly discuss in this paragraph that *Conflicto* has is that its beak is

“mediolaterally wider than the width across the paroccipital processes,” and this is consistent

with the states in Anseranatidae and extant Anatidae.

The beak of *Conflicto* is broken along the sides and front, indicating that if unbroken the bill would have been wider than those of *P. grandei*, *Anachronornis* or *Danielsavis*.

We have now added *Anachronornis* and *Danielsavis* to the “narrow beak” discussion portion as suggested by the reviewer.

Pelagornithidae have been recovered as closely related to Anseriformes, and thus it is our opinion that it is important tocompare their beak shape to that of other Paleogene Anseriformes.

I stand by my original comment.

We respect your opinion but will have to disagree.
